# Solid‐Phase Synthesis and Biological Evaluation of Peptides ADP‐Ribosylated at Histidine

**DOI:** 10.1002/anie.202313317

**Published:** 2023-11-14

**Authors:** Hugo Minnee, Johannes G. M. Rack, Gijsbert A. van der Marel, Herman S. Overkleeft, Jeroen D. C. Codée, Ivan Ahel, Dmitri V. Filippov

**Affiliations:** ^1^ Bio-Organic Synthesis Leiden Institute of Chemistry Leiden University RA-2300 Leiden (The Netherlands; ^2^ Sir William Dunn School of Pathology University of Oxford South Parks Road Oxford OX1 3RE UK; ^3^ Current address: Medical Research Council Centre for Medical Mycology at the University of Exeter University of Exeter, Geoffrey Pope Building Stocker Road Exeter EX4 4QD UK

**Keywords:** ADP-Ribosylation, Glycosylation, Histidine, Peptides, Solid-Phase Synthesis

## Abstract

The transfer of an adenosine diphosphate (ADP) ribose moiety to a nucleophilic side chain by consumption of nicotinamide adenine dinucleotide is referred to as ADP‐ribosylation, which allows for the spatiotemporal regulation of vital processes such as apoptosis and DNA repair. Recent mass‐spectrometry based analyses of the “ADP‐ribosylome” have identified histidine as ADP‐ribose acceptor site. In order to study this modification, a fully synthetic strategy towards α‐configured N(τ)‐ and N(π)‐ADP‐ribosylated histidine‐containing peptides has been developed. Ribofuranosylated histidine building blocks were obtained via Mukaiyama‐type glycosylation and the building blocks were integrated into an ADP‐ribosylome derived peptide sequence using fluorenylmethyloxycarbonyl (Fmoc)‐based solid‐phase peptide synthesis. On‐resin installation of the ADP moiety was achieved using phosphoramidite chemistry, and global deprotection provided the desired ADP‐ribosylated oligopeptides. The stability under various chemical conditions and resistance against (ADP‐ribosyl) hydrolase‐mediated degradation has been investigated to reveal that the constructs are stable under various chemical conditions and non‐degradable by any of the known ADP‐ribosylhydrolases.

## Graphical abstract

We report the preparation of ribofuranosylated Fmoc‐histidine building blocks via a Mukaiyama‐type glycosylation. These building blocks were used in solid‐phase peptide synthesis, followed by on‐resin pyrophosphate construction and deprotection to access peptides containing N(τ)‐ and N(π)‐ADP‐ribosylated histidine. The N‐glycosidic linkage proved stable to the treatment with aqueous acid and base and resistant to (ADP‐ribosyl)hydrolases.

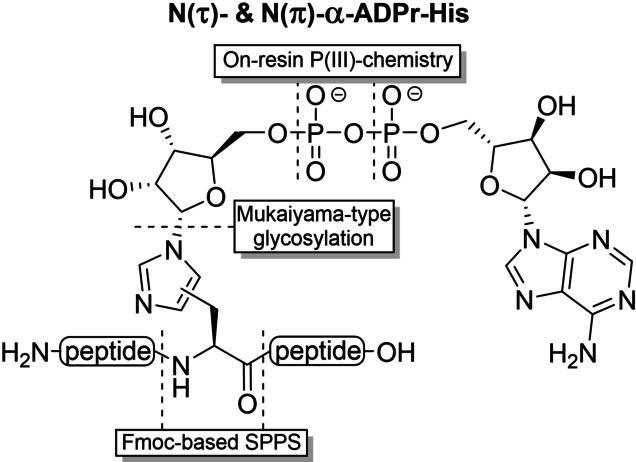

## Introduction

**Figure 1:**
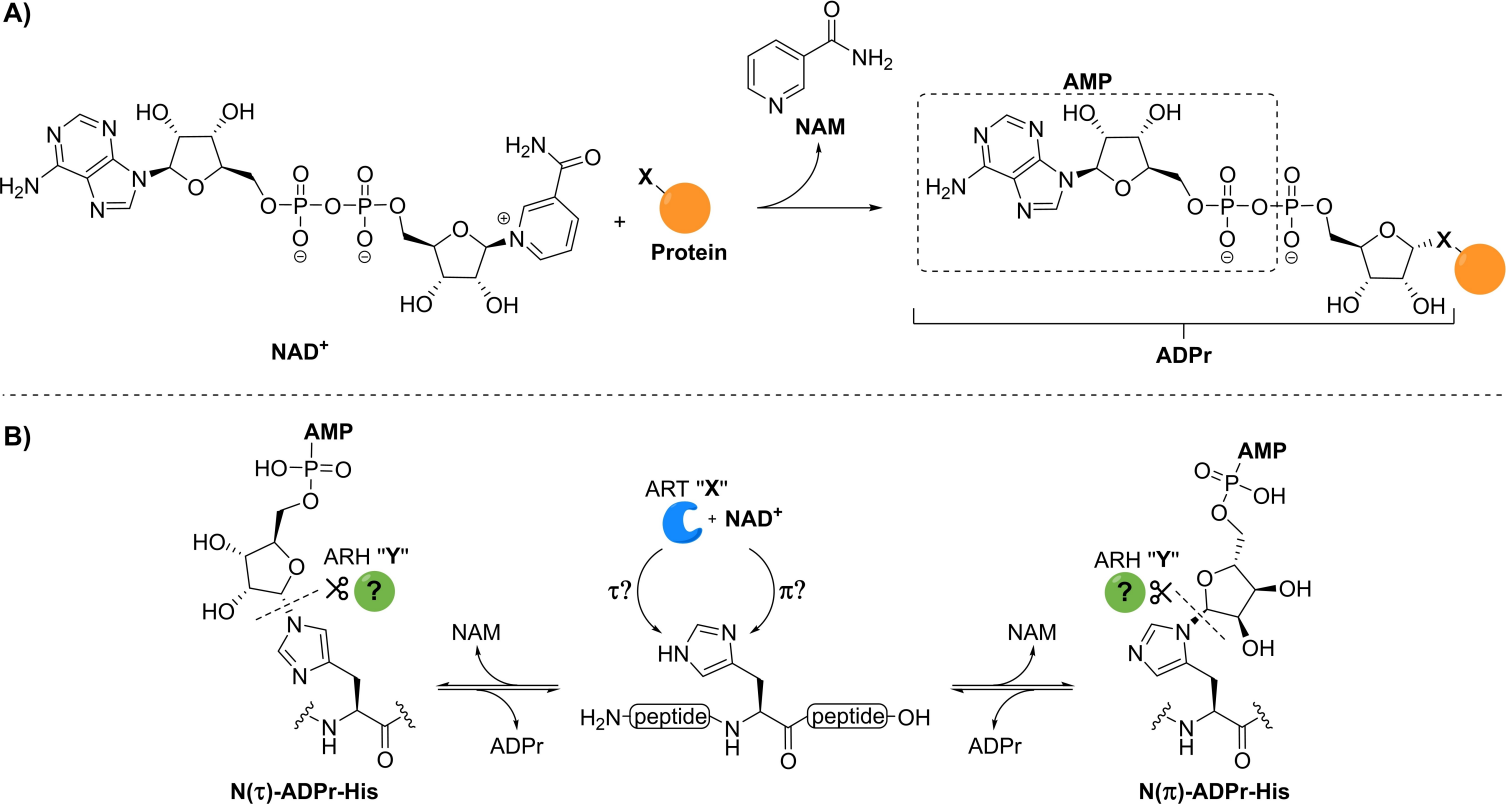
A) Schematic overview of mono‐ADP‐ribosylation where an ADPr moiety of NAD^+^ is covalently attached to a nucleophilic side chain (X=O, N or S) of the target protein in an α‐selective manner. B) Schematic overview of the ADP‐ribosylation of histidine residues including the uncertainties surrounding the nature of this specific modification. The identity of the transferase and hydrolase involved in the construction and degradation of the PTM remain unknown and a referred to as PARP “X” and ARH “Y” respectively. NAM=nicotinamide and AMP=adenosine monophosphate.

Post‐translational modifications (PTMs) comprise a vast collection of chemical alterations (e.g. methylation, phosphorylation and ubiquitination) that occur on amino acid side chains to regulate the function, localization and processing of proteins.^[1]^ These modifications are functionally associated with a wide variety of vital biological processes such as gene expression,^[2]^ cell metabolism,^[3]^ and environmental stress responses,^[4]^ and their disruption can lead to cancer, immune system dysfunction, and neurological disorders.[^3^, ^5^] Almost five decades ago, the well‐known redox co‐factor nicotinamide adenine dinucleotide (NAD^+^) was found to be responsible for a PTM, now referred to as adenosine diphosphate (ADP)‐ribosylation.[^6^, ^7^] This process is primarily mediated by (ADP‐ribosyl)‐transferases termed poly‐(ADP‐ribose) polymerases (PARPs)^[8]^ that consume NAD^+^ to construct α‐glycosidic linkages on nucleophilic amino acid residues while simultaneously expelling nicotinamide (Figure 1A). Although the majority of PARPs introduce a single ADP‐ribosyl (ADPr) molecule (mono‐ADP‐ribosylation or MARylation), a small subset of ADP‐transferases including PARP1 and PARP2 is able to extend the chain at the 2′‐OH position of the proximal ribose to generate poly‐(ADP‐ribose) (PAR) chains in a process termed poly‐ADP‐ribosylation (PARylation).^[9]^ Branching of PAR chains occurs through the occasional elongation at the 2′′‐OH position of the distal ribose, giving rise to more complex secondary structures.^[10]^ ADP‐ribosylation is a reversible PTM due to the action of distinct (ADP‐ribosyl)hydrolases (ARH). Cleavage of the polymer chain is mediated by poly(ADP‐ribosyl)glycohydrolase (PARG),[^11^, ^12^] while the removal of final protein‐linked ADPr‐moiety requires a collection of other hydrolases. Macrodomain proteins can facilitate the hydrolysis of the glycosidic ester linkages of ADP‐ribosylated aspartate and glutamate residues,[^13^, ^14^] while cleavage of ADPr from arginine and serine residues requires ARH1^[15]^ and ARH3,^[16]^ respectively.

The first ADP‐ribosylation sites to be discovered were arginine,[^17^, ^18^] cysteine^[19]^ and diphthamide,[^20^, ^21^] a uniquely modified histidine residue, which were targeted by a small number of pathogenic toxins to disrupt cellular functioning. Since then, advances in mass spectrometry (MS) have allowed for the comprehensive profiling of the “ADP‐ribosylome” to reveal the full scope of amino acid acceptors, including glutamate,[^22^, ^23^] aspartate,^[22]^ lysine,^[24]^ serine,^[25]^ and—as recently discovered—tyrosine[^26^, ^27^] and histidine.[^26^, ^28^] It has become clear that ADP‐ribosylation is a ubiquitous and highly dynamic modification that is regulated in a context‐specific manner by a vast network of transferases, hydrolases and ADP‐interacting proteins. Vital processes such as double‐strand DNA repair,^[29]^ inflammation,^[30]^ adipogenesis,^[23]^ and apoptosis^[31]^ are heavily influenced by ADP‐ribosylation in a direct manner and via cross‐talk with other PTMs[^23^, ^32^, ^33^, ^34^] and misfunctioning of the involved enzymes has been attributed, among others, to neurodegeneration,^[35]^ metabolic diseases,^[36]^ and impaired immune responses.^[30]^

Well‐defined synthetic ADP‐ribose oligomers and ADPr‐peptides have proven to be indispensable tools to unravel the mode of action of (ADP‐ribosyl) binding proteins[^37^, ^38^] and hydrolases[^39^, ^40^, ^41^] at the molecular level and they have enabled the production of anti‐ADPr antibodies^[42]^ and standards for proteomics studies.^[39]^ To this end we have previously developed synthetic methodologies that have enabled the introduction of ADP‐ribose on glutamate,^[43]^ aspartate,^[43]^ serine,^[39]^ threonine,^[41]^ and cysteine^[41]^ residues. No synthetic methods are available for the generation of ADP‐ribosylated histidine (His‐ADPr), and the exact structure of this PTM remains unknown. In line with the linkages to the other amino acid side chains and their biosyntheses,^[21]^ the ADP‐ribose moiety is most likely attached to the histidine side chain through an α‐ribosyl linkage. However, the imidazolyl moiety of histidine has two potential ADP‐ribosylation sites that are commonly referred to as the N(τ)‐ and N(π)‐positions (Figure 1B). It is unknown which of these or whether both regioisomers are of physiological importance, and as yet, no clear transferase and hydrolase candidates have been identified for the processing of His‐ADPr. Thus, to determine the structure of naturally occurring ADP‐ribosylated histidine and study the enzymes involved in their metabolism, methodology to procure both N(τ)‐ and N(π)‐α‐ADP‐ribosylated histidine peptides is desired.

**Figure 2:**
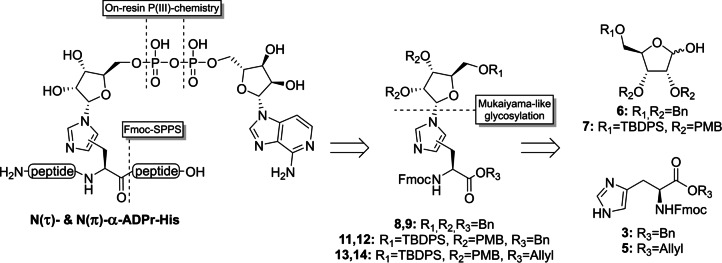
Retrosynthetic analysis of N(τ)‐ and N(π)‐ADP‐ribosylated histidine peptides. Key reactions of the novel methodology are highlighted.

Although there are numerous examples in literature for the, mainly β‐selective, introduction of substituted imidazole groups on the anomeric position of ribofuranosides,[^44^, ^45^, ^46^, ^47^] almost no procedures towards glycosylated histidine derivatives can be found.[^48^, ^49^, ^50^] Only two reports describe a condensation reaction between an α‐configured 1‐bromopyranoside with histidine or a derivative thereof. Since glycopyranosides behave differently from their furanosyl counterparts in terms of reactivity and stereoselectivity, a new and effective methodology is required to construct the glycosidic linkage between histidine and ADP‐ribose. Here, we describe the preparation of Fmoc‐N^im^‐ribosylhistidine building blocks **8**, **9 & 11**–**14**, compatible with solid‐phase peptide synthesis (SPPS), starting from suitably protected ribofuranosides **6** and **7** and histidine derivatives **3** and **5** using a base‐assisted Mukaiyama‐like glycosylation approach (Figure 2).[^51^, ^52^] The first methodology towards α‐configured N(τ)‐ and N(π)‐mono‐ADP‐ribosylated histidine‐containing peptides has subsequently been developed by implementing the modified histidine building blocks in a peptide sequence of choice followed by on‐resin construction of the ADPr moiety using phosphoramidite chemistry. In addition, the chemical stability of ADPr‐histidine peptides under various conditions as well as their sensitivity to enzymatic digestion by (ADP‐ribosyl)hydrolases, were evaluated.

## Results and Discussion

In order to synthesize ADPr‐His‐peptides using the general Fmoc‐based solid‐phase approach^[41]^ the N^im^‐ribosylated histidine building blocks had to be made accessible first. Toward this end, histidine benzyl ester **3** ^[53]^ and allyl ester **5** ^[54]^ were prepared from the commercially available precursor Fmoc‐His(Trt)‐OH in 2 steps (Scheme S1). Although the use of ribofuranosyl imidate donors has enabled the highly α‐selective glycosylation of a variety of amino acid functionalities,[^38^, ^41^] no product formation was detected in the attempted ribosylation reaction of acceptor **3** or **5** and a ribosyl imidate donor under the aegis of catalytic or stochiometric amounts of TMSOTf. Silylation of the imidazole nitrogen prior to the glycosylation proved to be of no avail. We decided, therefore, to switch to a glycosylation methodology that can operate under basic conditions and were drawn to the α‐selective ribosylation reaction, introduced by Mukaiyama and co‐workers,^[51]^ in which anomeric alcohol of a suitably protected ribofuranoside reacts with a diphosphonium triflate salt in the presence of a non‐nucleophilic base to produce a reactive phosphonium ribosyl intermediate. The acceptor and additional base are then added to the activated donor to yield the desired ribosylated product. According to optimized conditions reported in the literature,^[52]^ ribose derivative **6** was added with diisopropylethylamine (DIPEA) to an in situ prepared bis‐phosphonium salt prepared from tributylphosphine and triflic anhydride (Table 1). The resulting phosphonium riboside species, which could be conveniently monitored by ^31^P NMR (94 and 98 ppm), was added to a limiting amount of Fmoc‐histidine benzyl ester **3** (0.6 equiv, entry 1). It quickly became apparent that both imidazole nitrogen atoms were reactive under the given conditions since the complex mixture of isolated ribosylated histidine derivatives showed a substantial amount of doubly substituted product **10**. Formation of side product **10** could be successfully reduced by the slow addition of the activated donor solution to a two‐fold excess of the acceptor over 30 min using a syringe pump (entry 2). Notably, a total of three distinct mono‐substituted products were formed (Table1). Heteronuclear multiple bond correlation (HMBC) measurements demonstrated that a single anomer of N(π)‐ribosylated histidine **9** was obtained alongside an inseparable mixture of α‐ and β‐anomers of N(τ)‐regioisomer **8** (Table 1). In view of the late‐stage on‐resin construction of the pyrophosphate functionality, orthogonally protected ribose **7** was subjected to the optimized reaction conditions (entry 3). The bulky silyl protecting group on the ribose 5‐OH position did not have a significant impact on the selectivity of this reaction as regioisomers **11** and **12** were acquired in a similar ratio albeit in slightly higher yields. Allyl ester **5** was also found to be compatible with the Mukaiyama‐type glycosylation conditions, although for this acceptor, the formation of the N(π)‐substituted product **14** prevailed over its N(τ)‐counterpart **13** (entry 4). Treatment of allyl ester **5** with the silylating agent BSTFA prior to addition of the activated donor (entry 5) enhanced the reactivity of the imidazolyl sidechain somewhat to provide the mixture of the three mono‐ribosylated products in an improved overall yield. However, this was accompanied by an increase in di‐ribosylated side products **10**, which affected the yield of the N(τ)‐regioisomers **13**, because of separation complications. Comparable difficulties with overglycosylation were encountered when the reaction was performed on a larger scale (entry 6). Nevertheless, ribosylated histidine analogues **8**, **9** and **11**–**14** could be consistently isolated in satisfactory yields and provided sufficient amounts to finalize the synthesis of the building blocks for Fmoc‐SPPS.

**Table 1:**
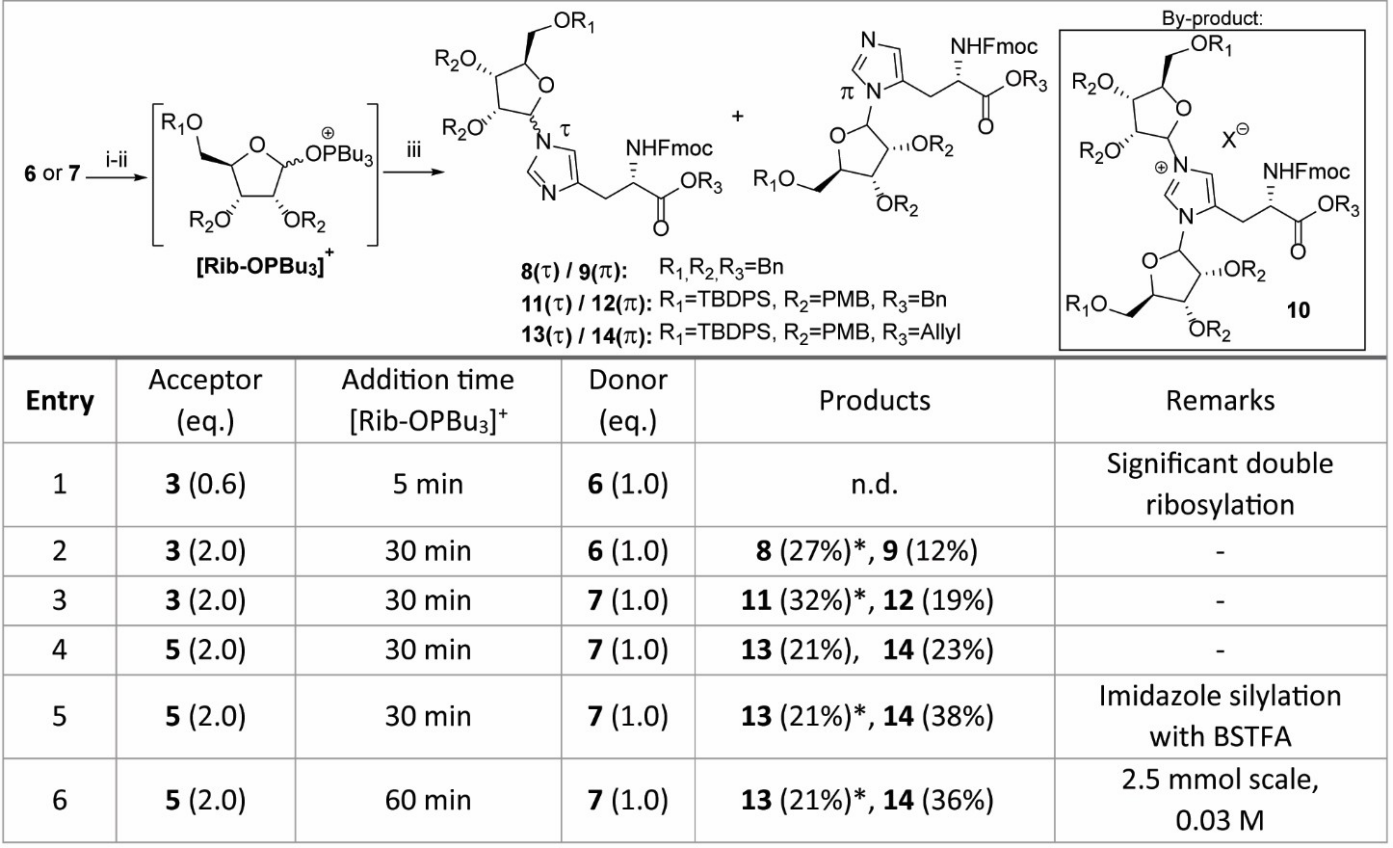
Ribosylation of Fmoc‐protected histidine analogues using diphosphonium salts. Reaction were performed at 0.5 mmol scale (limiting reagent) with a final concentration of 0.013 M. Reagents and conditions: i) Bu_3_PO, Tf_2_O, DCE, 0 °C,1 h. ii) **6** or **7**, DIPEA, DCE, 0 °C, 1 h. iii) **3** or **5**, DIPEA, DCE, 0 °C to rt, 16 h.

* double ribosylated product **10** was detected by LC–MS, quantified by ^1^H NMR and the yields were corrected accordingly.

**Scheme 1:**
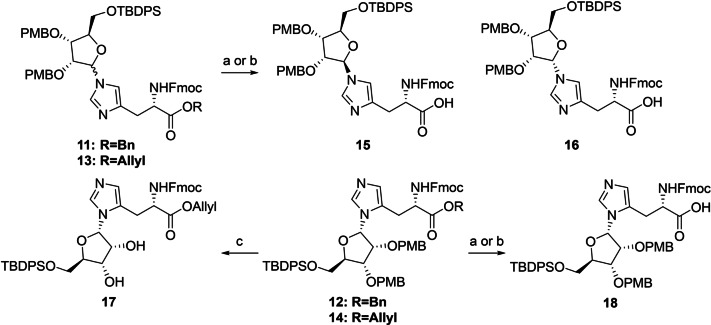
Protecting group manipulations of ribosylated histidine analogues. Reagents and conditions: a) LiOH, THF/H_2_O (3 : 1), 0 °C, 1.5 h (46 % from **11**, 47 % from **12**). b) Pd(PPh_3_)_4_, DCM, rt, 1 h (78 % from **13**, 89 % from **14**). c) HCl, HFIP, 0 °C, 10 min (36 %).

Next, the benzyl ester of inseparable anomeric mixture **11** was hydrolyzed, while keeping the base‐labile Fmoc group intact, using a 1.5 fold excess of lithium hydroxide at 0 °C (Scheme 1). At this stage, the two formed products showed sufficient differences in retention time to allow for separation by careful silica gel column chromatography. Although MS and NMR analysis confirmed the isolated compounds to be the N(τ)‐ribosylated histidines **15** and **16**, their anomeric configuration could not be reliably established. In parallel, deallylation of epimeric mixture **13** could be efficiently mediated by palladium(0) catalysis in the presence of dimethyl barbituric acid (DMBA) as allyl scavenger to provide the N(τ)‐anomers **15** and **16** in significantly higher yields than the deprotection of the corresponding benzyl ester described above. Similarly, saponification of N(π)‐ribosylated benzyl ester **12** led to carboxylic acid **18** in moderate yield (47 %), while the Pd(0) catalyzed deallylation of **14** provided the same product in 89 % yield. Clearly the allyl ester is the preferred protection group strategy for the carboxylic acid of histidine in this synthetic endeavor.

**Figure 3:**
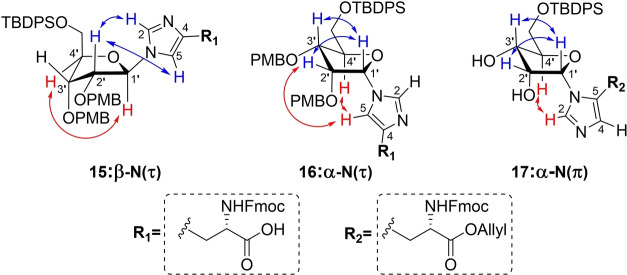
Schematic representation of the three ribosylated histidine analogues **15 16** and **17** depicted in their presumed ^3^E or E_3_ envelope conformation, with the most relevant observed proton‐proton interactions observed in NOESY measurements highlighted in red and blue. The H1′→H2′ interaction in structure **15** has been omitted for clarity.

Due to marginal differences in both the chemical shift and coupling constants of the anomeric proton and carbon atoms, additional NMR‐spectroscopic analyses were required to elucidate the configuration of the three histidine derivatives **15**, **16** and **18**. The use of heteronuclear single quantum coherence (HSQC) HECADE measurements^[55]^ was explored, which allows for the quantitative measurement of proton‐carbon *J‐*couplings (*J_CH_*). When the H1 and H2 of furanosides are *cis*‐oriented the *J*_*H1‐C2*_ is >0 Hertz, while *trans*‐related protons show coupling constants *J*_*H1‐C2*_ <0 Hertz.^[56]^ Unfortunately, the observed coupling constants were all equal to zero and thus did not provide any clues regarding the relative orientation of H1′ and H2′. Therefore, we measured through space interactions between the protons using NOESY measurements (Figure 3). In the spectra of both N(τ)‐conjugated anomers **15** and **16**, a strong interaction with the neighboring H2′ could be observed (Figure S15 and S16, respectively). However, a signal between H1′ and H4′ in compound **15** indicated the imidazole functionality to have the β‐configuration. This hypothesis was further substantiated by significant interactions between H1′ and H3′, and H5 and H4′ in **16**, pointing to the α‐configuration of this isomer. N(π)‐ribosylated carboxylic acid **18** and its allyl ester precursor **14** both displayed remarkably weak NOESY signals, so both PMB ethers were removed using a catalytic amount of HCl in HFIP^[57]^ to provide diol **17**, which revealed clear NOESY signals (Figure S17). Similar to α‐anomer **16**, the anomeric proton (H1′) of **17** interacted with H2′ as well as H3′ while a coupling with H4′ was lacking. In addition, an interaction of the imidazole H2 proton with H4′ supported the α‐configuration of this histidine isomer.

**Figure 4:**
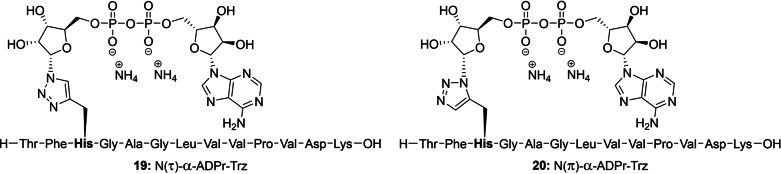
Chemical structures of the α‐configured 1,4‐ and 1,5‐disubstituted 1,2,3‐triazole‐based isosteres, here referred to as ADPr‐Trz that mimic their N(τ)‐ and N(π)‐ADP ribosylated histidine counterparts, respectively.

**Scheme 2:**
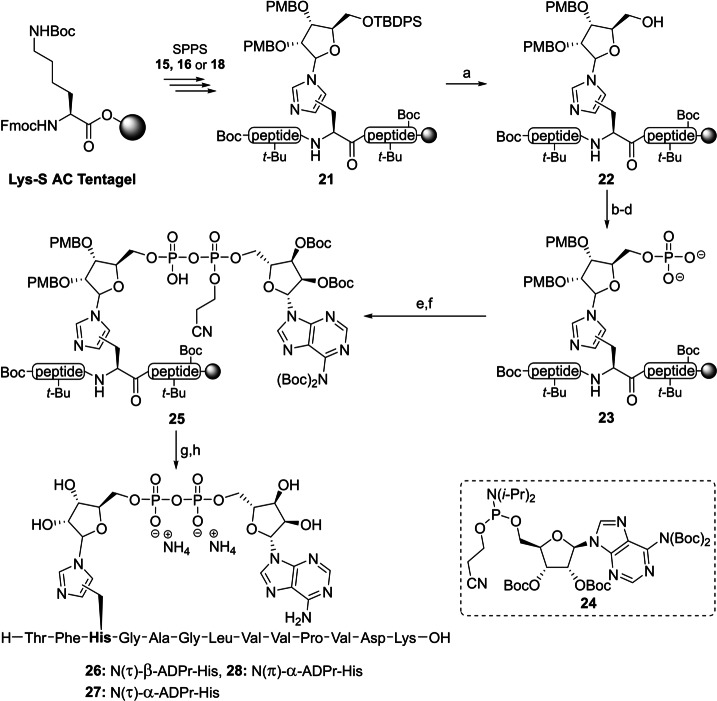
Incorporation of ribosylated histidine building blocks **15, 16** and **18** and in a peptide fragment originating from HPF1 a) HF‐pyridine, pyridine, rt, 2x 45 min. b) (FmO)_2_N(*i‐*Pr)_2_, ETT, MeCN, rt, 30 min. c) CSO, MeCN, rt, 30 min. d) DBU, DMF, rt, 2x 15 min. e) **24**, ETT, MeCN, rt, 30 min. f) CSO, MeCN, rt, 30 min. g) DBU, DMF, rt, 2x 10 min. h) TFA, DCM, rt, 1 h (28 % for **26**, 35 % for **27** and 28 % for **28** over 8 steps).

With the configuration of all three ribosylated Fmoc‐histidine derivatives **15**, **16** and **18** established, their incorporation in an oligopeptide using SPPS was investigated. β‐Configured analogue **15** was included as it can function as a suitable negative control in later biochemical experiments. A 13 amino acid‐long sequence originating from histone PARylation factor 1 (HPF1) presents an attractive target as the histidine residue 223 has recently been identified as an ADP‐ribosylation site in proteomic studies.^[58]^ Furthermore, we have observed the enzymatic hydrolysis of triazolyl‐based ADP‐ribosylated HPF1 peptides **19** and **20** (generated by click reactions between an 1′′‐azidoribose with a propargyl glycine residue and representing a close mimic of ADPr‐His, referred to as ADPr‐Trz, Figure 4) by (ADP‐ribosyl)hydrolase 3 (ARH3).^[**40**,59]^ Thus, the fully protected ribosylated peptides (general structure **21**) were successfully synthesized on a Tentagel resin, pre‐loaded with lysine, by implementing the respective building blocks (**15**, **16** or **18**) through standard Fmoc‐based SPPS (Scheme 2). The TBDPS group in each of the ribosylated peptides was efficiently cleaved using HF‐pyridine to liberate the primary alcohol of the immobilized ribosylpeptides (general structure **22**), after which the resin was thoroughly washed with DCM, Et_2_O and finally, anhydrous MeCN to remove any leftover base or water before the following steps. Treatment of the resin‐bound ribosyl peptides **22** with the bis‐fluoronylmethyl (Fm) protected phosphoramidite under the aegis of ethylthiotetrazole (ETT) as activator resulted in the phosphotriester intermediates that were immediately converted to the corresponding phosphates using (1S)‐(+)‐(10‐Camphorsulfonyl)oxaziridine (CSO). Next, the Fm‐groups of the phosphates were removed by treatment with dry 1,8‐Diazabicyclo(5.4.0)undec‐7‐ene (DBU) to give phosphoribosyl peptides (general structure **23**) ready for the condensation with protected adenosine phosphoramidite **24** ^[60]^ through the P(III)‐P(V) coupling method we developed for the construction of pyrophosphates.^[61]^ The coupling was followed directly by oxidation using CSO to give immobilized protected ADPr‐His‐peptides (general structure **25**). Elimination of the cyanoethyl group was performed with DBU followed by the global deprotection and simultaneous cleavage of the target peptides **26**–**28** from the resin using 50 % TFA in DCM. The target ADPr‐His‐peptides were then purified using HPLC chromatography. The use of an ammonium acetate buffered mobile phase conveniently provided the ADP‐ribosylated peptides **26**–**28** as stable ammonium salts but in an inadequate purity. Fortunately, the resolution of the peaks during the HPLC purification could be sufficiently enhanced using an eluent buffered with acetic acid, allowing for the isolation of all three oligopeptides in a high purity. Neutralization of the collected HPLC fractions with ammonium hydroxide before lyophilization smoothly converted the pyrophosphates into their respective ammonium salts **26**–**28**.

**Figure 5:**
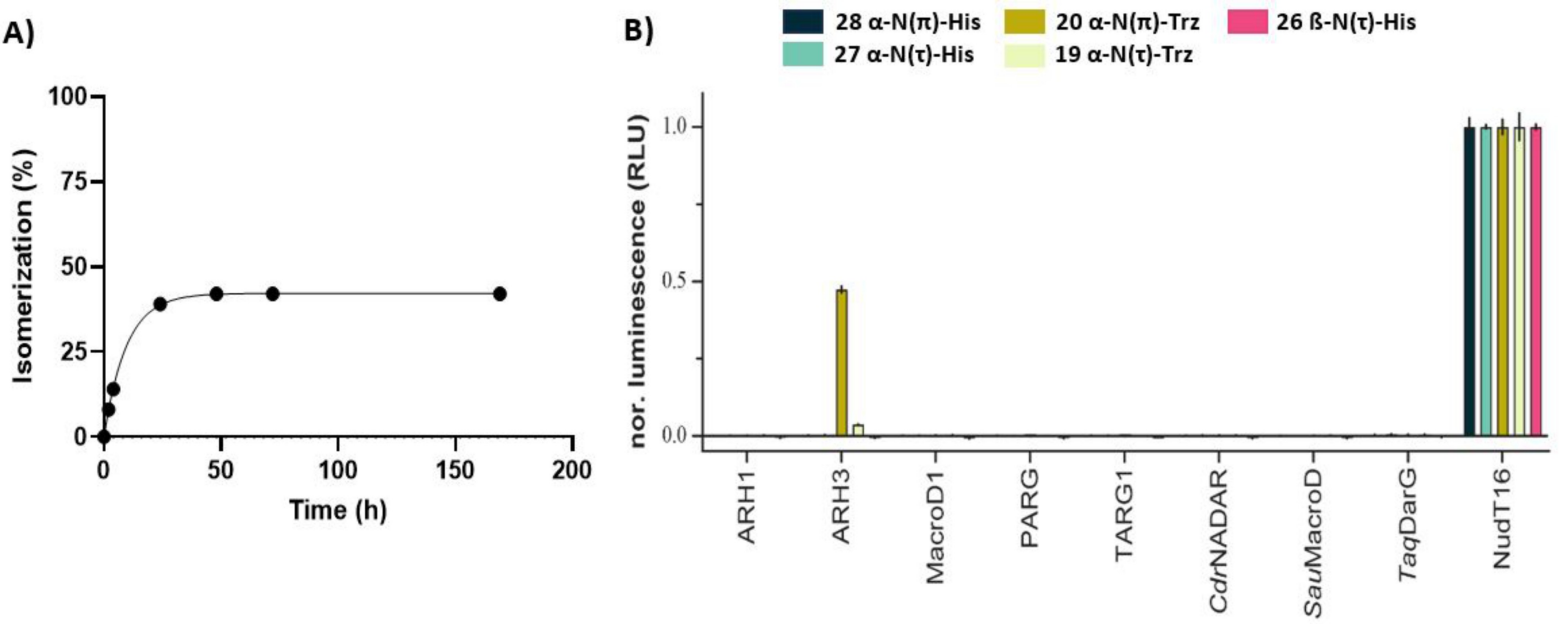
A) Chemical stability of peptide **28** under basic conditions (NaOH, 0.1 M). Samples were extracted at different time points (2, 4, 24, 48, 72 and 169 h) and quenched with TFA prior to LC–MS injection. Peptide degradation was quantified by analyzing the UV‐trace (260 nm) using Xcalibur software. Including an exponential one‐phase decay trendline (Y=42.2*e^(−0.1024*X)+57.8, R^2^=0.999). B) Enzymatic hydrolysis of interglycosidic linkages in ADP‐ribosylated histidine peptides **26**–**28** and 1,2,3‐triazole‐based isosteres **19** and **20**. Enzymatic turnover of the various peptides was assessed by monitoring AMP release directly (NudT16) or converting released ADPr via NudT5 to AMP. AMP was measured using the AMP‐Glo assay (Promega). Samples are background corrected and normalized to NudT16 activity The data represents mean values±SD measured in triplicates. Abbreviations: *Cdr*NADAR, NADAR from *Clostridium drakei*; *Sau*MacroD, zinc‐containing MacroD from *Staphylococcus aureus*; TaqDarG, DarG from *Thermus aquaticus*.

Having prepared ADPr‐His peptides **26**–**28**, we tested their stability under basic and acidic conditions in an LC–MS‐based assay to evaluate their sensitivity for future synthetic and biochemical studies. A selected set of chemically diverse conditions (aqueous acid, aqueous base and a neutral nucleophile) was probed because these are commonly applied in proteomic studies to identify ADP‐ribosylation sites^[22]^ in proteins or during sample preparation.[^14^, ^28^] Aqueous solutions of the ADPr peptides with a final concentration of 0.1 M TFA, 0.1 M NaOH or 0.5 M NH_2_OH were prepared and shaken for 24 h at room temperature. No detectable degradation was observed for any of the peptides under the TFA and NH_2_OH conditions. In contrast, the NaOH treatment led to some conversion of the N(τ)‐ribosylated histidines **26** and **27** into products with an identical mass (approximately 7 and 11 %, respectively), indicating a slow epimerization of the modified histidine residue in N(τ)‐ribosyl peptides. Isomerization of ADPr‐peptide **28** was significantly faster, and this substrate was therefore monitored in a follow‐up time‐course experiment (Figure 5A). After 24 h, no more isomerization is observed, leading to a mixture in which approximately 60 % of the ADPr‐peptide remains in its original form. No cleavage of the N‐glycosidic bond of the distal ribose has been observed in any of the samples, which is in correspondence to the stability described for glucopyranosylimidazoles under both acidic and basic conditions.^[62]^ The stability of ADPr‐His to aqueous hydroxylamine and acid and the sensitivity of it to base‐catalyzed epimerization effectively parallels the behavior of the triazole‐based isosteres of ADPr‐His reported by us previously.^[59]^ These chemical similarities support the idea that the latter is indeed a suitable substitute for His‐ADPr at least in some respects. It is noteworthy that even after a week of the alkaline treatment, no degradation of the pyrophosphate linkage was observed. Combined with earlier findings,^[39]^ this suggests that the pyrophosphate in the mono‐ADPr‐conjugates is stabilized by the peptide backbone and only becomes susceptible to nucleophilic degradation once it has been eliminated from the amino acid residue. With the chemical stability of conjugates **26**–**28** established, we next probed whether any of the so‐far identified human (ADP‐ribosyl)hydrolases has the ability to catalyze the turnover of the histidine modifications (Figure 5B).

To this end, we incubated peptides **26**–**28**, as well as the close ADP‐ribosyl histidine mimics **19** and **20**,^[59]^ with different (ADP‐ribosyl)hydrolases in combination with Nudix hydrolase (NudT) 5. The former hydrolases have been shown to cleave mono‐ADP‐ribose moieties from aspartic acid and glutamic acid (MacroD1, TARG1 and *Sau*MacroD), arginine (ARH1), serine (ARH3),^[63]^ cysteine (*Sau*MacroD),^[41]^ and ADP‐ribosylated DNA modified at the N3 position of a thymidine (TARG1 and *Taq*DarG)[^64^, ^65^] or N2 position of a guanosine base (*Cdr*NADAR),^[66]^ while NudT5 transforms the liberated ADPr into adenosine monophosphate (AMP) that allows for the quantification of hydrolase activity using the commercial AMP‐Glo^TM^ assay (Promega).^[39]^ Luminescent signals are normalized against NudT16, an enzyme capable of hydrolyzing the pyrophosphate linkage of both free and peptide‐bound ADPr.^[67]^ To our surprise, none of the tested hydrolases are capable of cleaving the ADPr‐modification on any of the three histidine peptides, while ARH3 was able to turn over ADP‐ribosyl triazolides in peptides **19** and **20**.[^40^, ^59^] The sensitivity of the ADPr‐triazoles to ARH3 in vitro probably results from a combination of the relative promiscuity in hydrolase activity of ARH3 and the good leaving group capacity of the triazolide anion (pKa (1,2,3‐triazole)=9.4 versus pKa (imidazole)=14.5). The high chemical and enzymatic stability of ADP‐ribosylated histidine, on the one hand, may suggest irreversibility of this PTM and, on the other hand, invites the search for the yet unidentified enzyme(s) capable of cleaving the ADPr‐histidine linkage. The tools generated here will be instrumental in this quest. At the same time, at present no definite conclusions can be drawn about the structure of ADP‐ribosylated histidine residues regarding the modification site on the histidine imidazole ring.

## Conclusion

A methodology for solid‐phase synthesis of α‐configured ADP‐ribosylated histidine peptides has been developed for the first time. The synthetic well‐defined ADPr‐His‐peptides will assist in determining the exact structure of this modification. They may enable the discovery of hydrolases that can reverse ADP‐ribosylation of histidine in living organisms and proteins containing macrodomains capable of binding ADPr‐His specifically. The synthesis of the desired ribosylated building blocks α‐N(τ) **16** and α‐N(π) **18** was realized through a modified Mukaiyama glycosylation reaction using a suitable protected, in situ generated phosphonium ribofuranoside donor. The exact structure of three key ribosyl histidine intermediates was elucidated using HMBC and NOESY measurements. The desired ADPr‐peptides **26**–**28** were generated using standard Fmoc‐based SPPS combined with effective P(III)–P(V) pyrophosphate chemistry. The histidine ADPr modification proved to be rather stable under both chemical (acidic, nucleophilic, and basic) conditions as well as enzymatic degradation conditions. Surprisingly, none of the known human (ADP‐ribosyl)hydrolases can cleave ADPr‐His. These findings are all the more striking, considering the extensive substrate spectrum of ARH3 (including α‐NAD^+^,^[68]^ poly‐ADPr,^[69]^ O‐acetyl‐ADPr,^[70]^ ADPr‐Ser,^[39]^ ADPr‐5′P DNA,^[71]^ and ADPr‐triazolyl conjugates),^[59]^ and suggest the existence of a yet unidentified enzyme with (ADP‐ribosyl)hydrolase activity towards histidine. Our newly synthesized tools will be of great value in the discovery of these enzymes and in uncovering new macrodomains capable of reading the ADPr code.

## Conflict of interest

The authors declare no conflict of interest.

1

## Supporting information

As a service to our authors and readers, this journal provides supporting information supplied by the authors. Such materials are peer reviewed and may be re‐organized for online delivery, but are not copy‐edited or typeset. Technical support issues arising from supporting information (other than missing files) should be addressed to the authors.

Supporting Information

## Data Availability

The Supporting Information is available free of charge at: Synthetic procedures for histidine acceptors **3** and **5** and ribosylated building blocks **8**‐**9** and **11‐18**; protocols for solid‐phase peptide synthesis, on‐resin phosphorylation, pyrophosphate construction and global deprotection as well as for plasmid expression, protein purification, and (ADP‐ribosyl)hydrolase activity screening; general experimental procedures; and copies of (^1^H, ^13^C, HMBC and NOESY) NMR and LC–MS spectra.
