# Supplementary material for: Solid‐Phase Synthesis and Biological Evaluation of Peptides ADP‐Ribosylated at Histidine
Source: Angew Chem Int Ed Engl. 2023 Nov 14;63(4):e202313317. doi: 10.1002/anie.202313317 (PMC10952301; doi:10.1002/anie.202313317)
Supplement: Supplementary file 1 — Supporting Information [file ANIE-63-0-s001.pdf]

Angewandte Chemie
Eine Zeitschrift der Gesellschaft Deutscher Chemiker

Supporting Information

Solid-Phase Synthesis and Biological Evaluation of Peptides ADP-Ribosylated at Histidine

*H. Minnee, J. G. M. Rack, G. A. van der Marel, H. S. Overkleeft, J. D. C. Codée, I. Ahel, D. V. Filippov**

Supporting Information

Table of Contents

Experimental Procedures 3
Plasmid construction 3
Protein expression and purification 3
Hydrolase activity assay 3
Chemical Synthesis 3
Solid-phase peptide synthesis 4
On-resin phosphorylation, pyrophosphate construction and final deprotection 4
Chemical stability assay 13
References 18
NMR spectra 19
Figure S5: ^{1}HNMR & ^{13}CNMR of compound **2** 19
Figure S6: ^{1}HNMR & ^{13}CNMR of compound **3** 20
Figure S7: ^{1}HNMR & ^{13}CNMR of compound **4** 21
Figure S8: ^{1}HNMR & ^{13}CNMR of compound **5** 22
Figure S9: ^{1}HNMR & ^{13}CNMR of compound **8** 23
Figure S10: ^{1}HNMR & ^{13}CNMR of compound **9** 24
Figure S11: ^{1}HNMR & ^{13}CNMR of compound **11** 25
Figure S12: ^{1}HNMR & ^{13}CNMR of compound **12** 26

Figure S13: ^{1}HNMR, ^{13}CNMR & HMBC of compound **13** 28
Figure S14: ^{1}HNMR, ^{13}CNMR & HMBC of compound **14** 30
Figure S15: ^{1}HNMR, ^{13}CNMR & NOESY of compound **15** 32
Figure S16: ^{1}HNMR, ^{13}CNMR & NOESY of compound **16** 34
Figure S17: ^{1}HNMR, ^{13}CNMR & NOESY of compound **17** 36
Figure S18: ^{1}HNMR & ^{13}CNMR of compound **18** 37
Figure S19: ^{1}HNMR, ^{31}PNMR & LCMS of compound **26** 39
Figure S20: ^{1}HNMR, ^{31}PNMR & LCMS of compound **27** 41
Figure S21: ^{1}HNMR, ^{31}PNMR & LCMS of compound **28** 43

Experimental Procedures

Plasmid construction

Construction of the expression plasmids for ARH1, ARH3, MacroD1, PARG, TARG1, *Sau*MacroD and *Taq*DarG were described earlier.[1–4] Amino acid sequence from *Clostridium drakei* NADAR (WP_032077447) was converted into coding sequence, optimized for expression in E. coli and string DNA fragment synthesized using the GeneArt service (Tjhermo Fisher scientific). The fragment containing PagI and BamHI restriction sites was subcloned in pET9H_3 digested with NcoI and BamHI for expression.[2]

Protein expression and purification

Recombinant proteins were expressed in Rosetta (DE3) cells grown in LB medium supplemented with 2 mM $MgSO_4$ and appropriate antibiotics at 37 °C to OD_{600} 0.6. Expression was induced with 0.4 mM IPTG and 5 µM zinc acetate in case of *Sau*MacroD. Cells were grown at 30 °C and harvested 4 hr post-induction by centrifugation (4500 x*g* for 15 min at 4 °C). Cell pellets were resuspended in lysis buffer (50 mM TrisHCl [pH 8], 500 mM NaCl, 10 mM imidazole) and stored at -20 °C until use. Recombinant His-tagged proteins were purified by Ni^{2+}-NTA chromatography (Serva Electrophoresis GmbH) according to the manufacturer's protocol using the following buffers: all buffer contained 50 mM TrisHCl [pH 8] and 500 mM NaCl; additionally, the lysis/binding buffer contained 10 mM imidazole, the washing buffer contained 30 mM imidazole, and the elution buffer contained 500 mM imidazole. Eluted proteins were dialysed against storage buffer (50 mM TrisHCl [pH 8], 200 mM NaCl, 1 mM DTT, 5% (v/v) glycerol) overnight at 4 °C and stored at -80 °C until use.

Hydrolase activity assay

The peptide demodification assay was described earlier.[5] Briefly, peptide concentration for the assay were estimated using absorbance at λ_{260nm} using the molar extinction coefficient of ADP-ribose (13,400 M^{-1} cm^{-1}). 10 µM indicated peptide were demodified by incubation with 1 µM hydrolase for 60 min at 30 °C in assay buffer (50 mM TrisHCl [pH 8], 200 mM NaCl, 10 mM $MgCl_2$, 1 mM DTT and 0.2 µM human NudT5).[6] Reactions were stopped and analyzed by performing the AMP-Glo™ assay (Promega) according to the manufacturer's protocol. Luminescence was recorded on a SpectraMax M5 plate reader (Molecular Devices) and data analyzed with GraphPad Prism 10.0.2. All reaction were background corrected using a control reaction without hydrolase, but containing NudT5.

Chemical Synthesis

5-TBDPS-2,3-bis-*O*-PMB-D-ribofuranose[7] and 2,3,5-tri-*O*-benzyl-D-ribofuranose[8] were synthesized, over 3 and 4 steps respectively, from D-ribofuranose according to previously reported procedures. All chemicals were used as received unless stated otherwise. Dowex 50WX8 hydrogen form (100-200 mesh) was purchased at Sigma Aldrich and washed with H_2SO_4 (5 M, 3x) and MeOH (3x) prior to use. Molecular sieves were flamedried (3x) in vacuo before use. Solvents were dried over activated 4Å molecular sieves for 24 h except for MeCN and MeOH which were dried over 3Å molecular sieves. A solution of HCl (0.2 M in HFIP) was freshly prepared prior to the reaction by dissolving HCl (37%, 0.1 ml) to HFIP (5.9 ml). Reactions were performed under N_2 atmosphere unless stated otherwise. A Julabo FT902 cryostat was used for low temperature glycosylation reactions. Reaction mixtures were concentrated under reduced pressure using rotary evaporators at 40-45 ˚C unless state otherwise. Reactions were monitored by thin layer chromatography (TLC) analysis using silica gel 60 F254 coated aluminum sheets from Merck. TLC plates

were visualized with ultraviolet light (254 nm) or sprayed with H_2SO_4 (20% v/v in MeOH), potassium permanganate (1 g $KMnO_4$, 5 g K_2CO_3, in 200 ml H_2O) or ceric ammonium molybdate (1 g $Ce(NH_4)_4(SO_4)_4 \bullet 2H_2O$, 2.5 g $(NH_4)_6Mo_7O_{24} \bullet 4H_2O$, 10 ml H_2SO_4 in 90 ml H_2O). Infrared (IR) values are reported in cm^{-1}. ^1H NMR, ^{13}C NMR and ^{31}P NMR spectra were recorded on Bruker AV-300 (300 mHz), AV-400 (400 mHz) or AV-500 (500 mHz) spectrometer. ^{13}C NMR spectra are acquired via the attached proton test (APT) experiment and are presented with even signals (C_q and CH_2) pointing upwards and odd signals (CH and CH_3) pointing downwards. The chemical shifts are noted as δ-values in parts per million (ppm) relative to the tetramethylsilane signal (δ = 0 ppm) or solvent signal of D_2O (δ = 4.79 ppm) for ^1H NMR and relative to the solvent signal of $CDCl_3$ (δ = 77.16 ppm) for ^{13}C NMR. Phosphorylation reactions were monitored with ^{31}P NMR using an acetone-D_6 insert for a locking signal and the resulting spectra were indirectly calibrated with H_3PO_4. HRMS samples were prepared in either MeOH, MeCN or milliQ grade H_2O with an approximate concentration of 1 mM and measured on a Thermo Scientific LTQ Orbitrap XL.

Solid-phase peptide synthesis

Fmoc-Asp(t-Bu)-OH, Fmoc-Val-OH, Fmoc-Pro-OH, Fmoc-Leu-OH, Fmoc-Gly-OH, Fmoc-Ala-OH and Fmoc-Phe-OH were all obtained from Merck Novabiochem. Boc-Thr(tBu)-OH was acquired from BLD pharmatech GmbH. Lysine(Boc) was purchased pre-loaded on tentagel® S AC resin from RAPP Polymere GmbH.

The Fmoc-N-Ala-Gly-Leu-Val-Val-Pro-Val-Asp-Lys-S AC linked Tentagel® sequence was prepared using a Liberty Blue peptide synthesizer via 9-fluorenylmethoxycarbonyl (Fmoc) based solid phase peptide chemistry at a 250 µmol scale. A 4-fold excess of the amino acids relative to the resin loaded amino acid was added in each prolongation step. A total of 4 equivalents of the additives Diisopropylcarbodiimide (DIC) and OxymaPure were added simultaneously. The coupling was established in the microwave reaction chamber at 90 °C for 2.5 minutes. After each coupling the peptide was subjected three consecutive times to a 20 v/v% piperidine solution in DMF at 90 °C for 1 minute to remove the Fmoc protection group.

The Fmoc-N-Ala-Gly-Leu-Val-Val-Pro-Val-Asp-Lys-Tentagel® sequence was completed at a 50 µmol scale by hand in a fritted syringe. After each step the resin was rinsed with the corresponding solvent (3x 3 ml) unless stated otherwise. Fmoc proctecting groups were removed by treatment with piperidine twice (10v/v% in DMF, 3 ml) for 3 and 7 min. Ribosylated amino acids **14**, **15** or **18** (0.1 mmol, 2 eq.) were coupled overnight in the presence DIPEA (40 µl, 4.5 eq.) and HCTU (0.1 mmol, 2 eq.) in DMF (3 ml). Fmoc-Phe-OH and Boc-Thr(t-Bu)-OH (0.25 mmol, 5 eq) were coupled for 45 min with HCTU (0.25 mmol, 5 eq) and DIPEA (90 µl, 10 eq.) in DMF (3 ml).

On-resin phosphorylation, pyrophosphate construction and final deprotection

TBDPS deprotection was achieved by treating the resin (50 µmol) with HF-pyridine (70 wt%, 1 ml) in pyridine (3 ml) twice for 45 min. The resin was washed with DMF (3x 3ml), DCM (3x 5 ml), Et_2O (3x 5 ml) and anhydrous MeCN (3x 3 ml) and flushed with nitrogen to minimize traces of water. Subsequently, the desilylated intermediate was treated with $(FmO)_2PN(i\text{-}Pr)_2$ (0.25 mmol, 5 eq.) and ETT (0.25 mmol, 5 eq.) in anhydrous MeCN (3 ml) for 30 min. CSO (1 mmol, 20 eq.) in anhydrous MeCN (2 ml) was added and the resin was shaken again for 30 min. Then the newly introduced phosphate was deprotected with DBU (10v/v% in DMF, 2 ml) twice for 15 min and thoroughly washed with DCM (3x 5 ml), Et_2O (3x 5 ml) and anhydrous MeCN (3x 3 ml) before treating with **24**[9] (0.2 mmol, 4 eq.) in the presence of ETT (0.4 mmol, 8

eq.) in anhydrous MeCN (3 ml) for 30 min. The P(III)-P(V) intermediate was oxidized again with CSO (1 mmol, 20 eq.) in anhydrous MeCN (2 ml) for 30 min. The resin was subsequently shaken with DBU (10v/v% in anhydrous DMF, 2 ml) twice for 10 min each to remove the cyanoethyl group. The modified oligopeptide was cleaved from the resin using TFA/TIS/DCM (50:50:2.5, 4 ml) for 1 h. The solution was poured into a Falcon® tube containing ice cold Et_2O (45 ml) and the resulting suspension was centrifuged (5 min, 3000 RCF) using an Eppendorf centrifuge 5702 followed by removal of the supernatant. The crude residues were neutralized with NH_4OH (28wt% in H_2O), lyophilized and subjected to preparative RP-HPLC (AcOH buffered system). Product fractions were collected and neutralized with NH_4OH (28wt% in H_2O) prior to lyophilisation to yield the desired peptide conjugate as ammonium salt.

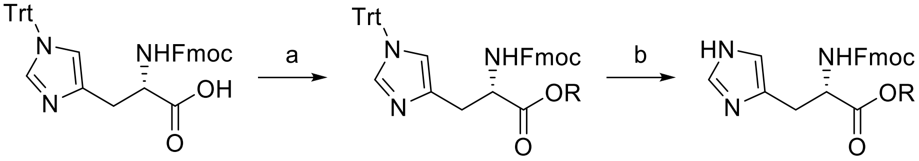

1 **2:** R=Bn **4:** R=Allyl **3:** R=Bn **5:** R=Allyl

Scheme S1. Preparation of benzyl and allyl ester derivatives of histidine. Reagents and conditions: a) BnOH or AllylOH, DMAP, DIC, DCM, rt, 30 min. b) TFA, TIS, DCM, rt, 16 h (94% over 2 steps for **3**, 80% over 2 steps for **5**).

Fmoc-His-OBn (3).

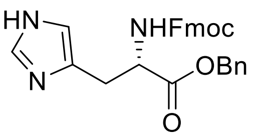

Fmoc-His(Trt)-OH (19.7 g, 31.8 mmol), DMAP (0.39 g, 3.2 mmol) and benzyl alcohol (5.0 ml, 48 mmol) were dissolved in anhydrous DCM (160 ml). DIC (7.4 ml, 48 mmol) was added and the clear solution was stirred at rt for 30 min. For analytical purposes, a small sample was concentrated under reduced pressure and purified by silica gel column chromatography (pentane/EtOAc = 95:5 → 80:20) to yield **2** as a white solid. $\mathbf{R_f}$ = 0.3 (pentane/Et_2O = 50:50). The obtained spectra were in full accordance with literature experimental data.[10] **^{1}H NMR** (400 MHz, $CDCl_3$): δ 7.75 (d, J = 7.5 Hz, 2H), 7.62 (t, J = 6.8 Hz, 2H), 7.42 – 7.19 (m, 14 H), 7.14 – 7.05 (m, 6H), 6.61 (d, J = 8.2 Hz, 1H), 6.50 (d, J = 1.4 Hz, 1H), 5.05 (q, J = 12.2 Hz, 2H), 4.72 – 4.63 (m, 1H), 4.40 – 4.19 (m, 3H), 3.08 (dd, J = 5.0, 2.9 Hz, 2H). **^{13}C NMR** (101 MHz, $CDCl_3$): δ 171.55, 156.36, 144.23, 144.05, 142.35, 141.33, 141.31, 138.97, 136.44, 135.56, 129.88, 128.61, 128.35, 128.21, 128.19, 127.72, 127.70, 127.18, 127.16, 125.52, 125.41, 119.99, 119.76, 75.40, 67.34, 66.98, 54.46, 47.24, 30.13. **HRMS** $[C_{47}H_{40}N_3O_4 + H]^+$ = 710,30028 found, 710,30133 calculated.

Then TFA (22 ml, 286 mmol) and TIS (9.8 ml, 48 mmol) were added and the reaction mixture was stirred at rt overnight. The yellow solution was concentrated under reduced pressure. and co-evaporated with toluene (3x). Purification of the crude residue by silica gel column chromatography (DCM/MeOH = 97.5:2.5→ 90:10) provided title compound **3** as TFA salt. The white solid was dissolved in $CHCl_3$/*i*-PrOH (4:1 v/v, 400 ml), washed with $NaHCO_3$ (sat., 2x 200 ml) and brine (200 ml). The organic fraction was dried over $MgSO_4$, filtered and concentrated under reduced pressure to yield title compound **3** (14 g, 29.9 mmol, 94% over 2 steps) as a white foam. $\mathbf{R_f}$ = 0.25 (DCM/MeOH = 95:5). **^{1}H NMR** (400 MHz, $CDCl_3$): δ 7.71 (d, *J* = 7.6 Hz, 2H), 7.54 (dd, *J* = 7.6, 4.7 Hz, 2H), 7.45 (s, 1H), 7.35 (s, 1H), 7.30 – 7.19 (m, 7H), 6.57 (s, 1H), 6.45 (d, *J* = 8.0 Hz, 1H), 5.16 – 5.05 (m, 2H), 4.69 – 4.61 (m, 1H), 4.37 – 4.24 (m, 2H), 4.17 (t, *J* = 7.3 Hz, 1H), 3.11 (d, *J* = 5.4 Hz, 2H). **^{13}C NMR** (101 MHz, $CDCl_3$): δ 171.69, 156.32, 143.95, 143.83, 141.29, 141.26, 135.53, 135.44, 135.30, 133.88, 128.80, 128.69, 128.60, 128.53, 128.49, 128.44, 128.40, 128.34, 127.75, 127.27,

127.13, 125.25, 125.22, 124.98, 121.07, 120.01, 119.80, 116.12, 107.89, 77.36, 67.31, 67.21, 67.12, 66.97, 54.30, 47.14, 29.51. **HRMS** $[C_{28}H_{25}N_3O_4 + H]^+$ = 468.19146 found, 468,19178 calculated.

Fmoc-His-OAllyl (5).

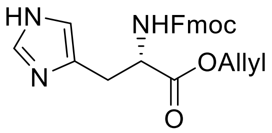

The following synthesis was adopted from literature procedures.[11] Fmoc-His(Trt)-OH (25 g, 40.3 mmol), DMAP (0.49 g, 4.0 mmol) and allyl alcohol (5.5 ml, 81 mmol) were dissolved in anhydrous DCM (134 ml). DIC (6.9 ml, 44 mmol) was added and the clear solution was stirred at rt for 30 min. For analytical purposes, a small sample was concentrated under reduced pressure and purified by silica gel column chromatography (pentane/EtOAc = 90:10 → 70:30) to yield **4** as a white solid. $\mathbf{R_f}$ = 0.5 (pentane/Et_2O = 50:50). The obtained spectra were in full accordance with literature experimental data.[11] **^{1}H NMR** (400 MHz, $CDCl_3$): δ 7.72 (d, J = 8.0 Hz, 2H), 7.66 – 7.57 (m, 2H), 7.43 (d, *J* = 1.4 Hz, 1H), 7.39 – 7.22 (m, 13H), 7.15 – 7.06 (m, 6H), 6.73 (d, J = 8.3 Hz, 1H), 6.59 (d, *J* = 1.4 Hz, 1H), 5.78 (ddt, *J* = 17.3, 10.4, 5.7 Hz, 1H), 5.21 (dd, *J* = 17.2, 1.5 Hz, 1H), 5.10 (dd, *J* = 10.4, 1.3 Hz, 1H), 4.68 (dt, *J* = 8.3, 4.9 Hz, 1H), 4.59 – 4.43 (m, 2H), 4.43 – 4.15 (m, 3H), 3.11 (t, *J* = 4.5 Hz, 2H). **^{13}C NMR** (101 MHz, $CDCl_3$): 171.23, 156.22, 144.05, 143.89, 142.23, 141.17, 141.15, 138.84, 136.34, 131.76, 129.69, 128.05, 128.03, 127.58, 127.03, 127.01, 125.36, 125.25, 119.85, 119.59, 118.45, 77.36, 75.22, 67.15, 65.63, 54.29, 47.09, 30.01. **HRMS** $[C_{43}H_{37}N_3O_4 + H]^+$ = 660.28518 found, 660.28568 calculated.
Then TFA (31.1 ml, 403 mmol) and TIS (12.4 ml, 60.5 mmol) were added and the reaction mixture was stirred at rt overnight. The yellow solution was diluted with DCM (200 ml) and washed with $NaHCO_3$ (sat., 4x 200 ml). The H_2O layer was back-extracted with DCM (1x 100 ml). The combined organic fractions were dried over $MgSO_4$, filtered and concentrated under reduced pressure Purification of the crude residue by silica gel column chromatography (DCM/Acetone = 100:0 → 50:50, 10% steps) provided title compound **5** (13.5 g, 32.3 mmol, 80% over 2 steps) as a white foam. $\mathbf{R_f}$ = 0.3 (DCM/MeOH = 95:5). **^{1}H NMR** (400 MHz, $CDCl_3$): δ 7.73 (d, *J* = 7.6 Hz, 2H), 7.60 – 7.50 (m, 2H), 7.37 (t, *J* = 7.5 Hz, 2H), 7.27 (t, *J* = 7.5 Hz, 2H), 6.77 (s, 1H), 6.37 (d, *J* = 7.5 Hz, 1H), 5.83 (ddt, *J* = 16.5, 11.0, 5.7 Hz, 1H), 5.29 – 5.14 (m, 2H), 4.63 (q, *J* = 6.3 Hz, 1H), 4.57 (d, *J* = 5.8 Hz, 2H), 4.33 (p, *J* = 10.5 Hz, 2H), 4.20 (t, *J* = 7.4 Hz, 1H), 3.14 (d, *J* = 5.5 Hz, 2H). **^{13}C NMR** (101 MHz, $CDCl_3$): 171.58, 156.32, 143.98, 143.88, 141.34, 141.32, 135.35, 134.20, 131.67, 127.79, 127.17, 125.27, 120.05, 118.75, 115.90, 67.21, 66.05, 54.30, 47.20, 29.66. **HRMS** $[C_{24}H_{23}N_3O_4 + H]^+$ = 418.17606 found, 418.17613 calculated.

Fmoc-His(1’-N(τ)-2’,3’,5´-tris-O-benzyl-D-ribofuranosyl)-OBn (8) and Fmoc-His(1’-N(π)-2’,3’,5´-tris-O-benzyl-D-ribofuranosyl)-OBn (9).

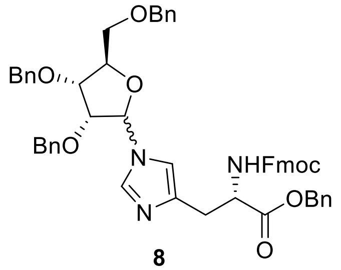

8

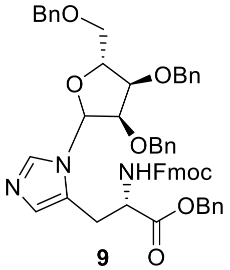

9

Bu_3PO (535 mg, 2.45 mmol) was co-evaporated with toluene (3x), dissolved in anhydrous DCE (10 ml) and cooled to 0 °C. Tf_2O (1 M, 1.15 ml, 1.15 mmol) was added dropwise over 20 min using a syringe pump and the resulting pinkish solution was stirred for 1 h at 0 °C. ^{31}P NMR indicated formation of the diphosphonium salt (δ = 119.5 ppm). **6** (0.21 g, 0.50 mmol) was co-evaporated with toluene (3x), dissolved in anhydrous DCE (10 ml) and DIPEA (0.23 ml) and added to the reaction mixture together with some activated 4A molecular sieves. After 1 h, ^{31}P NMR indicated formation of the activated ribosyl intermediates (δ = 98 & 94 ppm). Compound **3** (0.47 g, 1.0 mmol) was co-evaporated with dioxane (3x),

dissolved in DCE (20 ml) and DIPEA (0.23 ml) and cooled to 0 °C. Activated 4A molecular sieves were added to the histidine solution followed by dropwise addition of the ribosyl intermediate using a syringe pump over 30 min. The reaction mixture was slowly warmed to rt overnight, diluted with DCM (50 ml) and washed with citric acid (1M, 80 ml) and brine (2x 50 ml). The H_2O fractions were back-extracted with DCM (80 ml) and the combined organic fractions were dried over $MgSO_4$, filtered and concentrated under reduced pressure. Purification of the crude residue by silica gel column chromatography (DCM/MeOH = 100:0 → 98:2, 0.5% steps) provided a racemic mixture of title compound **8** (116 mg, 0.133 mmol, 27%) as a white foam and a single anomer (anomeric configuration not determined) of title compound **9** (52 mg, 0.060 mmol, 12%) as white foam.

8: $\mathbf{R_f}$ = 0.6 (DCM/MeOH = 95:5). **LC-MS** R_t = 8.38 min (10-90% MeCN/H_2O, TFA). **^{1}H NMR** (600 MHz, $CDCl_3$): δ 7.76 – 7.71 (m, 6H), 7.71 (s, 1H), 7.60 (dd, *J* = 7.6, 4.6 Hz, 6H), 7.55 (s, 2H), 7.38 – 7.19 (m, 66H), 7.21 – 7.07 (m, 6H), 6.91 (s, 1H), 6.63 (s, 2H), 6.54 (d, *J* = 8.0 Hz, 1H), 6.45 (d, *J* = 8.0 Hz, 2H), 5.69 (d, *J* = 5.3 Hz, 1H), 5.64 (d, *J* = 5.4 Hz, 2H), 5.18 – 5.06 (m, 6H), 4.71 – 4.40 (m, 18H), 4.40 – 4.17 (m, 16H), 4.11 (t, *J* = 5.1 Hz, 1H), 4.06 – 4.00 (m, 4H), 3.63 (dd, *J* = 10.6, 3.2 Hz, 2H), 3.56 (dd, *J* = 10.8, 3.2 Hz, 1H), 3.49 (ddd, *J* = 20.8, 10.7, 2.9 Hz, 3H), 3.16 – 2.96 (m, 6H). **^{13}C NMR** (151 MHz, $CDCl_3$): δ 171.71, 156.39, 156.36, 144.23, 144.20, 144.14, 144.07, 141.35, 141.33, 141.32, 137.93, 137.86, 137.53, 137.10, 137.03, 136.73, 136.04, 135.89, 135.81, 128.71, 128.66, 128.65, 128.64, 128.63, 128.62, 128.59, 128.35, 128.33, 128.25, 128.23, 128.19, 128.14, 128.12, 128.05, 127.96, 127.94, 127.75, 127.73, 127.71, 127.20, 127.18, 125.55, 125.50, 125.47, 125.42, 120.00, 119.96, 119.95, 117.08, 114.44, 88.93, 86.24, 82.41, 82.28, 81.92, 77.64, 76.85, 76.83, 73.77, 73.72, 73.27, 72.96, 72.85, 72.48, 69.91, 69.79, 67.31, 66.99, 66.96, 54.42, 54.29, 47.26, 30.04. **HRMS** $[C_{54}H_{51}N_3O_8 + H]^+$ = 870.37387 found, 870.37489 calculated.

9: $\mathbf{R_f}$ = 0.4 (DCM/MeOH = 95:5). **LC-MS** R_t = 8.33 min (10-90% MeCN/H_2O, TFA). **^{1}H NMR** (600 MHz, $CDCl_3$): δ 8.13 (s, 1H), 7.74 (d, *J* = 7.6 Hz, 2H), 7.55 – 7.50 (m, 2H), 7.41 – 7.14 (m, 22H), 7.08 (dd, *J* = 6.7, 2.8 Hz, 2H), 6.74 (s, 1H), 5.77 (d, *J* = 8.1 Hz, 1H), 5.75 (d, *J* = 5.5 Hz, 1H), 5.15 – 5.06 (m, 2H), 4.65 – 4.57 (m, 2H), 4.53 – 4.31 (m, 5H), 4.28 – 4.20 (m, 4H), 4.17 – 4.11 (m, 2H), 3.48 (dd, *J* = 10.9, 3.2 Hz, 1H), 3.41 (dd, *J* = 10.8, 3.2 Hz, 1H), 3.14 (dd, *J* = 15.6, 6.4 Hz, 1H), 3.00 (dd, *J* = 15.6, 5.9 Hz, 1H). **^{13}C NMR** (151 MHz, $CDCl_3$): δ 171.10, 155.94, 143.96, 143.77, 141.35, 141.33, 139.62, 137.80, 137.50, 136.90, 135.06, 128.71, 128.61, 128.57, 128.56, 128.52, 128.49, 128.16, 128.14, 128.04, 128.00, 127.85, 127.81, 127.78, 127.70, 127.17, 125.88, 125.29, 125.21, 120.03, 83.93, 81.94, 77.40, 76.75, 73.58, 73.30, 72.96, 69.53, 67.53, 67.21, 54.06, 47.15, 27.03. **HRMS** $[C_{54}H_{51}N_3O_8 + H]^+$ = 870.37381 found, 870.37489 calculated.

Fmoc-His (1'-N(τ)-2',3'-bis-O -para-methoxybenzyl-5'-O-tert - diphenylsilyl- ribofuranosyl)-OBn (11) and Fmoc-His(1'-N(π)-2',3'-bis-O-para-methoxybenzyl-5'-O-tert-diphenylsilyl-ribofuranosyl)-OBn (12).

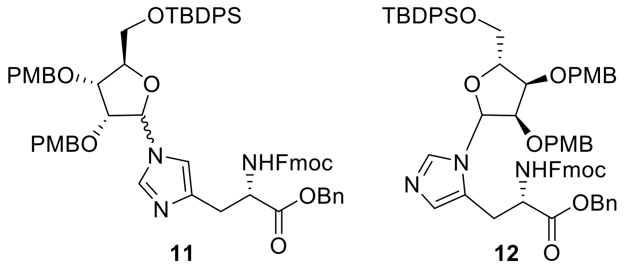

11 **12**

Bu_3PO (535 mg, 2.45 mmol) was co-evaporated with toluene (3x), dissolved in anhydrous DCE (10 ml) and cooled to 0 °C. Tf_2O (1 M, 1.15 ml, 1.15 mmol) was added dropwise over 20 min using a syringe pump and the resulting pinkish solution was stirred for 1 h at 0 °C. ^{31}P NMR indicated formation of the diphosphonium salt (δ = 119.5 ppm). **7** (0.31 g, 0.50 mmol) was co-evaporated with toluene (3x), dissolved in anhydrous DCE (10 ml) and DIPEA (0.23 ml) and added to the reaction mixture together with some activated 4Å molecular sieves. After 1 h, ^{31}P NMR indicated formation of the activated ribosyl intermediates (99 & 94 ppm). **5** (0.47 g, 1.0 mmol) was co-evaporated with dioxane (3x), dissolved in DCE

(20 ml) and DIPEA (0.23 ml) and cooled to 0 °C. Activated 4Å molecular sieves were added to the histidine solution followed by dropwise addition of the ribosyl intermediate using a syringe pump over 30 min. The reaction mixture was slowly warmed to rt overnight, diluted with DCM (50 ml) and washed with citric acid (1M, 80 ml) and brine (2x 50 ml). The H_2O fractions were back-extracted with DCM (80 ml) and the combined organic fractions were dried over $MgSO_4$, filtered and concentrated under reduced pressure. Purification of the crude residue by silica gel column chromatography (DCM/MeOH = 100:0 → 98:2, 0.5% steps) provided an anomeric mixture (1:1.2 ratio, anomeric configuration not determined) of title compound **11** (179 mg, 0.166 mmol, 33%) as a white foam and a single anomer (anomeric configuration not determined) of title compound **12** (0.10 g, 0.093 mmol, 18%) as clear oil.

11: $\mathbf{R_f}$ = 0.6 (DCM/MeOH = 97:3). **LC-MS** R_t = 10.22 min (10-90% MeCN/H_2O, TFA). **^{1}H NMR** (600 MHz, $CDCl_3$): δ 7.74 (s, 1H), 7.74 – 7.69 (m, 4.4H), 7.65 – 7.57 (m, 5.6H), 7.46 – 7.16 (m, 28.6H), 7.06 (dd, *J* = 8.5, 5.5 Hz, 4.4H), 6.95 (s, 1H), 6.89 – 6.77 (m, 8.8H), 6.71 – 6.67 (m, 2.4H), 6.61 (d, *J* = 8.0 Hz, 1H), 5.67 (d, *J* = 5.5 Hz, 1H), 5.65 (d, *J* = 6.3 Hz, 1H), 5.13 (d, *J* = 5.0 Hz, 4.4H), 4.71 (dt, *J* = 8.1, 5.2 Hz, 1H), 4.69 – 4.63 (m, 1.2H), 4.62 – 4.41 (m, 8.8H), 4.41 – 4.16 (m, 17.6H), 4.08 (dd, *J* = 5.1, 2.9 Hz, 1.2H), 3.98 (dd, *J* = 6.3, 5.1 Hz, 1.2H), 3.80 (dd, *J* = 11.5, 3.5 Hz, 1H), 3.78 – 3.68 (m, 14.2H), 3.66 (dd, *J* = 11.4, 2.9 Hz, 1.2H), 3.60 (dd, *J* = 11.5, 2.3 Hz, 1.2H), 3.20 – 3.09 (m, 2H), 3.11 – 2.97 (m, 2.4H), 1.02 (m, 19.8H). **^{13}C NMR** (151 MHz, $CDCl_3$): δ 171.59, 171.56, 159.46, 159.45, 159.44, 159.34, 156.28, 156.22, 144.06, 144.03, 143.94, 143.88, 141.17, 141.15, 141.12, 137.84, 136.47, 135.72, 135.62, 135.57, 135.47, 135.45, 132.85, 132.66, 132.53, 132.47, 130.06, 130.02, 129.94, 129.90, 129.69, 129.61, 129.40, 129.36, 129.34, 128.94, 128.89, 128.46, 128.43, 128.19, 128.06, 127.89, 127.83, 127.81, 127.59, 127.57, 127.56, 127.05, 127.04, 125.41, 125.36, 125.32, 125.27, 125.09, 125.07, 119.86, 119.85, 117.01, 114.30, 113.92, 113.88, 113.87, 113.83, 88.42, 86.23, 83.74, 83.54, 81.31, 77.42, 77.36, 76.01, 75.37, 72.68, 72.42, 72.26, 71.92, 67.14, 66.81, 66.78, 63.67, 63.61, 55.24, 55.19, 55.17, 55.15, 54.30, 54.16, 47.06, 26.86, 26.83, 26.80, 26.79, 19.18, 19.16. **HRMS** $[C_{65}H_{67}N_3O_{10}Si + H]^+$ = 1078.46628 found, 1078.46685 calculated.

12: $\mathbf{R_f}$ = 0.4 (DCM/MeOH = 97:3). **LC-MS** R_t = 10.22 min (10-90% MeCN/H_2O, TFA). **^{1}H NMR** (600 MHz, $CDCl_3$): δ 8.19 – 8.16 (m, 1H), 7.72 (d, *J* = 7.6 Hz, 2H), 7.60 – 7.44 (m, 6H), 7.40 – 7.18 (m, 17H), 7.01 (d, *J* = 8.6 Hz, 2H), 6.84 (d, *J* = 8.6 Hz, 2H), 6.75 (d, *J* = 8.7 Hz, 2H), 5.83 (d, *J* = 8.2 Hz, 1H), 5.69 (d, *J* = 5.7 Hz, 1H), 5.16 – 5.08 (m, 2H), 4.63 (dt, *J* = 8.2, 6.2 Hz, 1H), 4.58 (d, *J* = 11.5 Hz, 1H), 4.44 (d, *J* = 11.4 Hz, 1H), 4.37 – 4.17 (m, 6H), 4.13 (dd, *J* = 10.5, 7.4 Hz, 1H), 4.06 (d, *J* = 7.3 Hz, 1H), 3.81 – 3.66 (m, 7H), 3.58 (dd, *J* = 11.4, 2.8 Hz, 1H), 3.11 (dd, *J* = 15.7, 6.7 Hz, 1H), 3.00 (dd, *J* = 15.7, 5.7 Hz, 1H). **^{13}C NMR** (151 MHz, $CDCl_3$): δ 171.20, 159.46, 159.37, 155.90, 143.83, 143.74, 141.25, 141.24, 135.58, 135.53, 135.01, 132.87, 132.83, 129.95, 129.92, 129.75, 129.54, 128.95, 128.79, 128.65, 128.53, 128.44, 127.83, 127.83, 127.12, 127.10, 127.07, 125.85, 125.20, 125.15, 119.96, 113.91, 83.71, 83.43, 77.41, 76.17, 72.91, 72.57, 67.45, 67.13, 63.65, 55.30, 55.29, 53.86, 47.00, 26.97, 26.86, 19.24. **HRMS** $[C_{65}H_{67}N_3O_{10}Si + H]^+$ = 1078.46596 found, 1078.46685 calculated.

Fmoc-His(1'-N(τ)-2',3'-bis-O -para-methoxybenzyl-5'-O -tert-diphenylsilyl-ribofuranosyl)-OAllyl (13) and Fmoc-His(1'-α-N(π)-2',3'-bis-O-para-methoxybenzyl-5'-O-tert-diphenylsilyl-ribofuranosyl)-OAllyl (14).

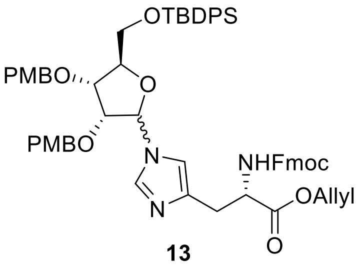

13

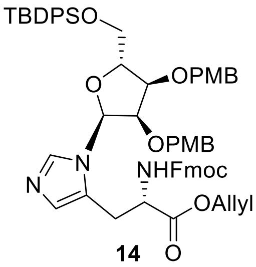

14

Bu_3PO (535 mg, 2.45 mmol) was co-evaporated with toluene (3x), dissolved in anhydrous DCE (10 ml) and cooled to 0 °C. Tf_2O (1 M, 1.15 ml, 1.15 mmol) was added dropwise over 20 min using a syringe pump and the resulting pinkish solution was stirred for 1 h at 0 °C. ^{31}P NMR indicated formation of the diphosphonium salt (δ = 119.5 ppm). **7** (0.31 g, 0.50 mmol) was co-evaporated with toluene (3x), dissolved in anhydrous DCE (10 ml) and DIPEA (0.23 ml) and added to the reaction mixture together with some activated 4Å molecular sieves. After 1 h, ^{31}P NMR indicated formation of the activated ribosyl intermediates (98 & 93 ppm). **5** (0.42 g, 1.0 mmol) was co-evaporated with dioxane (3x), dissolved in DCE (20 ml) and DIPEA (0.23 ml) and cooled to 0 °C. Activated 4Å molecular sieves were added to the histidine solution followed by dropwise addition of the ribosyl intermediate using a syringe pump over 30 min. The reaction mixture was slowly warmed to rt overnight, diluted with DCM (50 ml) and washed with citric acid (1M, 80 ml) and brine (2x 50 ml). The H_2O fractions were backextracted with DCM (80 ml) and the combined organic fractions were dried over $MgSO_4$, filtered and concentrated under reduced pressure. Purification of the crude residue by silica gel column chromatography (DCM/acetone = 100:0 → 90:10, 2.5% steps) provided an racemic mixture of title compound **13** (0.11 g, 0.11 mmol, 21%) as an off-white foam and title compound **14** (0.12 g, 0.12 mmol, 23%) an off-white foam.

<u>13:</u> R_f = 0.5 (DCM/MeOH = 95:5). **LC-MS** R_t = 9.28 min (10-90% MeCN/H_2O, TFA). **^{1}H NMR** (600 MHz, $CDCl_3$): δ 7.72 (t, *J* = 6.6 Hz, 5H), 7.64 – 7.57 (m, 13H), 7.45– 7.17 (m, 24H), 7.12 (s, 1H), 7.07 (dd, *J* = 21.9, 8.6 Hz, 4H), 6.88 – 6.77 (m, 9H), 6.61 (d, *J* = 8.0 Hz, 1H), 6.55 (d, *J* = 8.0 Hz, 1H), 5.88 – 5.79 (m, 2H), 5.71 (d, *J* = 5.6 Hz, 1H), 5.68 (d, *J* = 6.3 Hz, 1H), 5.29 – 5.13 (m, 4H), 4.70 – 4.32 (m, 13H), 4.31 – 4.16 (m, 11H), 4.09 (dd, *J* = 5.2, 2.9 Hz, 1H), 4.01 (t, *J* = 5.7 Hz, 1H), 3.76 (dd, *J* = 22.7, 11.9 Hz, 13H), 3.69 – 3.58 (m, 3H), 3.19 – 2.98 (m, 4H), 1.08 – 0.96 (m, 18H). **^{13}C NMR** (151 MHz, $CDCl_3$): δ 171.50, 171.43, 159.55, 159.53, 159.41, 156.31, 156.25, 144.10, 144.07, 144.03, 143.97, 141.24, 141.23, 141.22, 138.02, 136.66, 135.90, 135.62, 135.60, 135.58, 135.53, 135.51, 132.93, 132.74, 132.61, 132.55, 131.94, 131.87, 130.11, 130.06, 130.01, 129.98, 129.94, 129.74, 129.64, 129.56, 129.44, 129.41, 129.00, 128.97, 127.94, 127.91, 127.87, 127.85, 127.68, 127.64, 127.63, 127.61, 127.60, 127.10, 127.08, 125.45, 125.41, 125.38, 125.33, 119.95, 119.91, 119.87, 118.32, 118.27, 117.18, 114.28, 114.03, 114.00, 113.95, 113.93, 113.90, 88.47, 86.26, 83.89, 83.63, 81.42, 77.52, 76.14, 75.46, 72.76, 72.52, 72.38, 71.99, 67.18, 65.74, 65.73, 63.74, 63.73, 55.31, 55.29, 55.27, 55.26, 55.24, 54.35, 54.18, 47.14, 30.05, 29.91, 26.93, 26.90, 26.86, 19.23. **HRMS** $[C_{61}H_{65}N_3O_{10}Si + H]^+$ = 1028.45017 found, 1028.45120 calculated.

<u>14:</u> R_f = 0.4 (DCM/MeOH = 95:5). **LC-MS** R_t = 9.28 min (10-90% MeCN/H_2O, TFA). **^{1}H NMR** (600 MHz, $CDCl_3$): 8.19 (s, 1H), 7.71 (d, *J* = 7.6 Hz, 2H), 7.57 (d, *J* = 28.1 Hz, 2H), 7.52 – 7.46 (m, 4H), 7.41 – 7.28 (m, 8H), 7.28 – 7.18 (m, 4H), 7.04 (d, *J* = 8.6 Hz, 2H), 6.86 – 6.81 (m, 3H), 6.78 (d, *J* = 8.6 Hz, 2H), 5.88 – 5.77 (m, 2H), 5.72 (d, *J* = 5.7 Hz, 1H), 5.28 – 5.16 (m, 2H), 4.65 – 4.54 (m, 4H), 4.45 (d, *J* = 11.5 Hz, 1H), 4.39 – 4.29 (m, 3H), 4.25 (d, *J* = 11.5 Hz, 3H), 4.28 – 4.23 (m, 2H), 4.21 (q, *J* = 3.3 Hz, 1H), 4.17 (dd, *J* = 10.6, 7.4 Hz, 1H), 4.10 (t, *J* = 7.3 Hz, 1H), 3.79 – 3.69 (m, 7H), 3.60 (dd, *J* = 11.4, 2.8 Hz, 1H) 3.11 (dd, *J* = 15.6, 6.7 Hz, 1H), 3.01 (dd, *J* = 15.6, 5.8 Hz, 1H). **^{13}C NMR** (151 MHz, $CDCl_3$): δ 171.02, 159.46, 159.36, 155.87, 143.80, 143.74, 141.23, 135.56, 135.50, 132.87, 132.83, 131.36, 129.91, 129.88, 129.71, 129.51, 128.96, 127.85, 127.81, 127.80, 127.76, 127.68, 127.67, 127.09, 127.04, 125.86, 125.16, 125.13, 119.93, 119.15, 113.90, 113.89, 83.71,

83.42, 77.45, 76.16, 72.91, 72.55, 67.08, 66.23, 63.67, 55.26, 55.25, 53.77, 47.00, 26.85, 19.22. **HRMS** $[C_{61}H_{65}N_3O_{10}Si + H]^+$ = 1028.45031 found, 1028.45120 calculated.

Fmoc-His(1'-β-N(τ)-2',3'-bis-O-para-methoxybenzyl-5'-O-tert-diphenylsilyl-ribofuranosyl)-OH (15) and Fmoc-His(1'-α-N(τ)-2',3'-bis-O-para-methoxybenzyl-5'-O-tert-diphenylsilyl-ribofuranosyl)-OH (16).

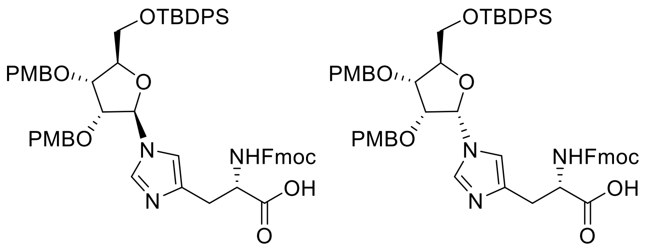

15 **16**

Method I: Compounds **11** (144 mg, 0.134 mmol) were dissolved in THF (7.5 ml) and cooled to 0 °C before adding a solution of LiOH (6.4 mg, 0.19 mmol) in H_2O (2.5 ml). After 1.5 h at 0 °C, the reaction mixture was diluted with DCM (50 ml) and washed with citric acid (10wt%, 2x 40 ml). The H_2O layer was backextracted with DCM (2x 40 ml). The combined organic fractions were dried over $MgSO_4$, filtered and concentrated under reduced pressure. Purification of the crude residue by silica gel column chromatography (DCM/MeOH + 1% AcOH = 100:0 → 90:10) provided title compound **15** (30 mg, 0.030 mmol, 23%) as a white foam and **16** (32 mg, 0.030 mmol, 24%) as a white foam.

Method II: Compounds **13** (0.45 g, 0.44 mmol) and DMBA (82 mg, 0.52 mmol) were dissolved in anhydrous DCM (15 ml) and bubbled with argon gas for 10 min before adding $Pd(PPh_3)_4$ (15 mg, 0.013 mmol). The yellow solution was stirred for 1 h at rt, diluted with DCM (100 ml) and washed with citric acid (10wt%, 2x 100 ml). The H_2O layer was backextracted with DCM (2x 50 ml). The combined organic fractions were dried over $MgSO_4$, filtered and concentrated under reduced pressure. Purification of the crude residue by silica gel column chromatography (DCM/MeOH + 1% AcOH = 100:0 → 95:5) provided title compound **15** (0.17 g, 0.17 mmol, 39%) as a white foam and **16** (0.17 g, 0.17 mmol, 39%) as a white foam.

15: $\mathbf{R_f}$ = 0.3 (DCM/MeOH + 1% AcOH = 95:5). **LC-MS** R_t = 6.78 min (50-90% MeCN/H_2O, TFA). $\mathbf{^1H}$ **NMR** (600 MHz, $CDCl_3$): δ 7.83 (s, 1H), 7.74 (d, *J* = 7.6 Hz, 2H), 7.60 – 7.50 (m, 6H), 7.41 – 7.26 (m, 10H), 7.20 (d, *J* = 8.6 Hz, 2H), 7.04 (d, *J* = 8.3 Hz, 2H), 6.87 – 6.84 (m, 3H), 6.77 (d, *J* = 8.6 Hz, 2H), 5.93 (d, *J* = 6.0 Hz, 1H), 5.65 (d, *J* = 6.3 Hz, 1H), 4.54 – 4.44 (m, 3H), 4.41 (d, *J* = 11.9 Hz, 1H), 4.37 (dd, *J* = 10.5, 7.3 Hz, 1H), 4.33 (d, *J* = 11.8 Hz, 1H), 4.23 – 4.15 (m, 3H), 4.02 – 3.99 (m, 1H), 3.96 (t, *J* = 5.7 Hz, 1H), 3.78 (s, 3H), 3.76 – 3.70 (m, 4H), 3.62 (dd, *J* = 11.6, 3.0 Hz, 1H), 3.32 (dd, *J* = 15.0, 2.9 Hz, 1H), 3.07 (dd, *J* = 15.1, 6.6 Hz, 1H), 0.98 (s, 9H). $\mathbf{^{13}C}$ **NMR** (214 MHz, $CDCl_3$): δ 173.26, 159.66, 159.59, 155.68, 144.11, 143.94, 141.33, 141.31, 135.60, 135.54, 134.57, 132.67, 132.56, 130.14, 130.12, 129.73, 129.57, 129.56, 129.43, 129.10, 128.76, 128.29, 127.97, 127.74, 127.72, 127.20, 127.16, 125.34, 120.00, 115.25, 114.11, 114.09, 114.03, 88.99, 84.24, 81.37, 75.57, 72.43, 72.10, 66.93, 63.65, 55.36, 55.28, 53.65, 47.25, 29.76, 29.75, 27.00, 26.94, 26.91, 19.21. **HRMS** $[C_{58}H_{61}N_3O_{10}Si + H]^+$ = 988.41908 found, 988.41990 calculated.

16: $\mathbf{R_f}$ = 0.2 (DCM/MeOH + 1% AcOH = 95:5). **LC-MS** R_t = 6.96 min (50-90% MeCN/H_2O, TFA). $\mathbf{^1H}$ **NMR** (600 MHz, $CDCl_3$): δ 8.15 (s, 1H), 7.72 (d, *J* = 7.7 Hz, 2H), 7.62 – 7.53 (m, 6H), 7.46 – 7.40 (m, 2H), 7.40 – 7.29 (m, 6H), 7.26 (t, *J* = 7.6 Hz, 2H), 7.11 (d, *J* = 8.1 Hz, 2H), 7.01 (s, 1H), 6.99 (d, *J* = 8.1 Hz, 2H), 6.80 (d, *J* = 8.2 Hz, 2H), 6.75 (d, *J* = 8.2 Hz, 2H), 6.12 (s, 1H), 5.60 (d, *J* = 5.7 Hz, 1H), 4.54 – 4.47 (m, 2H), 4.44 – 4.27 (m, 6H), 4.23 (s, 1H), 4.19 (t, *J* = 7.5 Hz, 1H), 4.14 – 4.10 (m, 1H), 3.73 (s, 3H), 3.71 – 3.66 (m, 4H), 3.54 (d, *J* = 11.6 Hz, 1H), 3.44 (d, *J* = 14.7 Hz, 1H), 3.23 (dd, *J* = 15.1, 6.4 Hz, 1H), 1.00 (s, 9H). $\mathbf{^{13}C}$ **NMR** (214 MHz, $CDCl_3$): δ 173.72, 159.69, 159.48, 155.73, 144.19, 144.10, 141.35, 141.30, 137.93, 136.07, 135.60, 135.55, 133.06, 132.88, 132.64, 130.08, 130.06, 129.68, 129.63, 129.56, 129.34, 129.11, 128.71, 128.30, 127.95, 127.92, 127.68, 127.13, 125.37, 125.36, 125.34, 119.96, 119.95, 118.00, 114.14, 113.98, 86.97, 84.93, 77.96, 77.36,

75.95, 73.02, 72.51, 66.76, 63.82, 55.30, 55.23, 54.01, 47.33, 26.93, 19.27. **HRMS** $[C_{58}H_{61}N_3O_{10}Si + H]^+$ = 988.41931 found, 988.41990 calculated.

Fmoc-His(1'-α-N(τ)-5'-O-tert-diphenylsilyl-ribofuranosyl)-OAllyl (17).

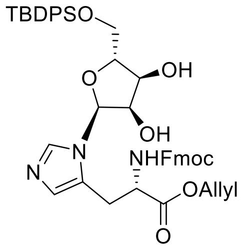

Compound **14** (0.17 g, 0.16 mmol) was dissolved in HFIP (3.3 ml) and cooled to 0 ºC before adding HCl (0.2 M in HFIP, 0.33 ml). After 25 min, additional HCl (0.2 M in HFIP, 0.33 ml) was added and the resulting deep red solution was stirred for another 10 min. The reaction mixture was diluted with $NaHCO_3$ (sat., 30 ml) and extracted with DCM (3x 30 ml). The combined organic fractions were dried over $MgSO_4$, filtered and concentrated under reduced pressure. Purification of the crude residue by silica gel column chromatography (DCM/MeOH = 99:1 → 95:5) provided title compound **17** (46 mg, 0.058 mmol, 36%) as an off-white foam. **R$_f$** = 0.3 (DCM/MeOH = 90:10). **LC-MS** R_t = 8.00 min (10-90% MeCN/H_2O, TFA). **^{1}H NMR** (850 MHz, $CDCl_3$): δ 7.73 (s, 1H), 7.69 (d, *J* = 7.6 Hz, 2H), 7.64 (t, *J* = 7.5 Hz, 4H), 7.50 (d, *J* = 4.5 Hz, 1H), 7.41 – 7.30 (m, 8H), 7.26 – 7.21 (m, 2H), 6.66 (s, 1H), 6.11 (d, *J* = 8.0 Hz, 1H), 5.83 (d, *J* = 5.1 Hz, 1H), 5.82 – 5.75 (m, 1H), 5.26 (s, 1H), 5.24 (d, *J* = 17.1 Hz, 1H), 5.16 (d, *J* = 10.5 Hz, 1H), 4.60 – 4.55 (m, 3H), 4.53 (t, *J* = 5.6 Hz, 1H), 4.47 (t, *J* = 4.8 Hz, 1H), 4.35 – 4.30 (m, 2H), 4.21 (dd, *J* = 10.7, 7.4 Hz, 1H), 4.11 (t, *J* = 7.4 Hz, 1H), 3.83 (dd, *J* = 11.6, 3.1 Hz, 1H), 3.74 (dd, *J* = 11.6, 2.9 Hz, 1H), 3.13 (dd, *J* = 15.9, 5.6 Hz, 1H), 3.04 (dd, *J* = 15.8, 7.6 Hz, 1H), 1.04 (s, 9H). **^{13}C NMR** (214 MHz, $CDCl_3$): δ 171.14, 156.14, 143.83, 143.77, 141.29, 137.61, 135.62, 132.94, 132.92, 131.36, 129.98, 129.93, 127.91, 127.89, 127.80, 127.77, 127.15, 127.13, 126.61, 126.23, 125.24, 125.21, 120.01, 119.23, 86.76, 85.80, 72.36, 71.26, 67.20, 66.40, 64.20, 53.74, 53.55, 47.06, 26.99, 26.95, 26.93, 19.29. **HRMS** $[C_{45}H_{49}N_3O_8Si + H]^+$ = 788.33574 found, 788.33617 calculated.

Fmoc-His(1'-α-N(τ)-2',3'-bis-O-para-methoxybenzyl-5'-O-tert-diphenylsilyl-ribofuranosyl)-OH (18).

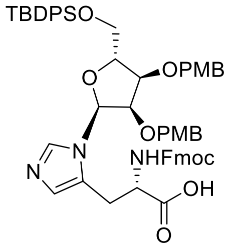

Method I: **12** (0.10 g, 0.093 mmol) was dissolved in THF (7.5 ml) and cooled to 0 °C before adding a solution of LiOH (4.4 mg, 0.19 mmol) in H_2O (2.5 ml). After 1.5 h at 0 °C, the reaction mixture was diluted with DCM (50 ml) and washed with HCl (1 M, 2x 50 ml). The H_2O layer was backextracted with DCM (50 ml). The combined organic fractions were dried over $MgSO_4$, filtered and concentrated under reduced pressure. Purification of the crude residue by silica gel column chromatography (DCM/MeOH + 1% AcOH = 100:0 → 95:5) provided title compound **18** (43 mg, 0.044 mmol, 47%) as a white foam.

Method II: **14** (0.12 g, 0.12 mmol) and DMBA (31 mg, 0.20 mmol) were dissolved in anhydrous DCM (7.5 ml) and bubbled with argon gas for 10 min before adding $Pd(PPh_3)_4$ (13 mg, 0.012 mmol). The yellow solution was stirred for 30 min at rt, diluted with DCM (40 ml) and washed with citric acid (10wt%, 2x 40 ml). The water layer was backextracted with DCM (2x 40 ml). The combined organic fractions were dried over $MgSO_4$, filtered and concentrated under reduced pressure. Purification of the crude residue by silica gel column chromatography (DCM/MeOH + 1% AcOH = 100:0 → 90:10) provided title compound **18** (0.10 g, 0.10 mmol, 89%) as an off-white foam.

R$_f$ = 0.20 (DCM/MeOH + 1% AcOH = 95:5). **LC-MS** R_t = 8.70 min (10-90% MeCN/H_2O, TFA). **^{1}H NMR** (850 MHz, Acetone): δ 8.51 (s, 1H), 7.80 – 7.57 (m, 8H), 7.46 – 7.11 (m, 15H), 6.82 (dd, *J* = 57.5, 8.3 Hz, 4H), 6.33 (s, 1H), 4.69 – 4.07 (m, 11H), 3.79 – 3.59 (m, 8H), 3.44 – 3.39 (m, 1H), 3.33 – 3.24 (m, 1H), 0.99 (s, 9H). **^{13}C NMR** (214 MHz, Acetone): δ 174.23, 160.27, 160.21, 156.82, 144.95, 144.88, 141.90, 138.47, 136.28, 136.24, 135.58, 133.81, 133.69, 130.68, 130.66, 130.50, 130.37, 130.25, 129.48, 128.65, 128.38, 127.90,

126.17, 126.17, 124.54, 120.61, 114.52, 114.51, 85.36, 84.45, 78.17, 76.96, 73.38, 72.67, 67.25, 64.39, 55.46, 55.43, 54.98, 47.81, 27.21, 19.65. **HRMS** $[C_{58}H_{61}N_3O_{10}Si + H]^+$ = 988.42016 found, 988.41990 calculated.

N(τ)-β-ADPr-His: TF[H]^{ADPr}GAGLVVPVDK (26).

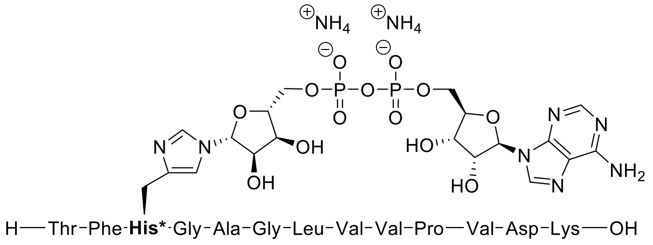

Title compound **26** was synthesized according to the general resin-based procedure described above on a 50 µmol scale by incorporating the β-N(τ)-ADP-ribosylated histidine **15**. Purification of half of the crude residue (25 µmol) by preparative HPLC (NH_4OAc buffer) and subsequent lyophilization yielded title compound **26** (13.0 mg, 6.93 µmol, 28%) as a fluffy white powder. **LC-MS** R_t = 4.29 min (10-50% $MeCN/H_2O$, TFA). **^{1}H NMR** (400 MHz, D_2O): δ 8.73 (d, *J* = 1.6 Hz, 1H), 8.44 (s, 1H), 8.17 (s, 1H), 7.42 (s, 1H), 7.25 – 7.14 (m, 3H), 7.12 (d, *J* = 7.2 Hz, 2H), 6.03 (d, *J* = 5.7 Hz, 1H), 5.74 (d, *J* = 4.5 Hz, 1H). $[C_{77}H_{119}N_{21}O_{30}P_2 + 3H]^{3+}$ = 627.60438 found, 627.60417 calculated.

N(τ)-α-ADPr-His: TF[H]^{ADPr}GAGLVVPVDK (27).

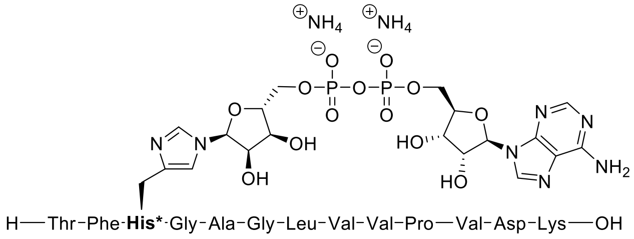

Title compound **27** was synthesized according to the general resin-based procedure described above on a 50 µmol scale by incorporating the α-N(τ)-ADP-ribosylated histidine **16**. Purification of half of the crude residue (25 µmol) by preparative HPLC (AcOH buffered) and subsequent lyophilization yielded title compound **27** (16.2 mg, 8.62 µmol, 35%) as a fluffy white powder. **LC-MS** R_t = 4.26 min (10-50% $MeCN/H_2O$, TFA). **^{1}H NMR** (400 MHz, D_2O): δ 8.64 (s, 1H), 8.48 (s, 1H), 8.19 (s, 1H), 7.30 (s, 1H), 7.27 – 7.21 (m, 3H), 7.15 (d, *J* = 6.2 Hz, 2H), 6.07 (d, *J* = 6.1 Hz, 1H), 6.04 (d, *J* = 5.3 Hz, 1H). **^{31}P NMR** (162 MHz, D_2O): δ -10.41, -10.54, -10.71, -10.84. **HRMS** $[C_{77}H_{119}N_{21}O_{30}P_2 + 2H]^{2+}$ = 940.90222 found, 940.90262 calculated; $[C_{77}H_{119}N_{21}O_{30}P_2 + 3H]^{3+}$ = 627.60418 found, 627.60417 calculated.

N(π)-α-ADPr-His: TF[H]^{ADPr}GAGLVVPVDK (28).

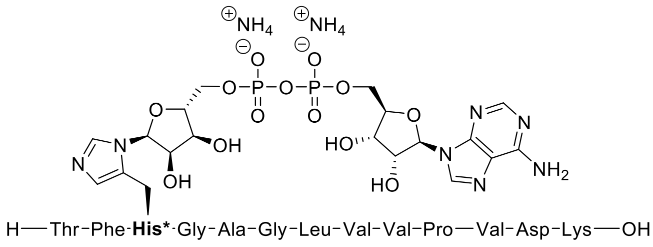

Title compound **28** was synthesized according to the general resin-based procedure described above on a 50 µmol scale by incorporating the N(π)-ADP-ribosylated histidine **18**. Purification of half of the crude residue (25 µmol) by preparative HPLC (AcOH buffered) and subsequent lyophilization yielded title compound **28** (12.9 mg, 6.91 µmol, 28%) as a fluffy white powder. **LC-MS** R_t = 4.24 min (10-50% $MeCN/H_2O$, TFA). **^{1}H NMR** (400 MHz, D_2O): δ 8.68 (s, 1H), 8.47 (s, 1H), 8.17 (s, 1H), 7.28 – 7.21 (m, 3H), 7.16 (d, *J* = 7.4 Hz, 2H), 7.05 (s, 1H), 6.12 (d, *J* = 5.2 Hz, 1H), 6.05 (d, *J* = 6.0 Hz, 1H). **^{31}P NMR** (162 MHz, D_2O): δ -10.47, -10.60, -10.69, -10.82. **HRMS** $[C_{77}H_{119}N_{21}O_{30}P_2 + 2H]^{2+}$ = 940.90226 found, 940.90262 calculated; $[C_{77}H_{119}N_{21}O_{30}P_2 + 3H]^{3+}$ = 627.60421 found, 627.60417 calculated.

Chemical stability assay

A stock solution of peptides **26-28** (1 mg/ml) was prepared in milliQ grade water. The stock solution was mixed with an equal volume of an aqueous solution of TFA (0.2 M), NH_2OH (1 M) or NaOH (0.2 M) and shaken at rt. Samples (20 µl) were taken at various time points for LC-MS analysis. In case of TFA and NH_2OH, samples were simply diluted with milliQ grade water (80 µl) before injection. NaOH samples were acidified with TFA (0.2 M, 40 µl) and diluted with milliQ grade water (40 µl) prior to analysis. Peptide degradation was monitored by UV detection and mass fragments. The resulting peak areas in the UV-trace (260 nm) were quantified in Thermo Xcalibur Qual Browser using the "Auto Calc Initial Event" option. The time-course LC-MS data of **28** was imported to GraphPad Prism 9.0.0 and subjected to a "Nonlinear regression (curve fit)". Selecting the "Exponential (one-phase decay)" option provided a trendline including the coefficient of determination (R^2).

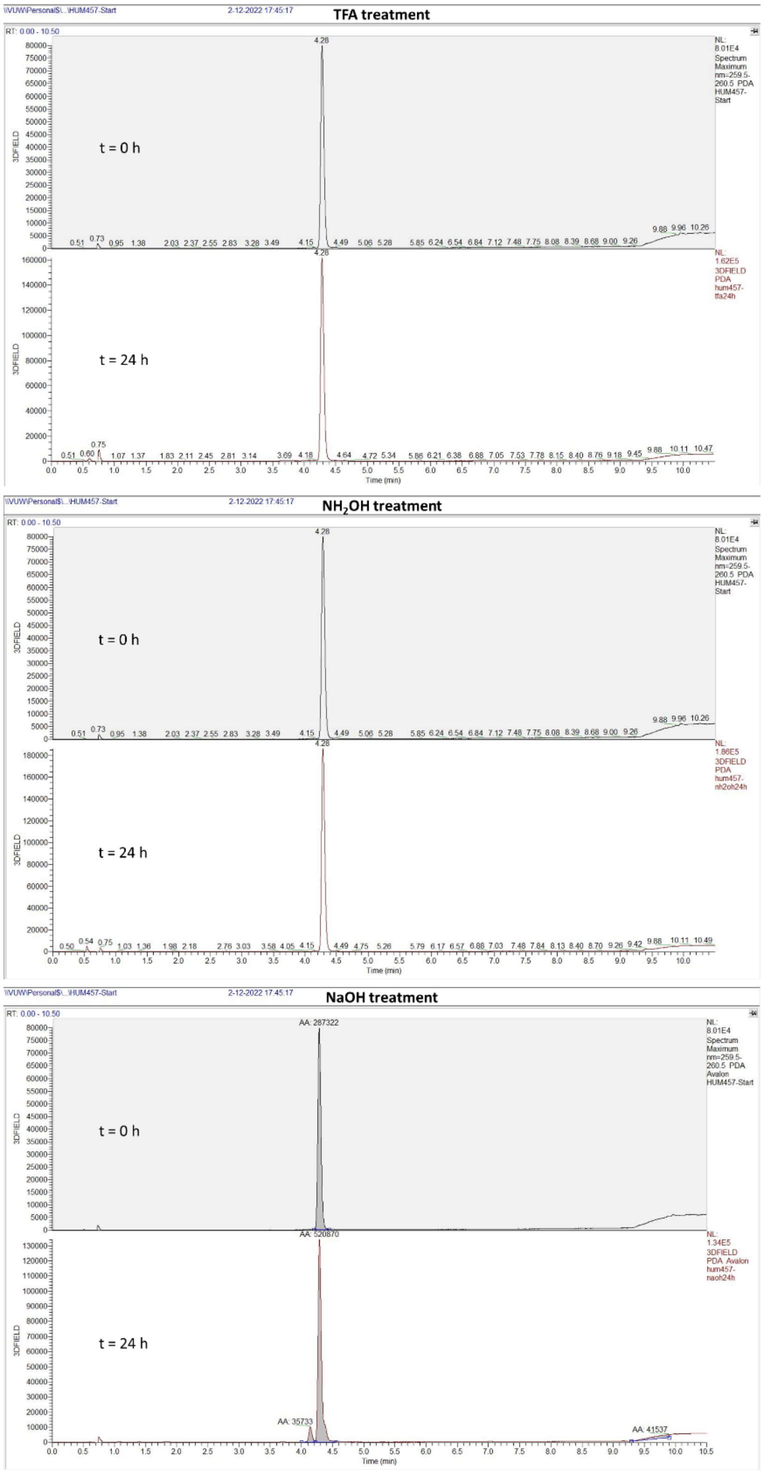

Figure S1. UV-trace (260 nm) of the obtained LC-MS spectra after treating β-configured N(τ)-ADP-ribosylated HPF1 fragment **26** with TFA (0.1 M, top) or NH_2OH (0.5 M, mid) or NaOH (0.1 M, bot) at various time points.

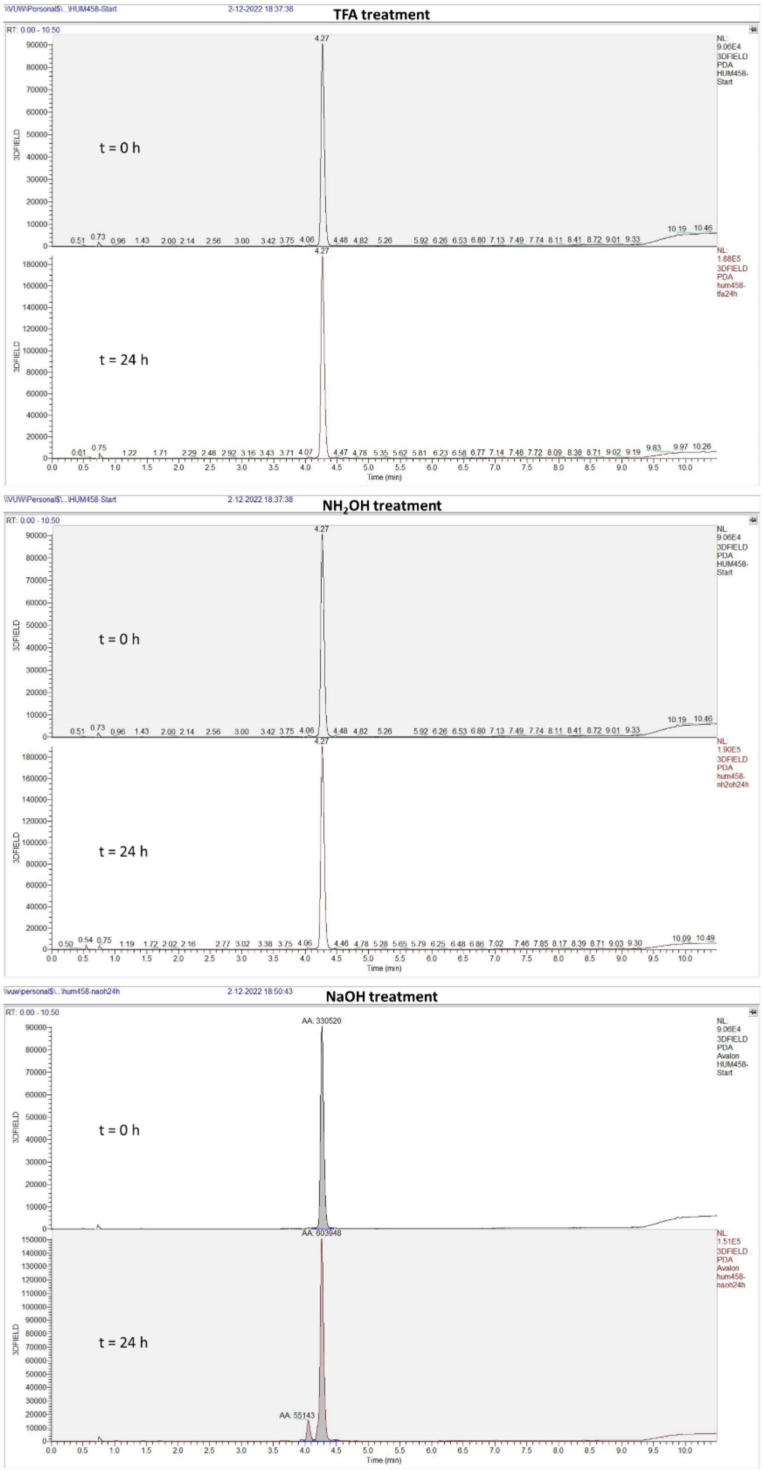

Figure S2. UV-trace (260 nm) of the obtained LC-MS spectra after treating α-configured N(τ)-ADP-ribosylated HPF1 fragment **27** with TFA (0.1 M, top) or NH_2OH (0.1 M, mid) or NaOH (0.5 M, bot) at various time points.

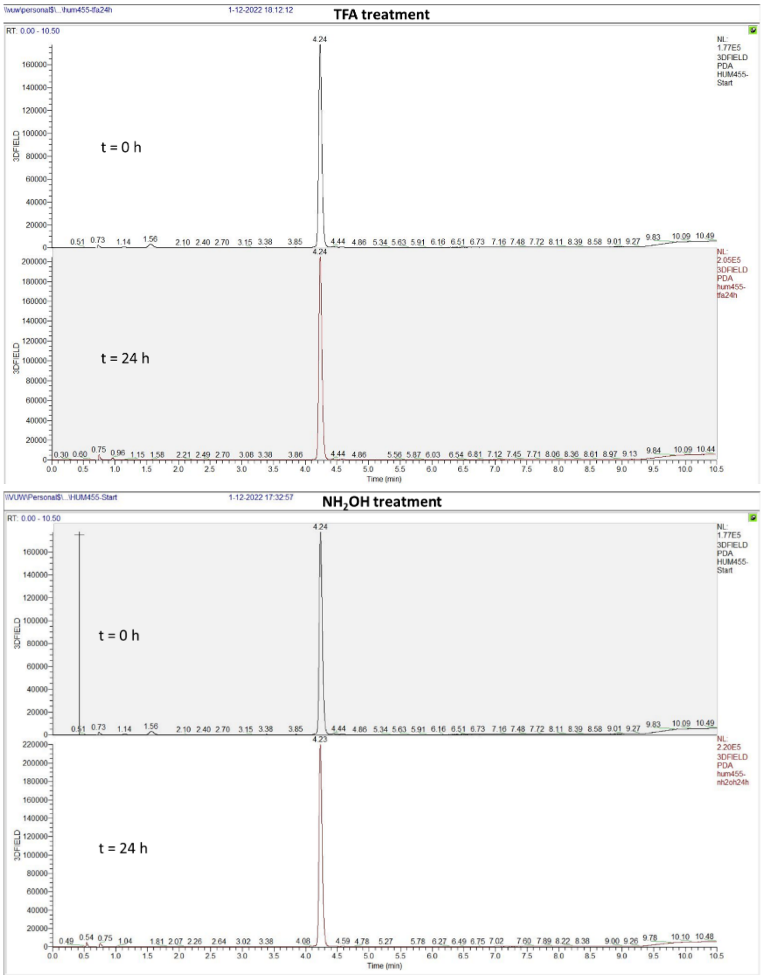

Figure S3. UV-trace (260 nm) of the obtained LC-MS spectra after treating α-configured N(π)-ADP-ribosylated HPF1 fragment **28** with TFA (0.1 M, top) or NH_2OH (0.5 M, bottom) at various time points.

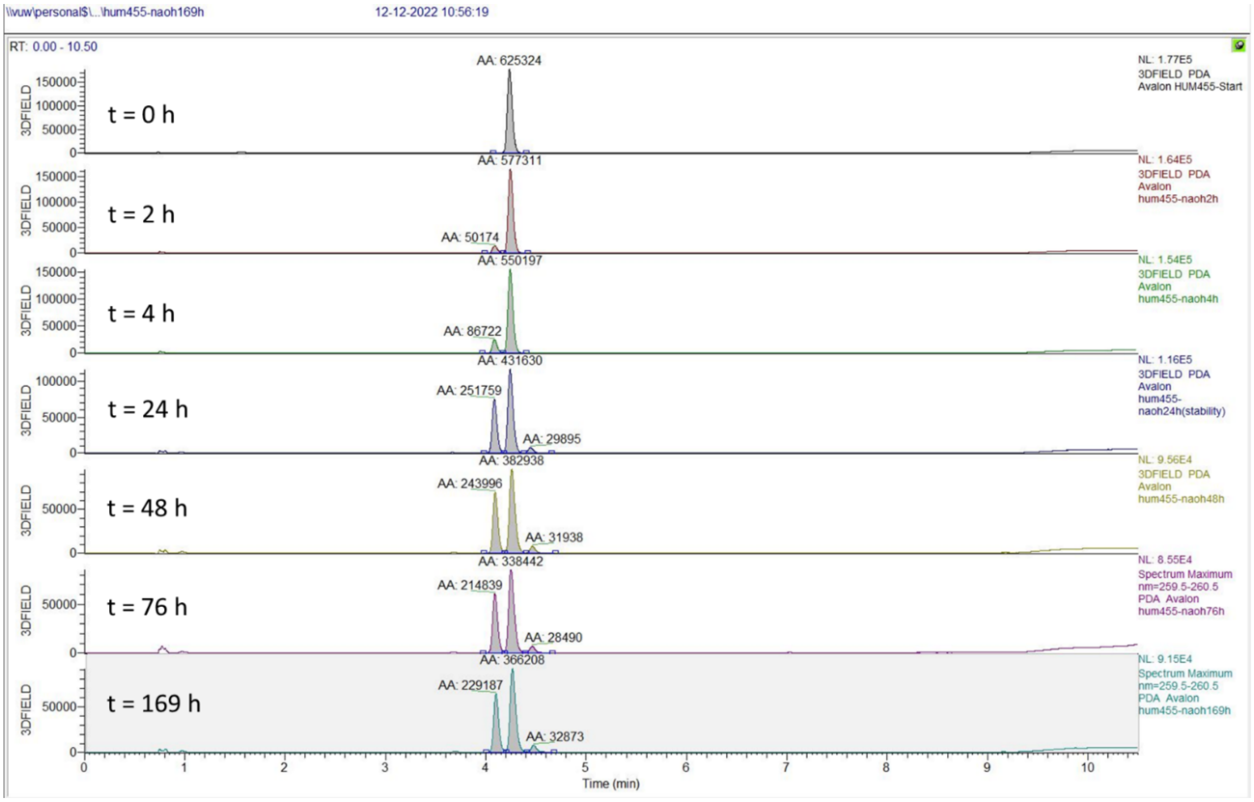

Figure S4. UV-trace (260 nm) of the obtained LC-MS spectra after treating α-configured N(π)-ADP-ribosylated HPF1 fragment **28** with 0.1 M NaOH at various time points.

Table S1. Residual peptide **28** after treatment with 0.1 M NaOH derived from the data presented in figure S4.

Time (h)	Intact peptide (%)	Accumulated isomers (%)
0	100	0
2	92	8
4	86	14
24	61	39
48	58	42
76	58	42
169	58	42

References

[1] P. Fontana, J. J. Bonfiglio, L. Palazzo, E. Bartlett, I. Matic, I. Ahel, *Elife* **2017**, *6*, e28533.

[2] J. G. Rack, R. Morra, E. Barkauskaite, R. Kraehenbuehl, A. Ariza, Y. Qu, M. Ortmayer, O. Leidecker, D. R. Cameron, I. Matić, A. Y. Peleg, D. Leys, A. Traven, I. Ahel, *Mol Cell* **2015**, *59*, 309–20.

[3] D. Slade, M. S. Dunstan, E. Barkauskaite, R. Weston, P. Lafite, N. Dixon, M. Ahel, D. Leys, I. Ahel, *Nature* **2011**, *477*, 616–620.

[4] M. Schuller, R. E. Butler, A. Ariza, C. Tromans-Coia, G. Jankevicius, T. D. W. Claridge, S. L. Kendall, S. Goh, G. R. Stewart, I. Ahel, *Nature* **2021**, *596*, 597–602.

[5] J. Voorneveld, J. G. M. Rack, I. Ahel, H. S. Overkleeft, G. A. van der Marel, D. V. Filippov, *Org. Lett.* **2018**, *20*, 4140–4143.

[6] L. Palazzo, B. Thomas, A.-S. Jemth, T. Colby, O. Leidecker, K. L. H. Feijs, R. Zaja, O. Loseva, J. C. Puigvert, I. Matic, T. Helleday, I. Ahel, *Biochem. J.* **2015**, *468*, 293–301.

[7] H. A. V. Kistemaker, A. P. Nardozza, H. S. Overkleeft, G. A. van der Marel, A. G. Ladurner, D. V. Filippov, *Angew. Chem. Int. Ed.* **2016**, *55*, 10634–10638.

[8] E. R. van Rijssel, P. van Delft, G. Lodder, H. S. Overkleeft, G. A. van der Marel, D. V. Filippov, J. D. C. Codée, *Angew. Chem. Int. Ed.* **2014**, *53*, 10381–10385.

[9] N. Hananya, S. K. Daley, J. D. Bagert, T. W. Muir, *J. Am. Chem. Soc.* **2021**, *143*, 10847–10852.

[10] X. Z. Zhao, D. Hymel, T. R. Burke, *Bioorg. Med. Chem. Lett.* **2016**, *26*, 5009–5012.

[11] K. Nakayama, I. Heise, H. Görner, W. Gärtner, *Photochem. Photobiol.* **2011**, *87*, 1031–1035.

NMR spectra

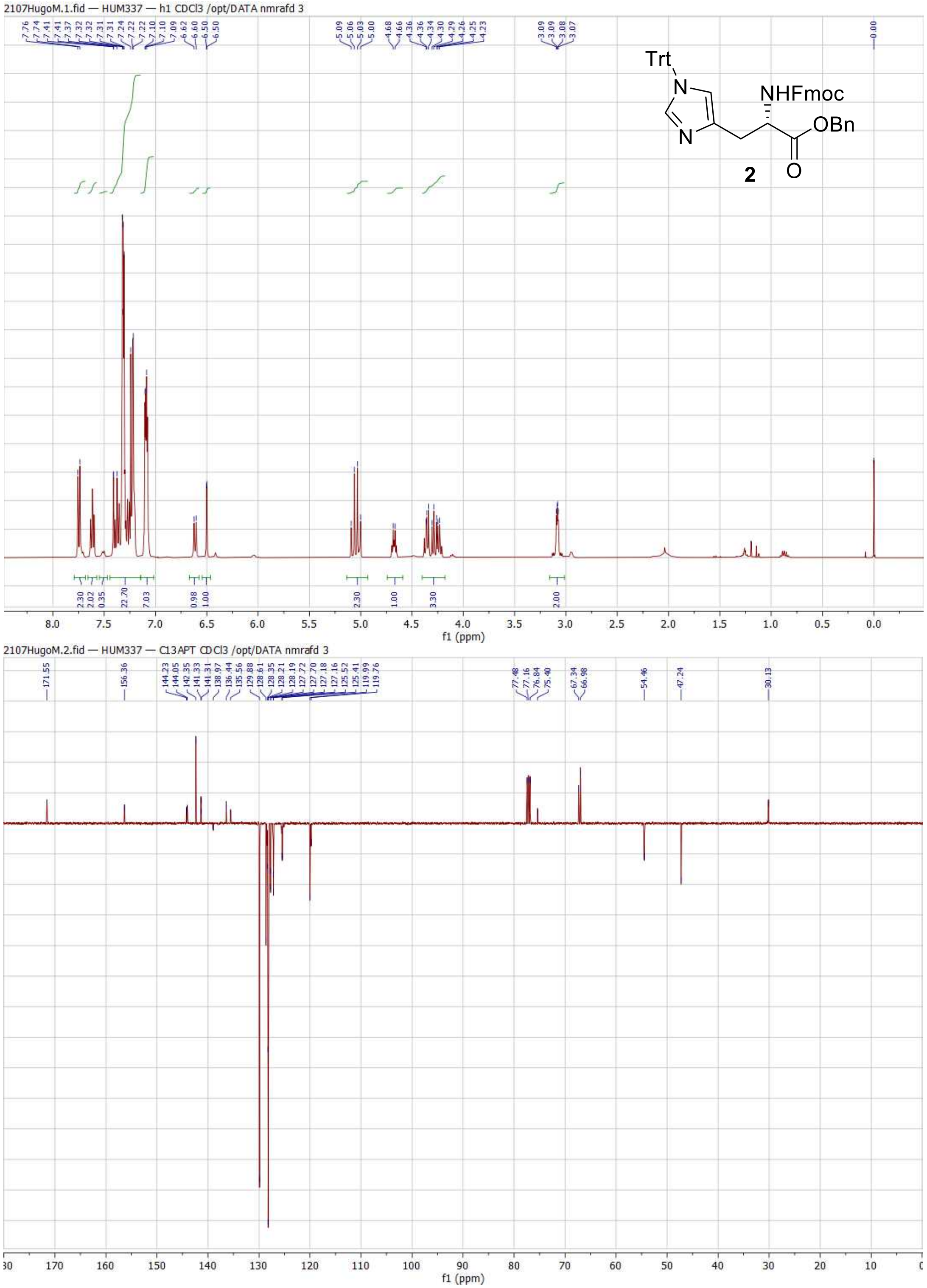

Figure S5: ^{1}HNMR & ^{13}CNMR of compound **2**

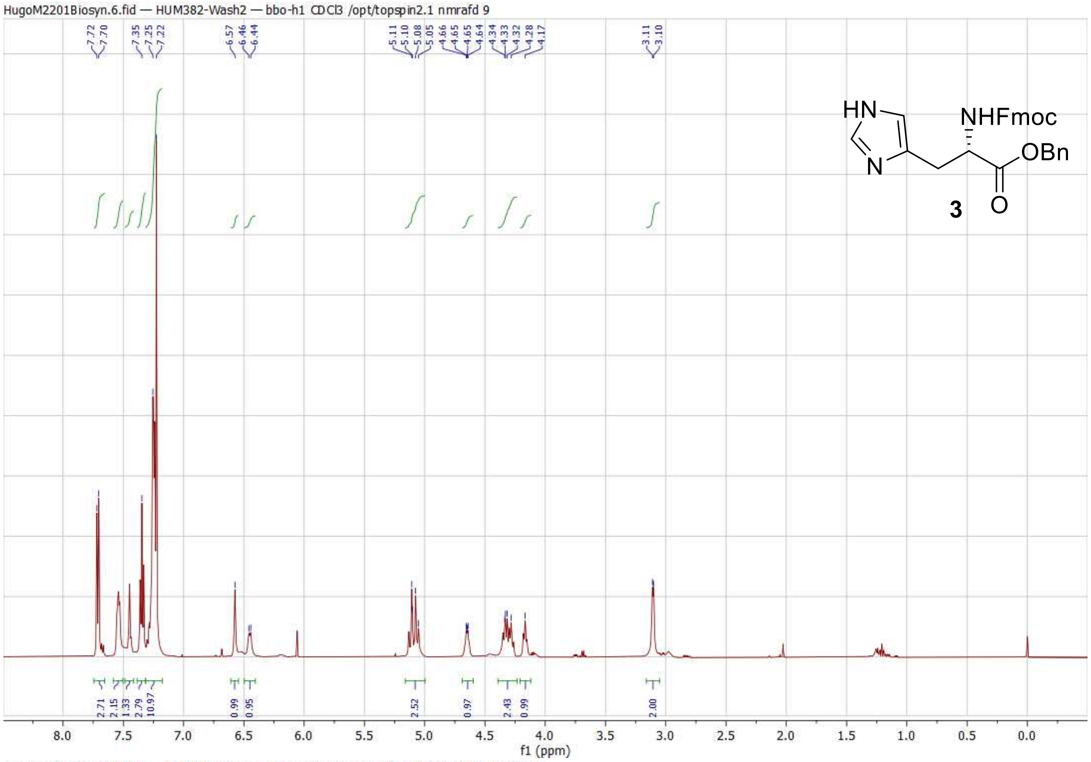

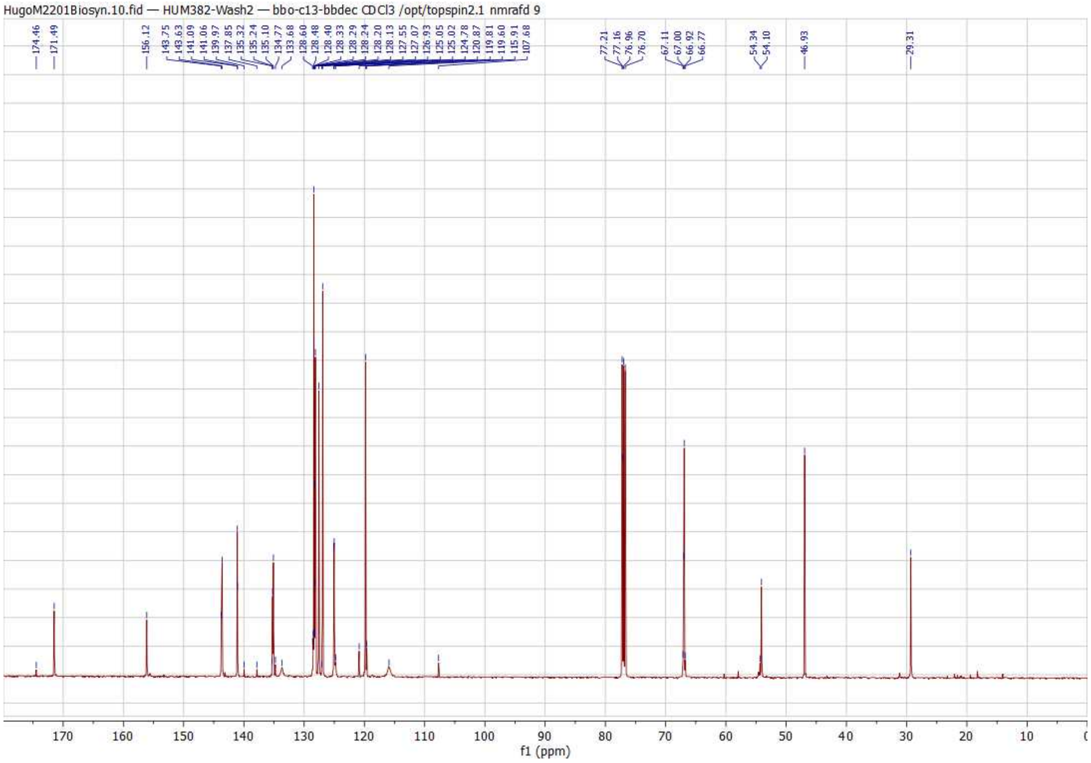

Figure S6: ^{1}HNMR & ^{13}CNMR of compound **3**

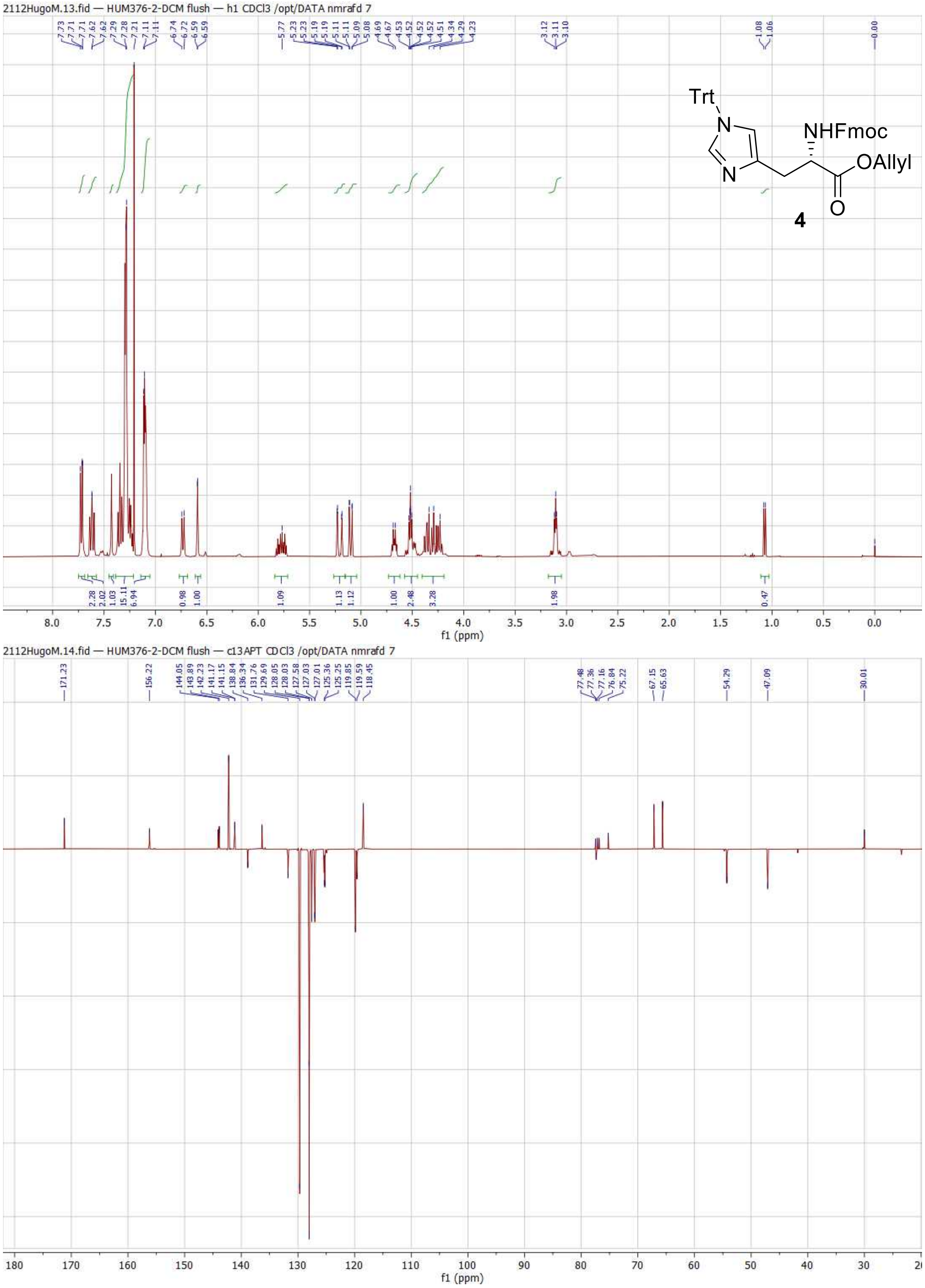

Figure S7: ^{1}HNMR & ^{13}CNMR of compound **4**

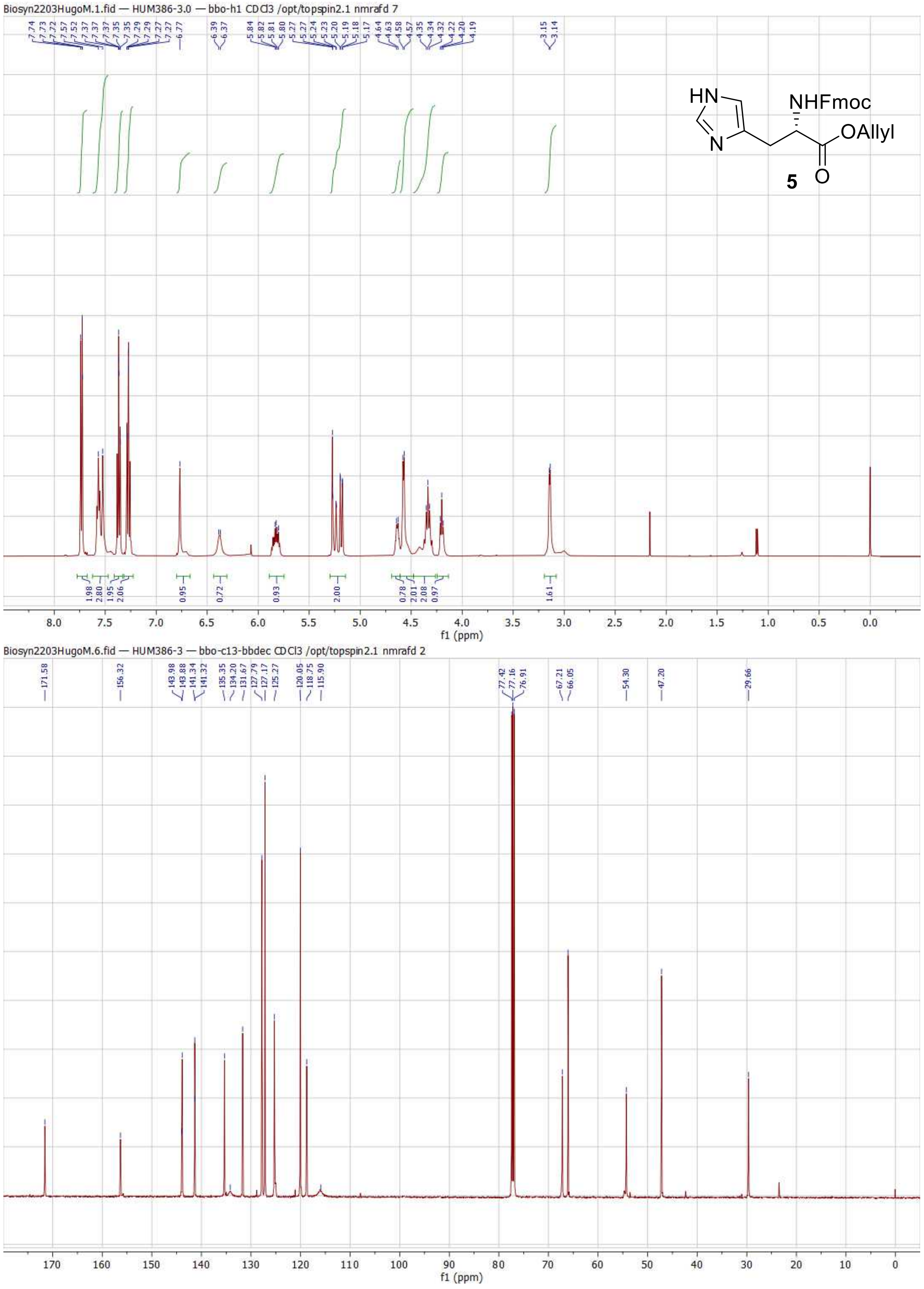

Figure S8: ^{1}HNMR & ^{13}CNMR of compound **5**

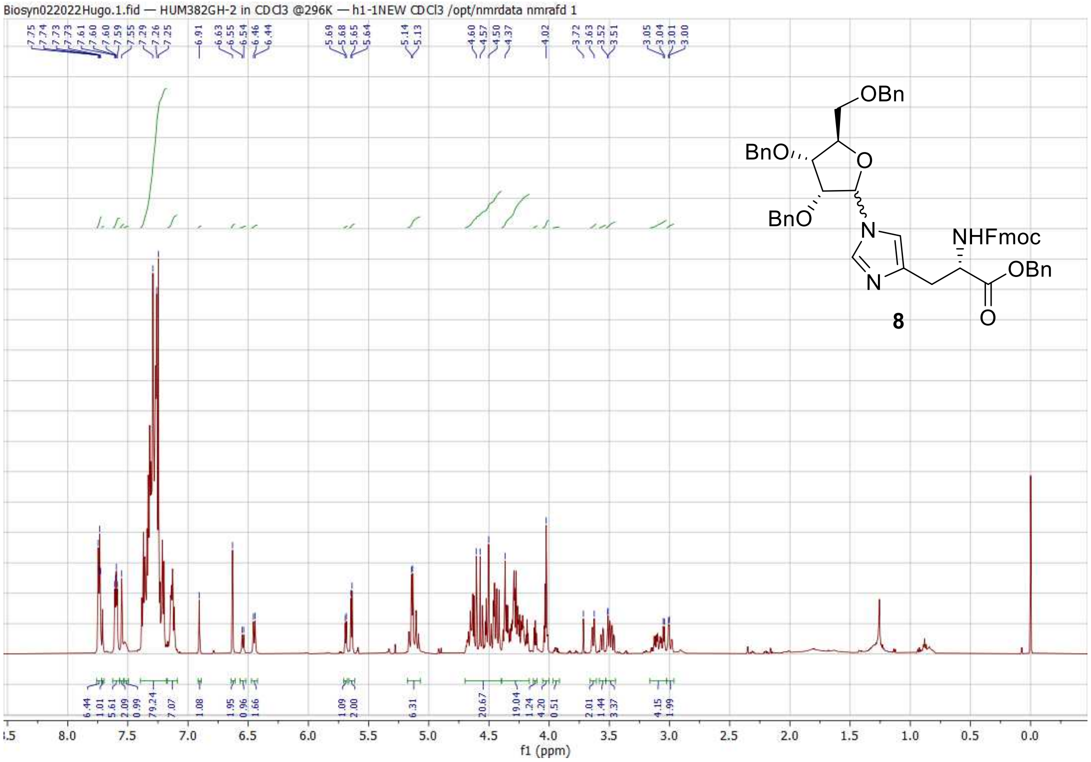

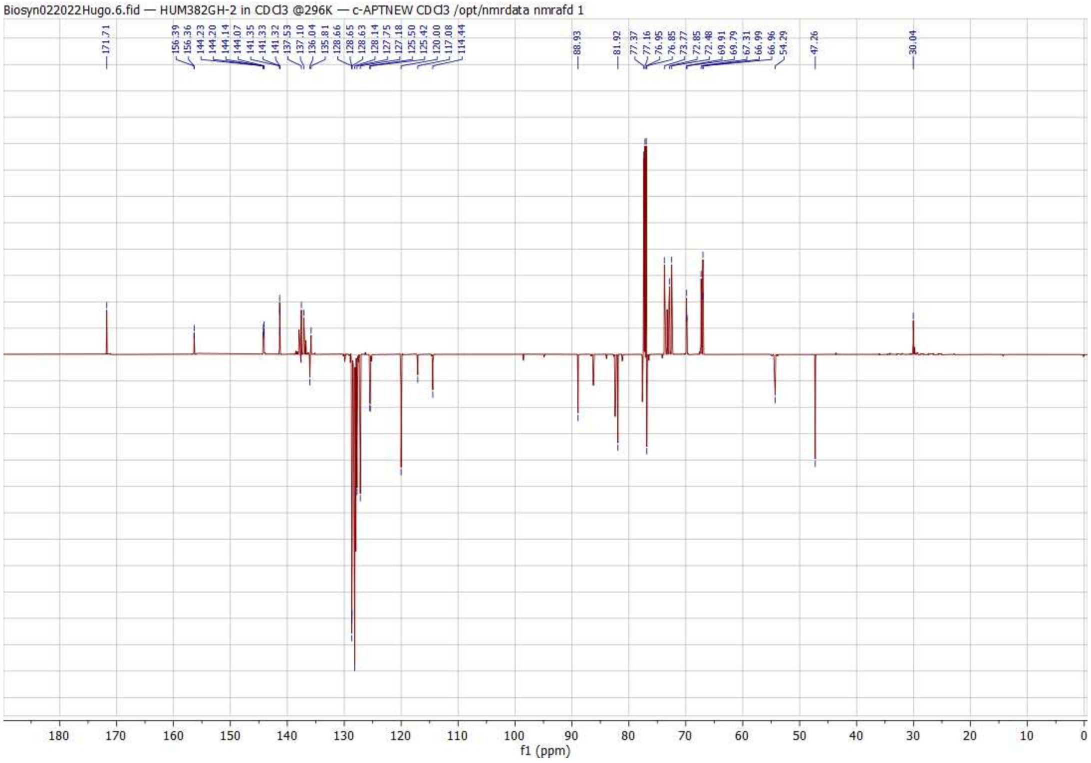

Figure S9: ^{1}HNMR & ^{13}CNMR of compound **8**

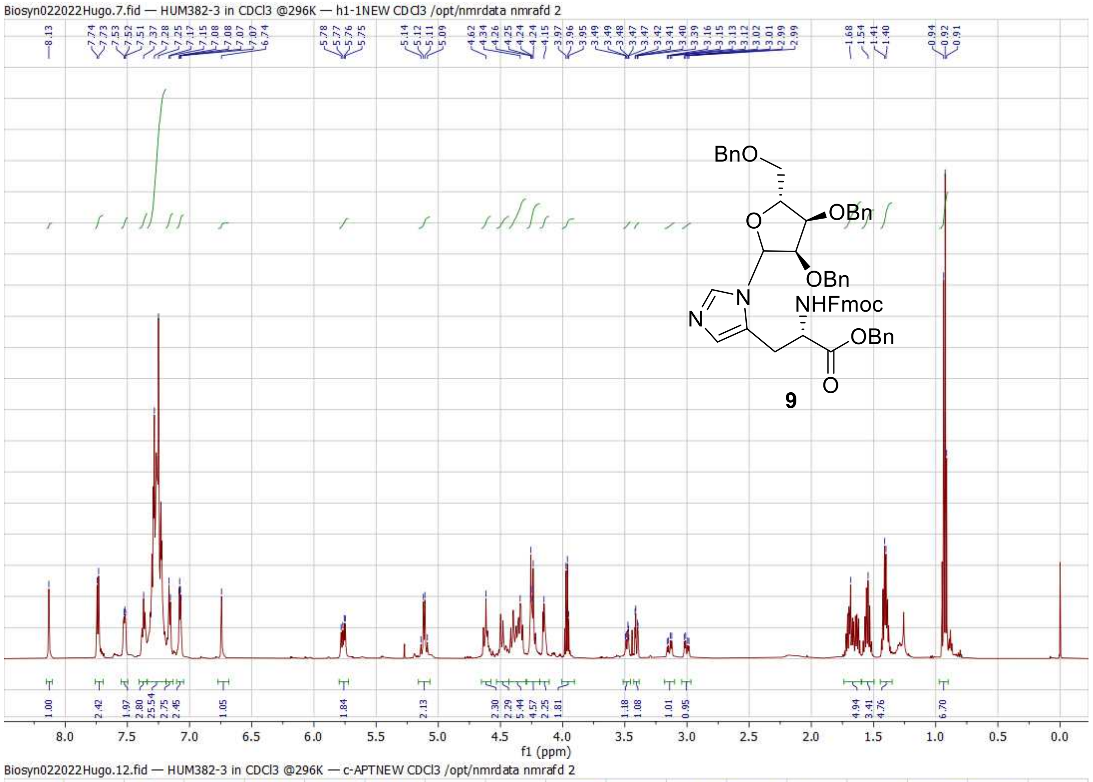

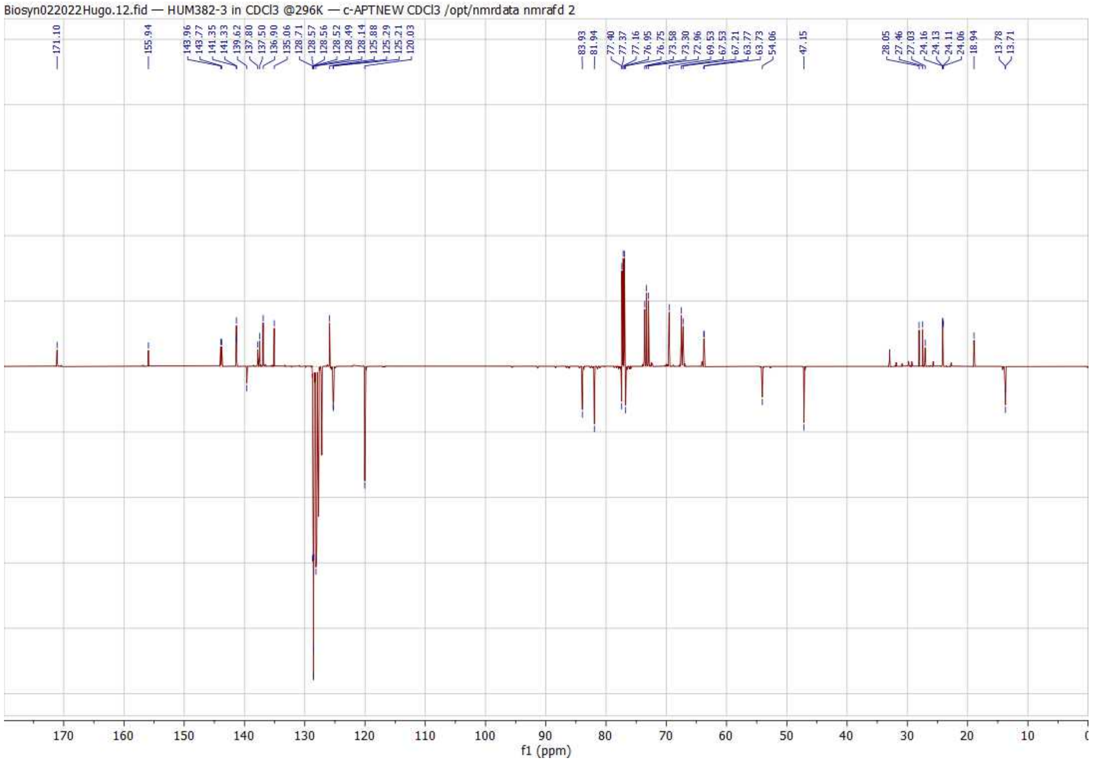

Figure S10: ^{1}HNMR & ^{13}CNMR of compound **9**

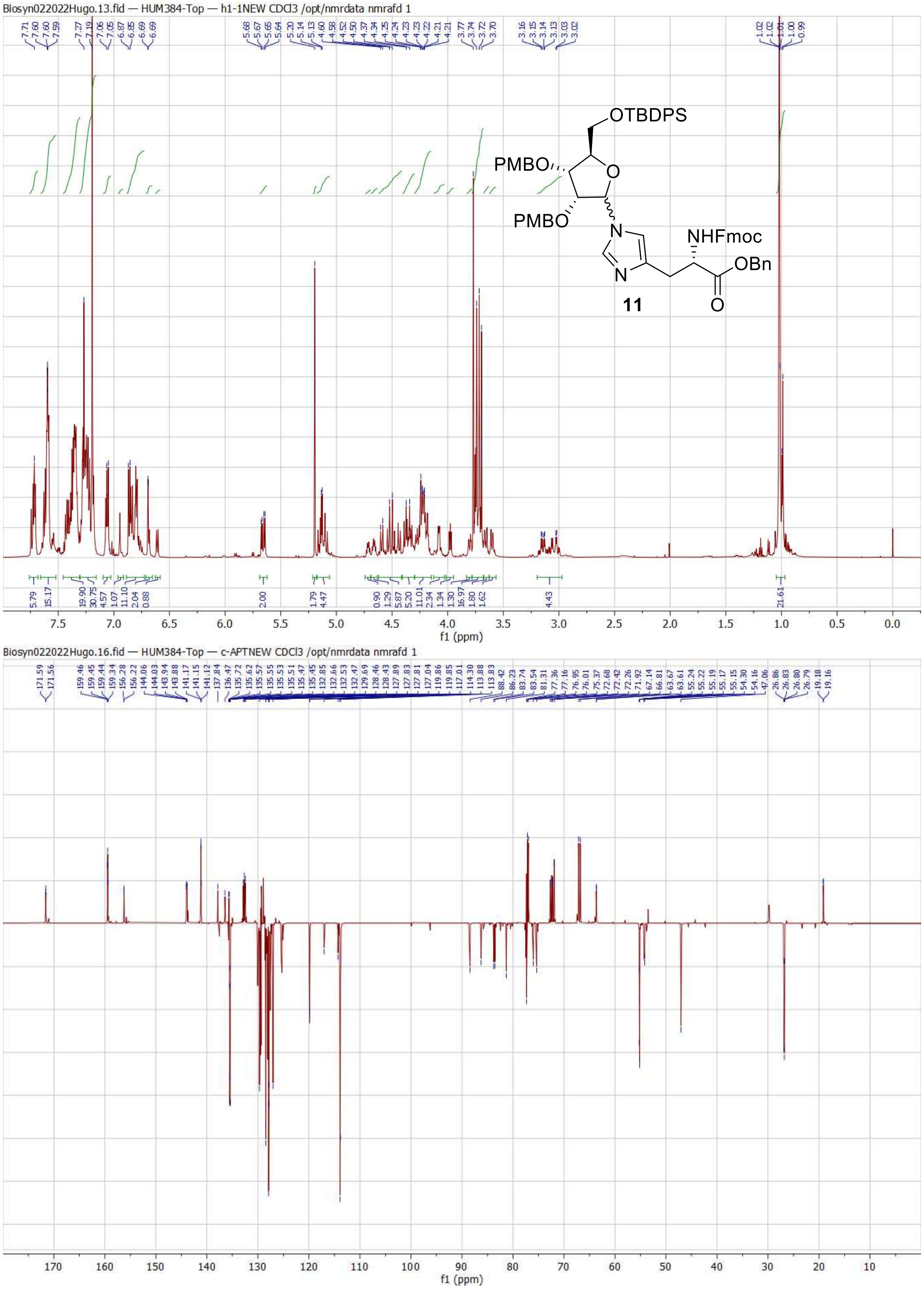

Figure S11: ^{1}HNMR & ^{13}CNMR of compound **11**

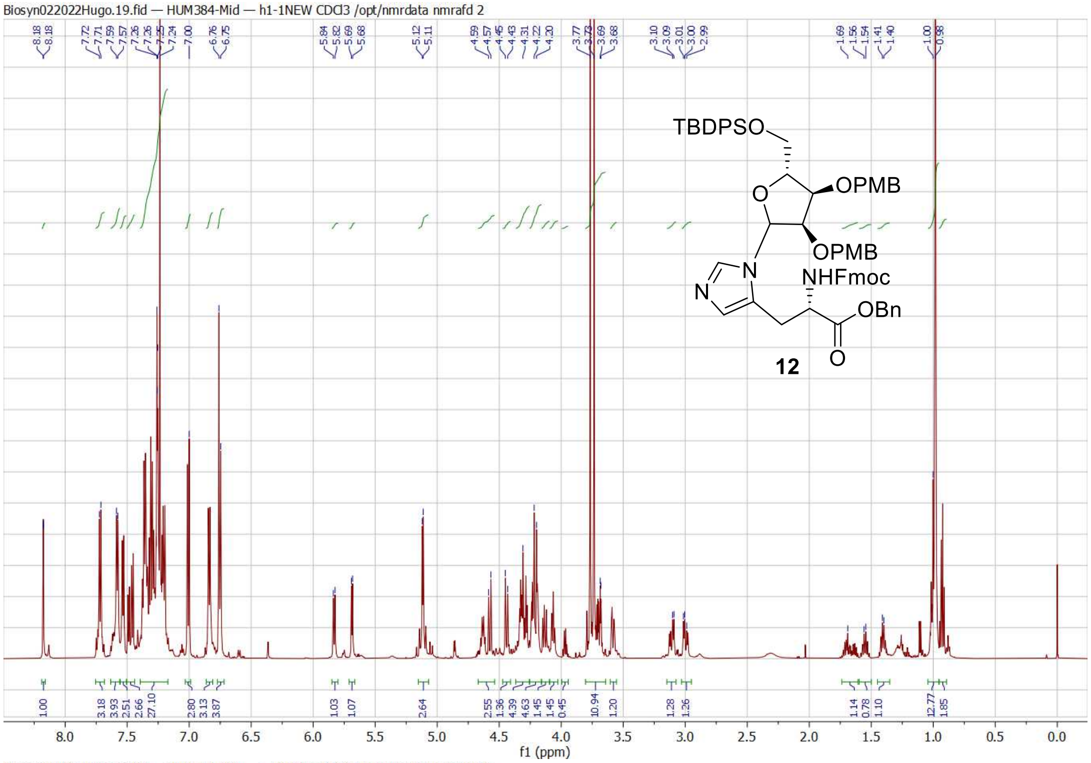

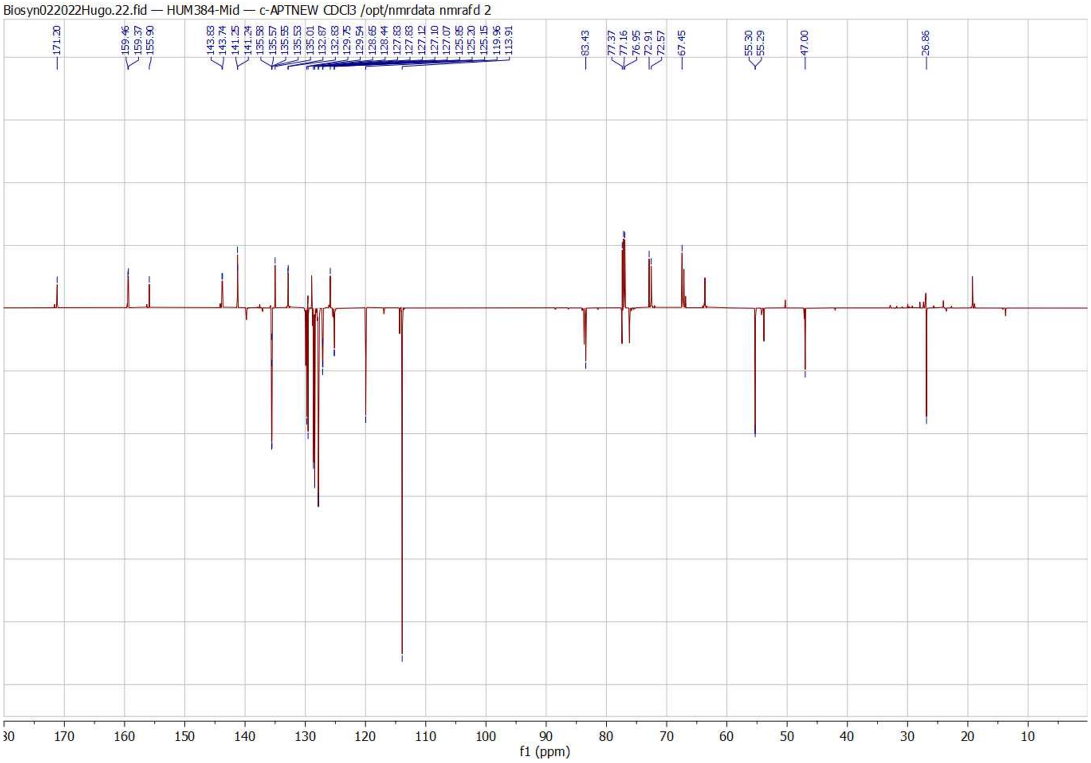

Figure S12: ^{1}HNMR & ^{13}CNMR of compound **12**

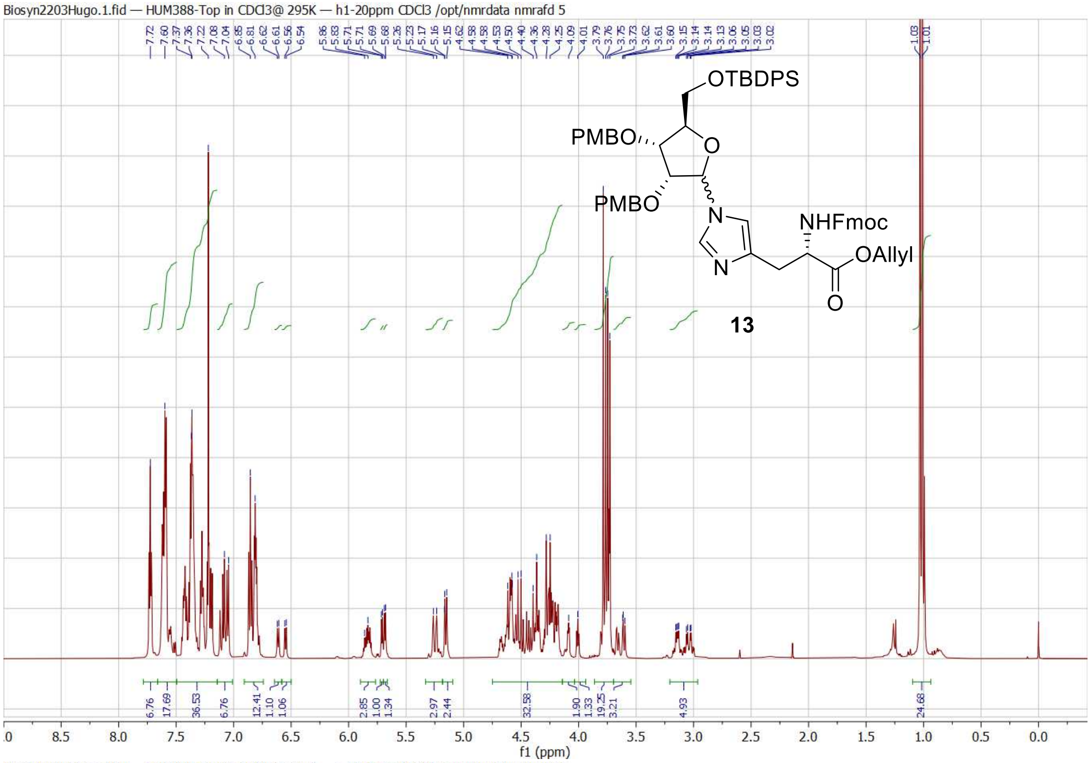
Biosyn2203Hugo.1.fid — HUM388-Top in CDCl3@ 295K — h1-20ppm CDCl3 /opt/nmrdata nmrafd 5
OTBDPS
PMBO
PMBO
O
N
N
NHFmoc
OAllyl
O
13
f1 (ppm)

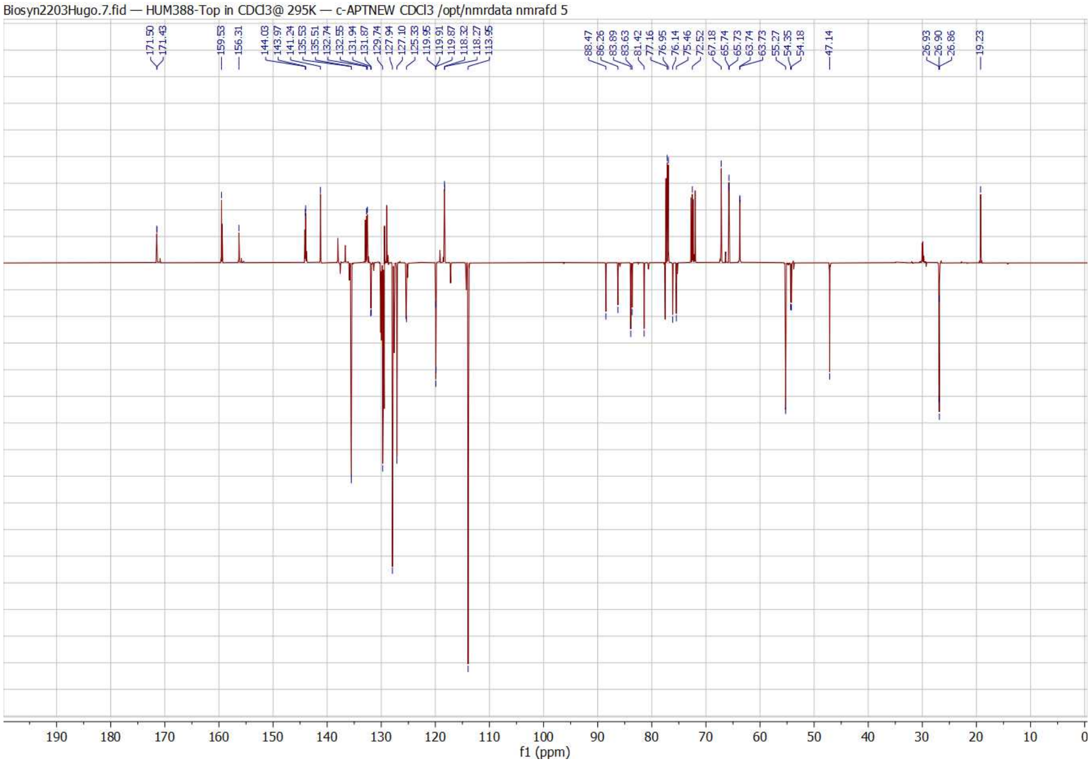
Biosyn2203Hugo.7.fid — HUM388-Top in CDCl3@ 295K — c-APTNEW CDCl3 /opt/nmrdata nmrafd 5
f1 (ppm)

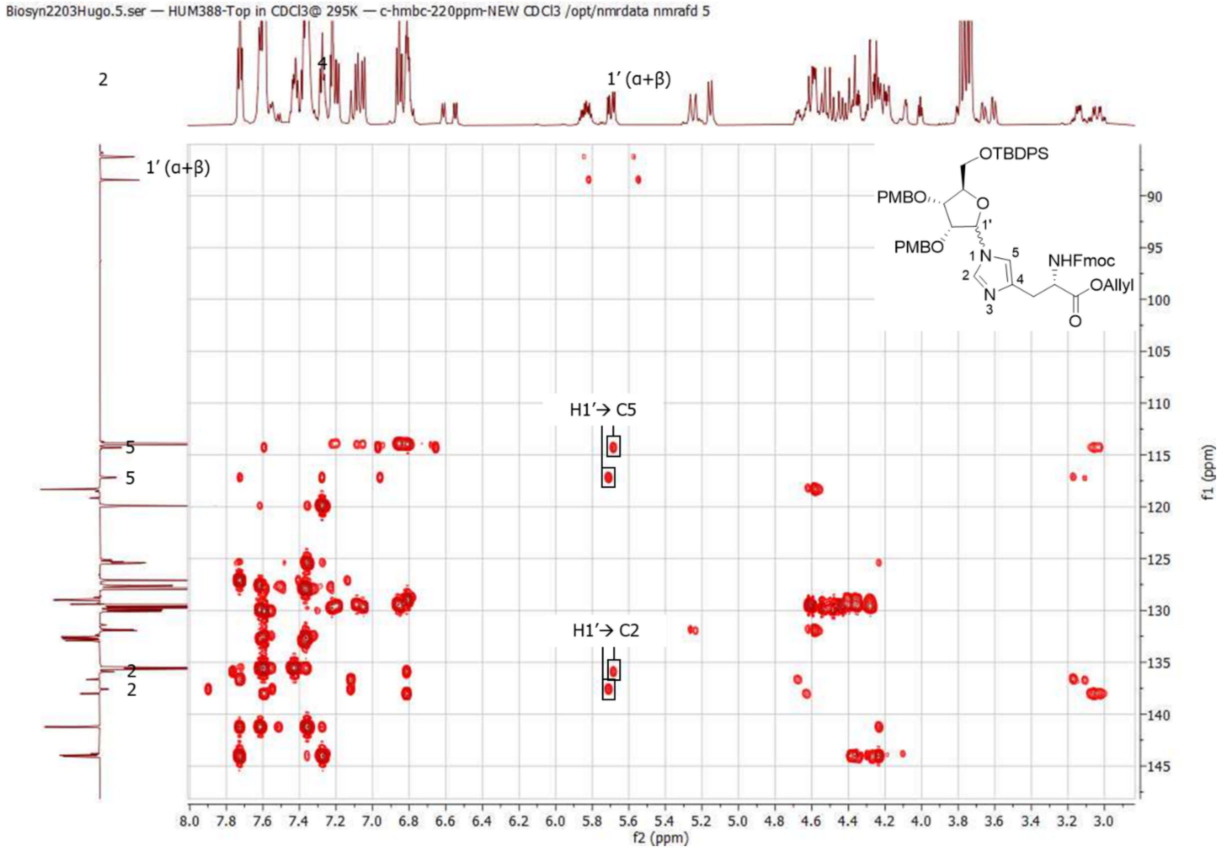

Figure S13: ^{1}HNMR, ^{13}CNMR & HMBC of compound **13**

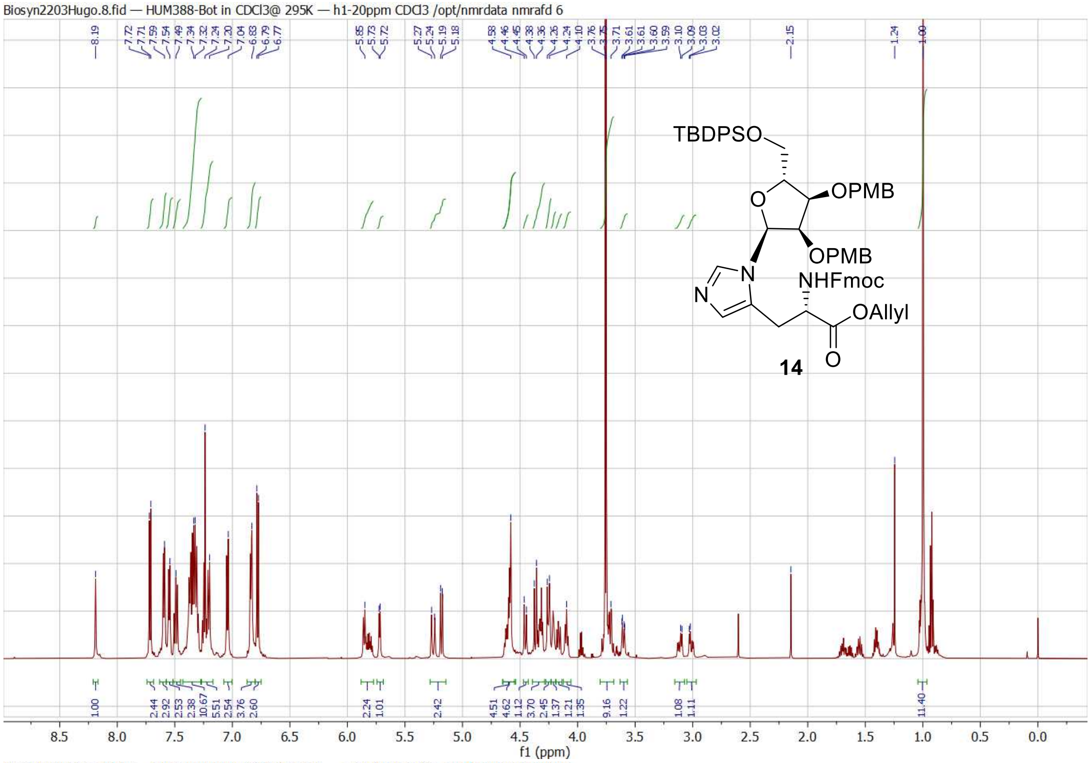
Biosyn2203Hugo.8.fid — HUM388-Bot in CDCl3@ 295K — h1-20ppm CDCl3 /opt/nmrdata nmrafd 6
8.19
7.72 7.71 7.59 7.54 7.49 7.34 7.32 7.24 7.20 7.04 6.83 6.79 6.77
5.85 5.73 5.72
5.27 5.24 5.19 5.18
4.58 4.46 4.45 4.38 4.36 4.36 4.34 4.10 3.76 3.75 3.71 3.61 3.61 3.60 3.59 3.10 3.09 3.03 3.02
2.15
1.24
1.00
TBDPSO
OPMB
OPMB
NHFmoc
OAllyl
14
1.00 2.44 2.92 2.53 2.38 10.67 5.51 2.54 3.76 2.60 2.24 1.01 2.42 4.51 4.62 1.12 3.70 2.45 1.37 1.21 1.35 9.16 1.22 1.08 1.11 11.40
f1 (ppm)

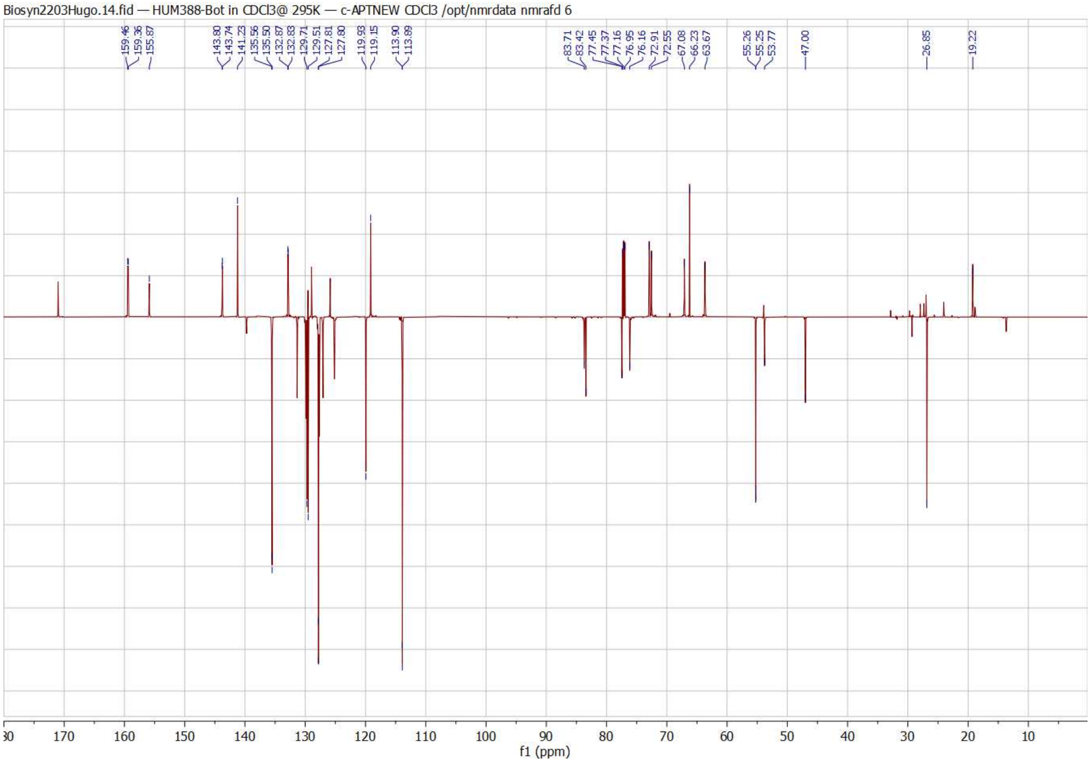
Biosyn2203Hugo.14.fid — HUM388-Bot in CDCl3@ 295K — c-APTNEW CDCl3 /opt/nmrdata nmrafd 6
159.46 159.36 155.87
143.80 143.74 141.23 135.56 135.50 132.87 132.83 129.71 129.51 127.81 127.80 119.93 119.15 113.90 113.89
83.71 83.42 77.45 77.37 77.16 76.95 76.16 72.91 72.55 67.08 66.23 63.67
55.26 55.25 53.77 47.00
26.85 19.22
f1 (ppm)

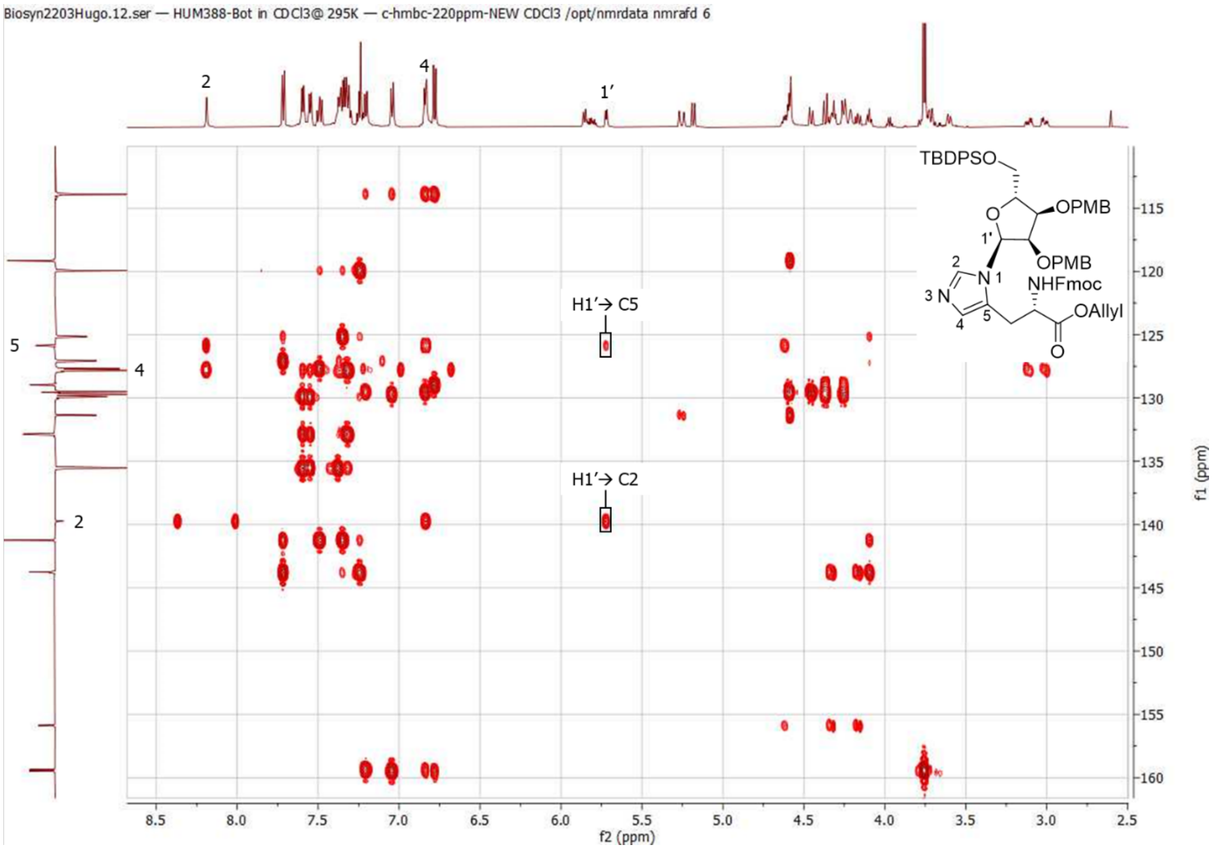

Figure S14: ^{1}HNMR, ^{13}CNMR & HMBC of compound **14**

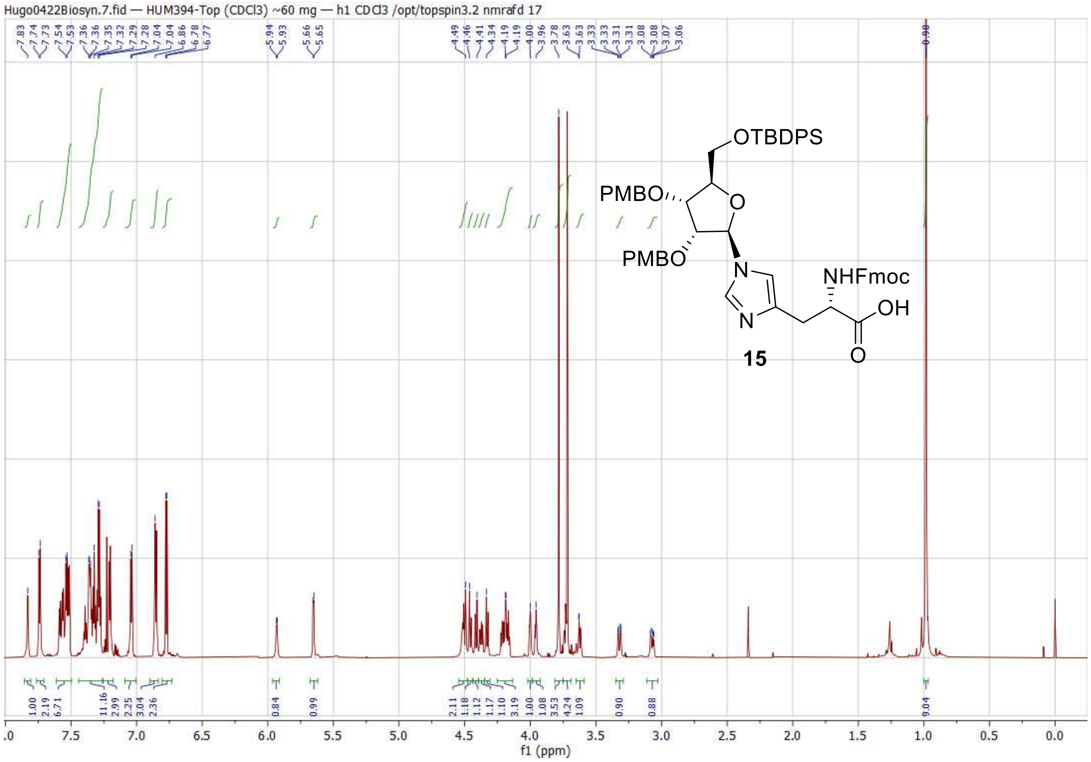
Hugo0422Biosyn.7.fid — HUM394-Top (CDCl3) ~60 mg — h1 CDCl3 /opt/topspin3.2 nmrafd 17
OTBDPS
PMBO
PMBO
NHFmoc
OH
15
f1 (ppm)

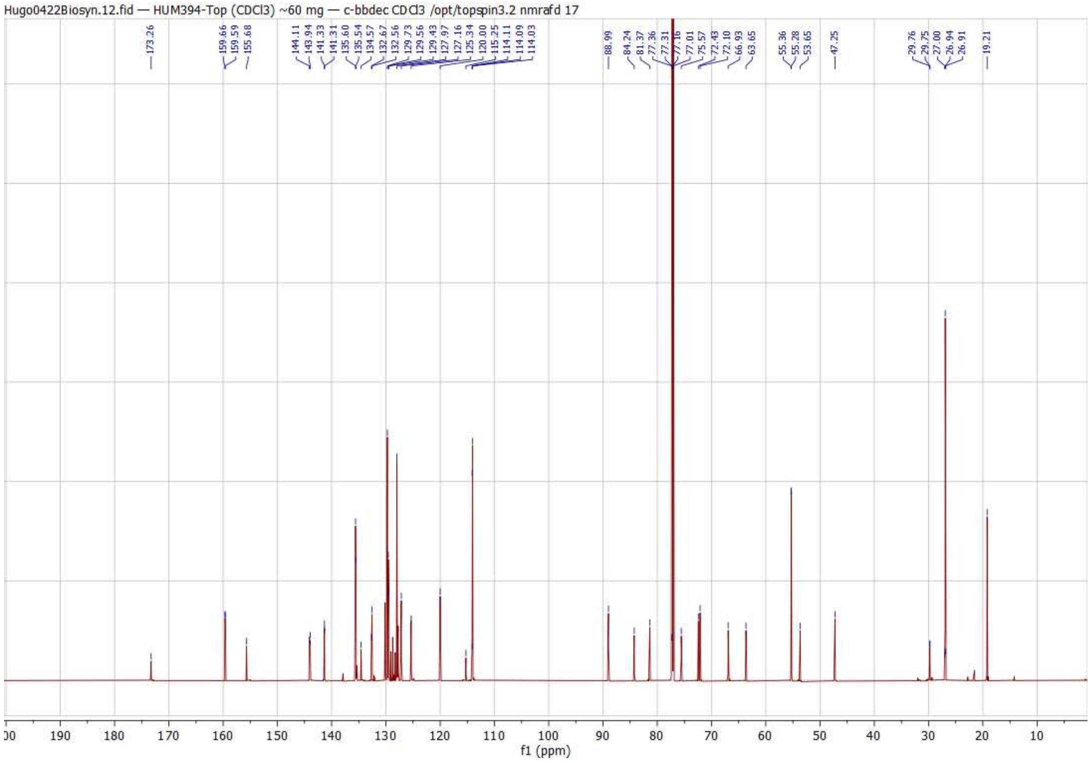
Hugo0422Biosyn.12.fid — HUM394-Top (CDCl3) ~60 mg — c-bbdec CDCl3 /opt/topspin3.2 nmrafd 17
f1 (ppm)

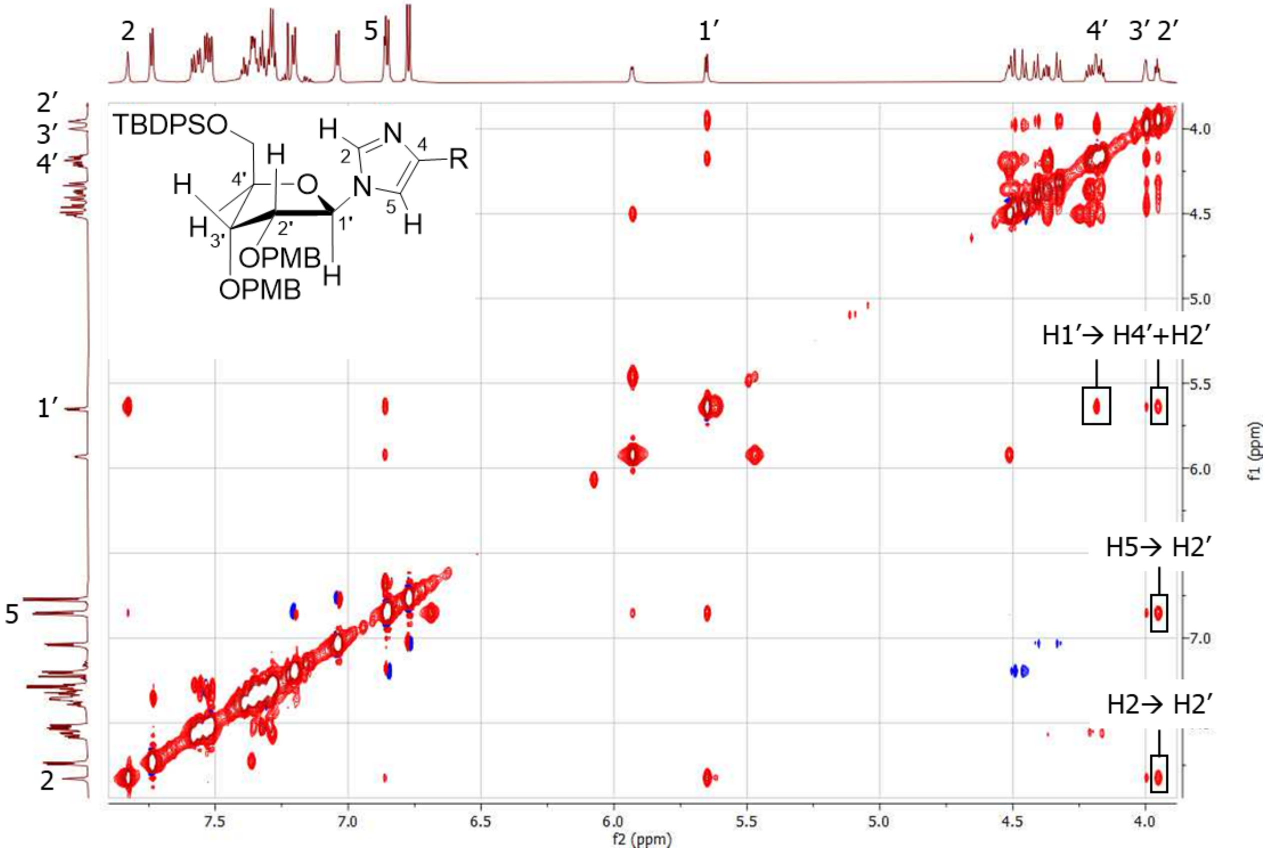

Figure S15: ^{1}HNMR, ^{13}CNMR & NOESY of compound **15**

Hugo0422Biosyn.13.fid — HUM394-Bot (CDCl3) ~60 mg — h1 CDCl3 /opt/topspin3.2 nmrafd 18

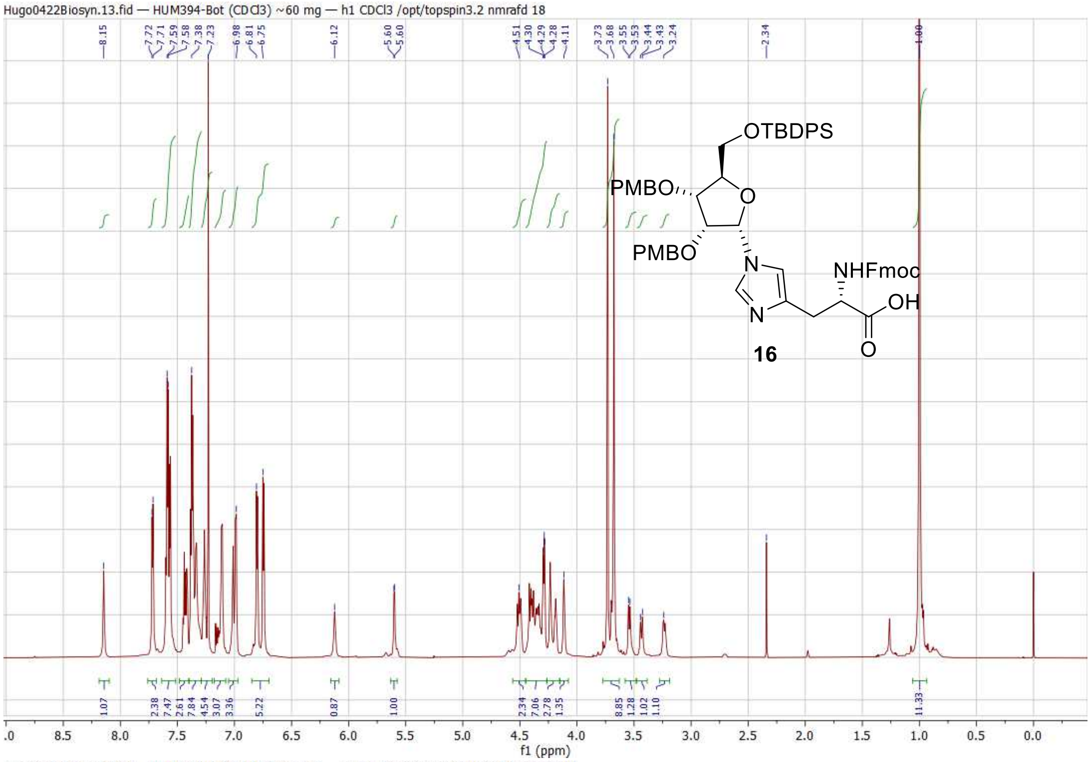

Hugo0422Biosyn.18.fid — HUM394-Bot (CDCl3) ~60 mg — c-bbdec CDCl3 /opt/topspin3.2 nmrafd 18

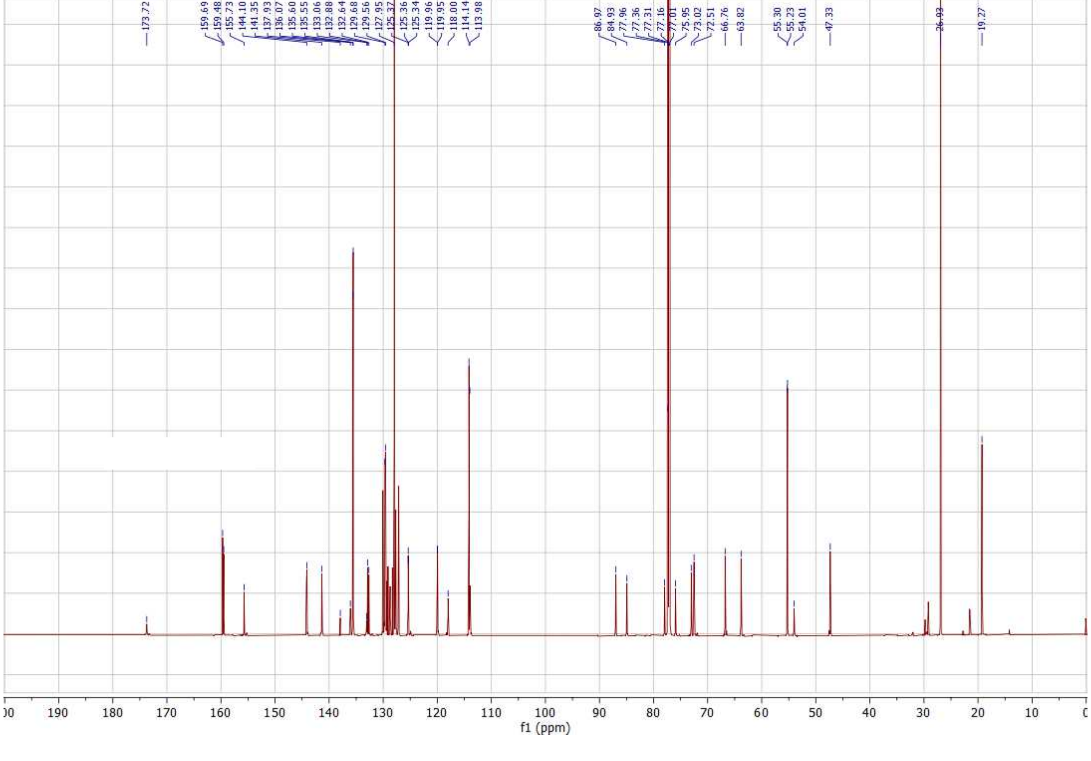

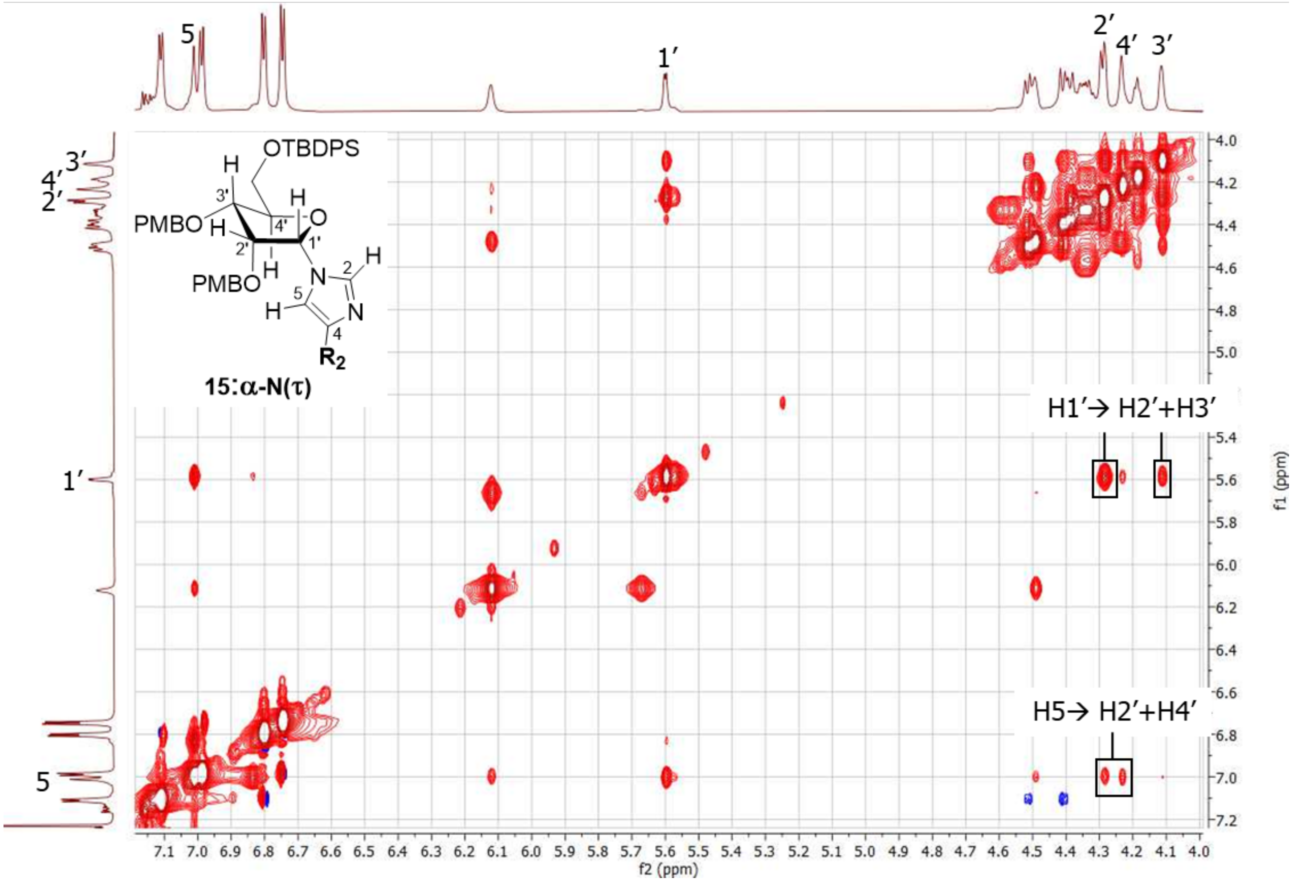

Figure S16: ^{1}HNMR, ^{13}CNMR & NOESY of compound **16**

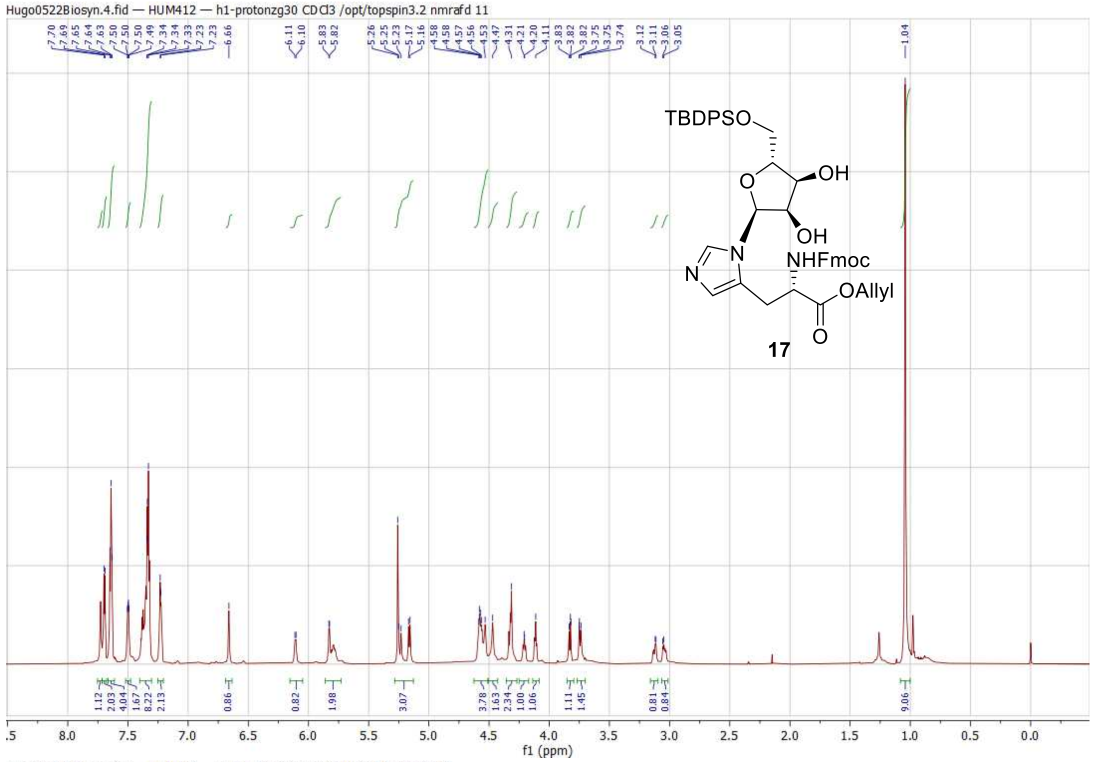
Hugo0522Biosyn.4.fid — HUM412 — h1-protonzg30 CDCl3 /opt/topspin3.2 nmrafd 11
TBDPSO
OH
OH
N
N
NHFmoc
OAllyl
O
17
f1 (ppm)

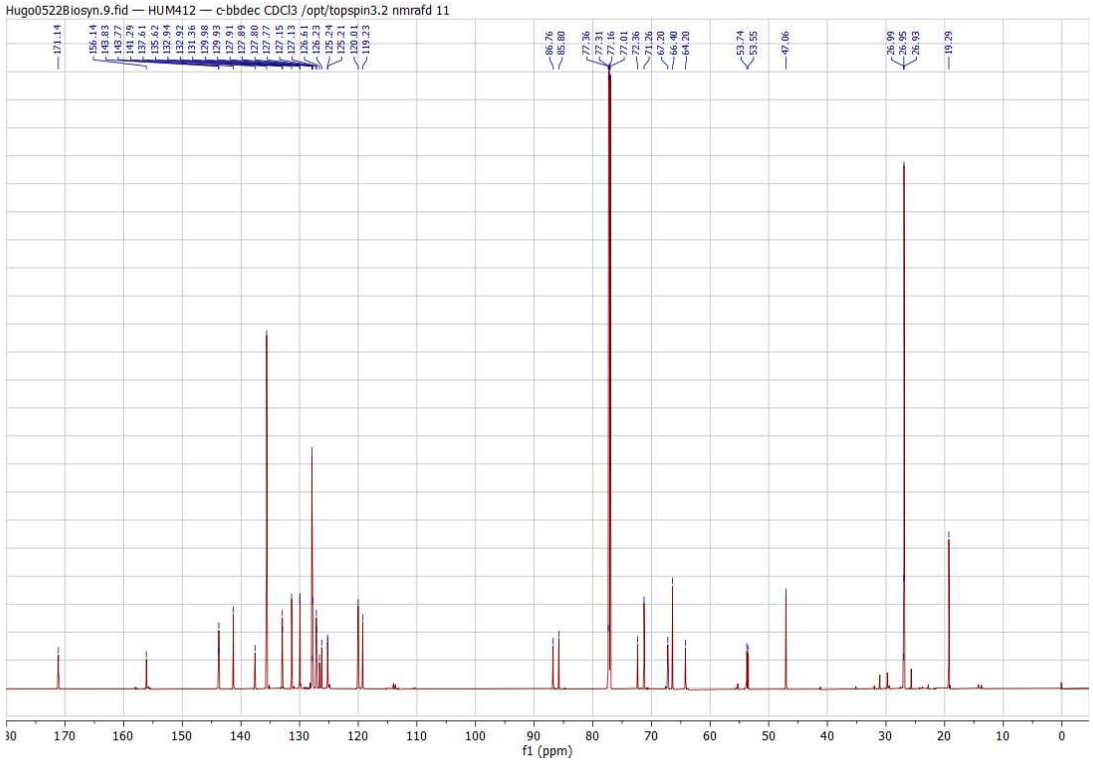
Hugo0522Biosyn.9.fid — HUM412 — c-bbdec CDCl3 /opt/topspin3.2 nmrafd 11
f1 (ppm)

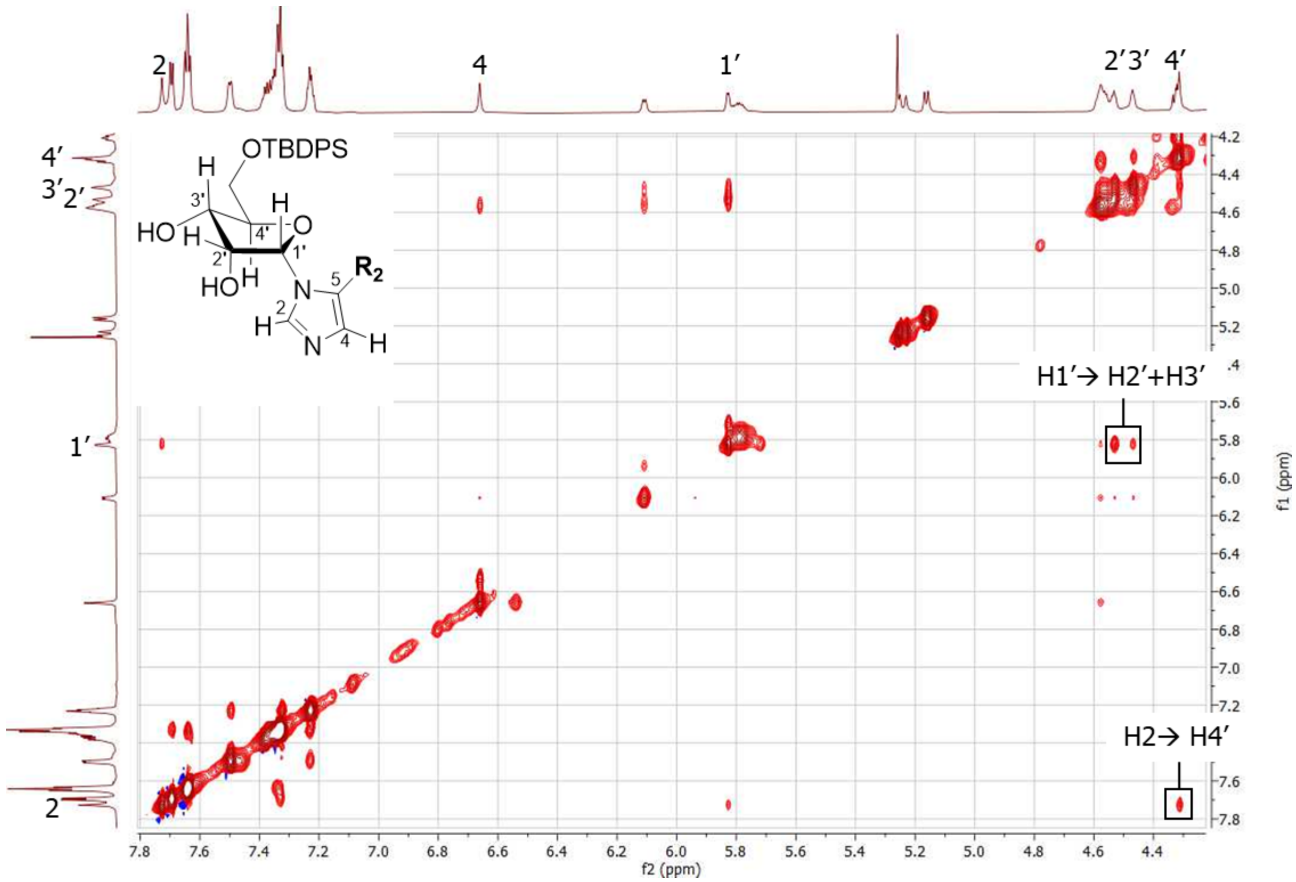

Figure S17: ^{1}HNMR, ^{13}CNMR & NOESY of compound **17**

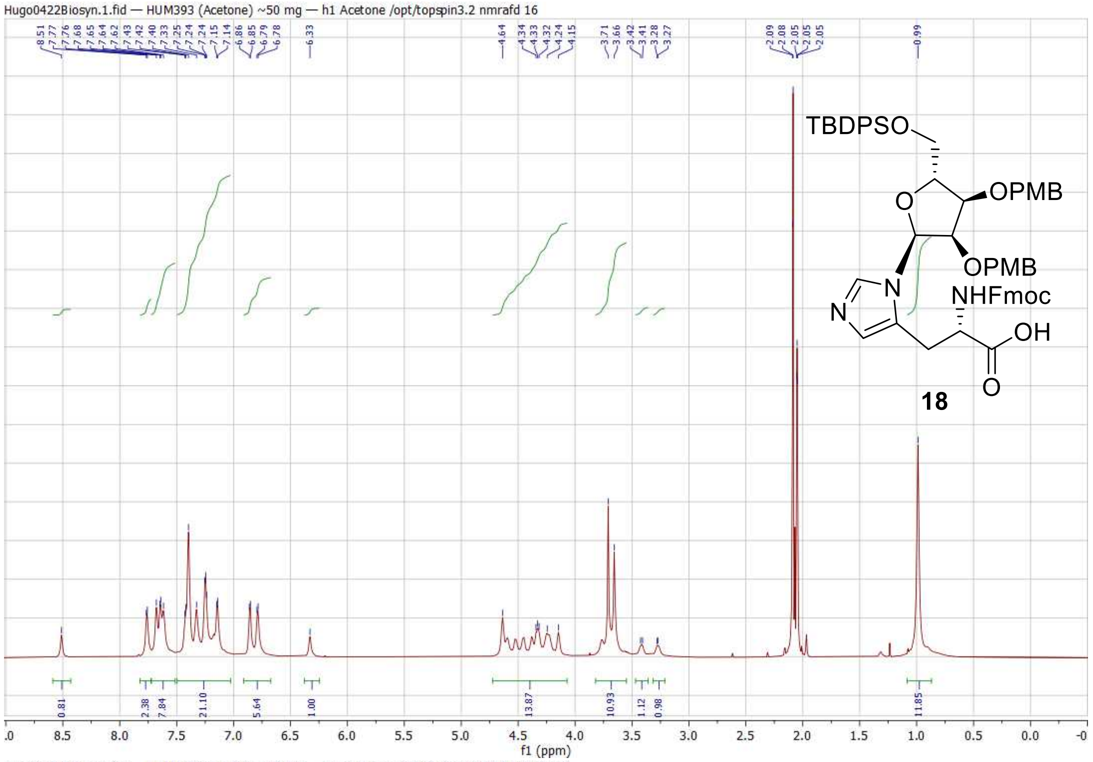

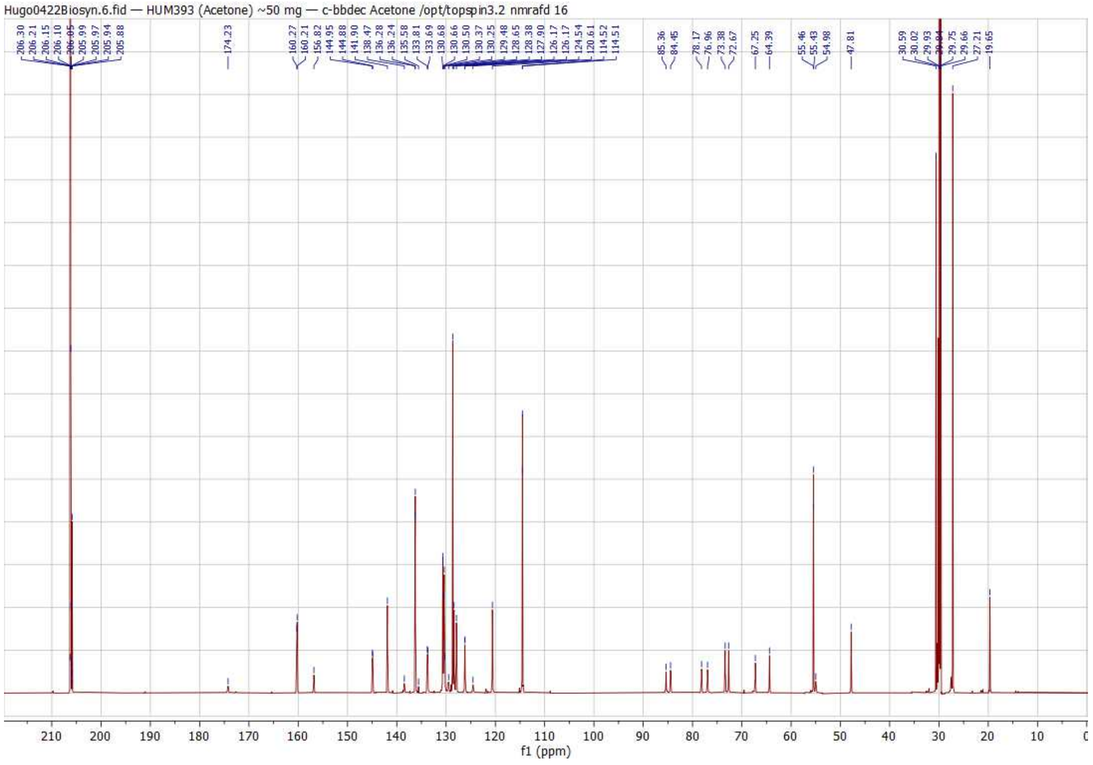

Figure S18: ^{1}HNMR & ^{13}CNMR of compound **18**

HugoM102022.14.fid — 1H NMR av400wb — HUM447-HPLC-FD

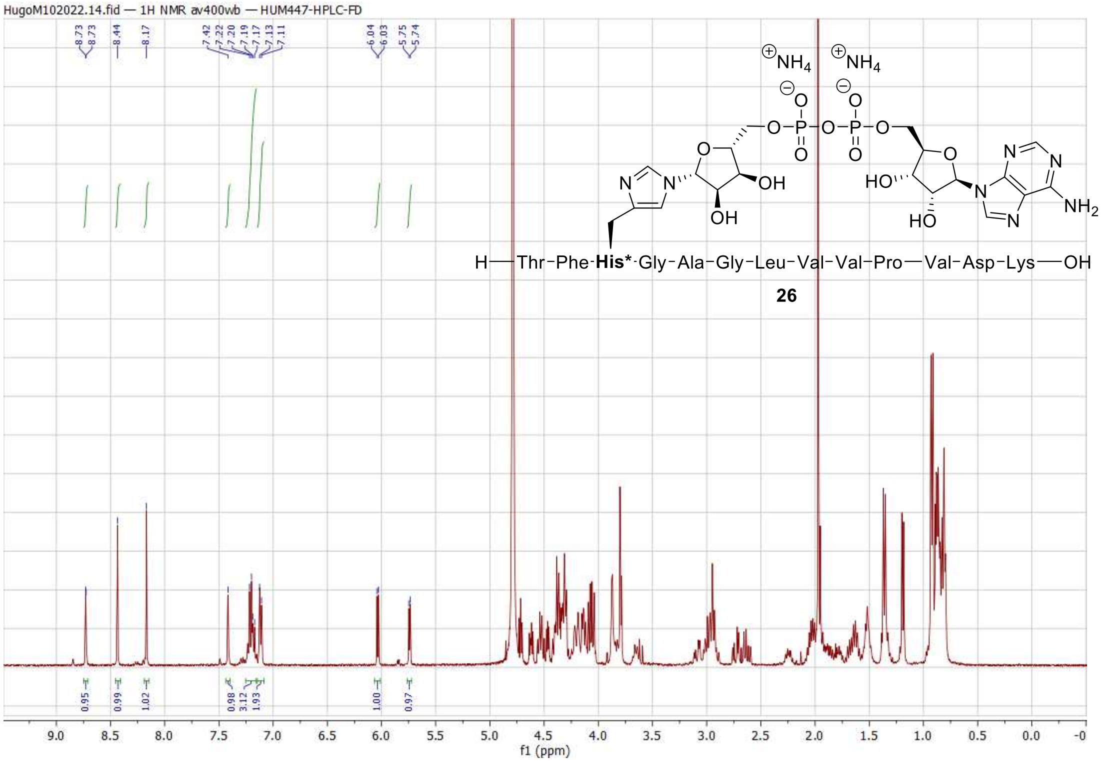

HugoM102022.13.fid — 31P-nmr-1H-dec. av400wb, sw=200_-200 ppm — HUM447-HPLC-FD

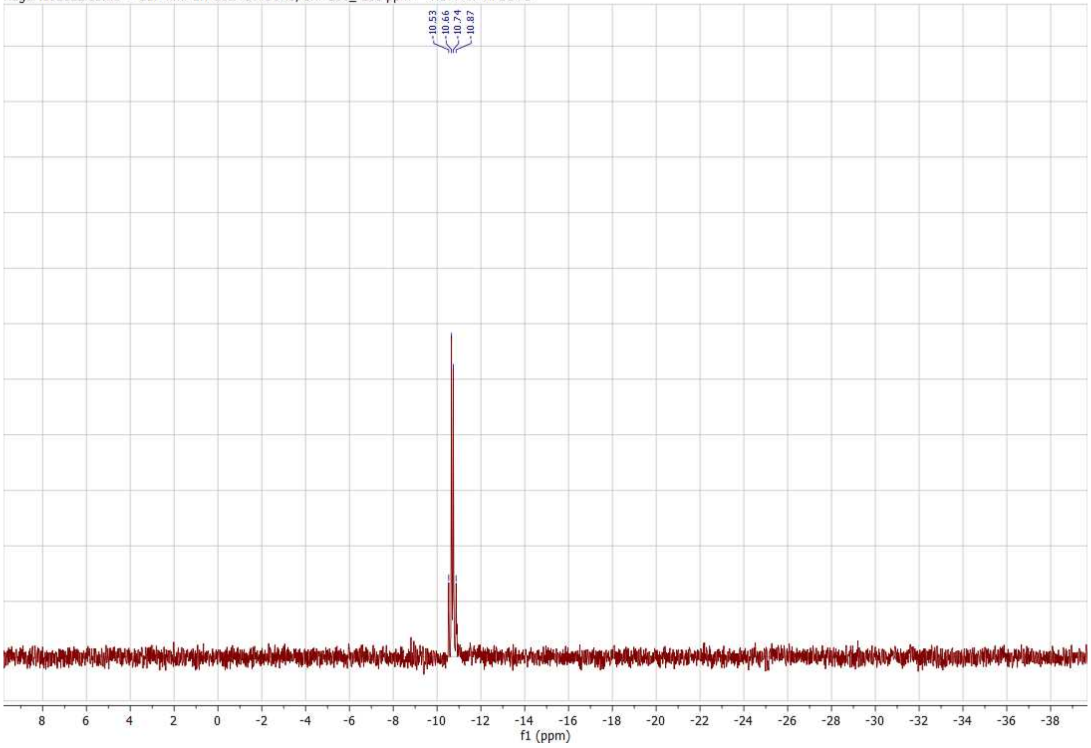

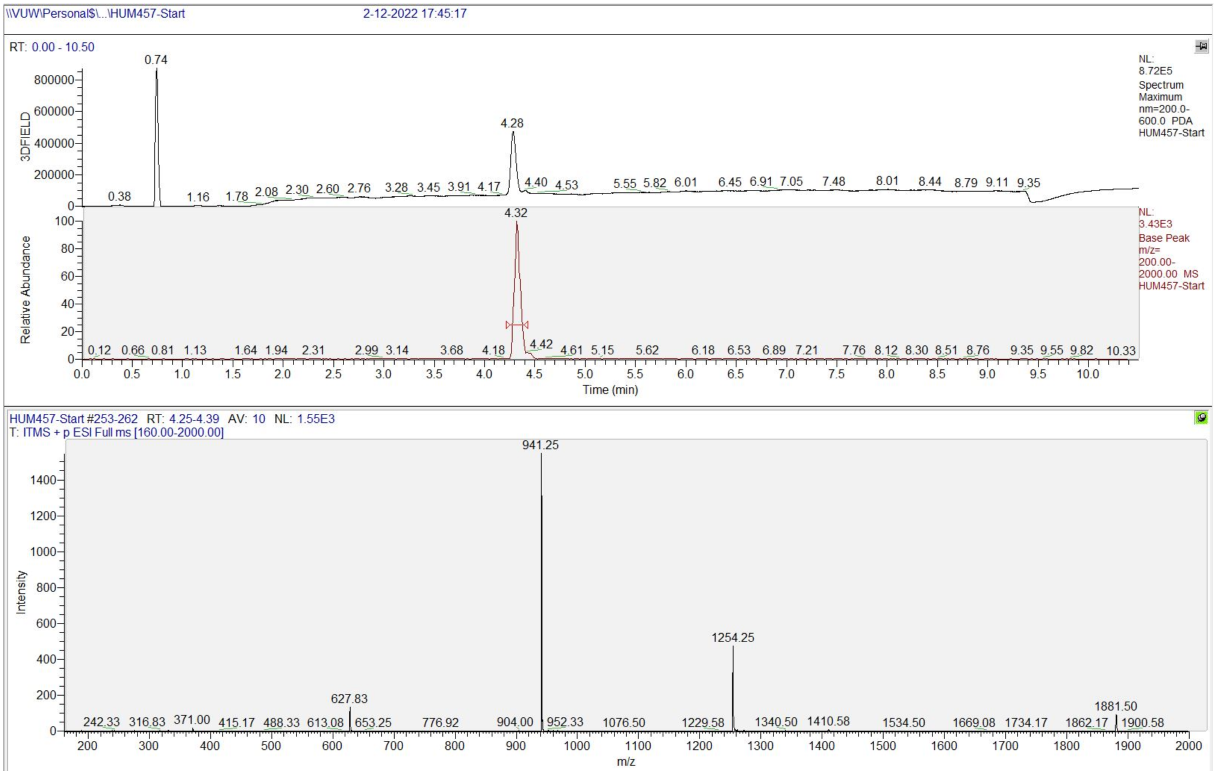

Figure S19: ^{1}HNMR, ^{31}PNMR & LCMS of compound **26**

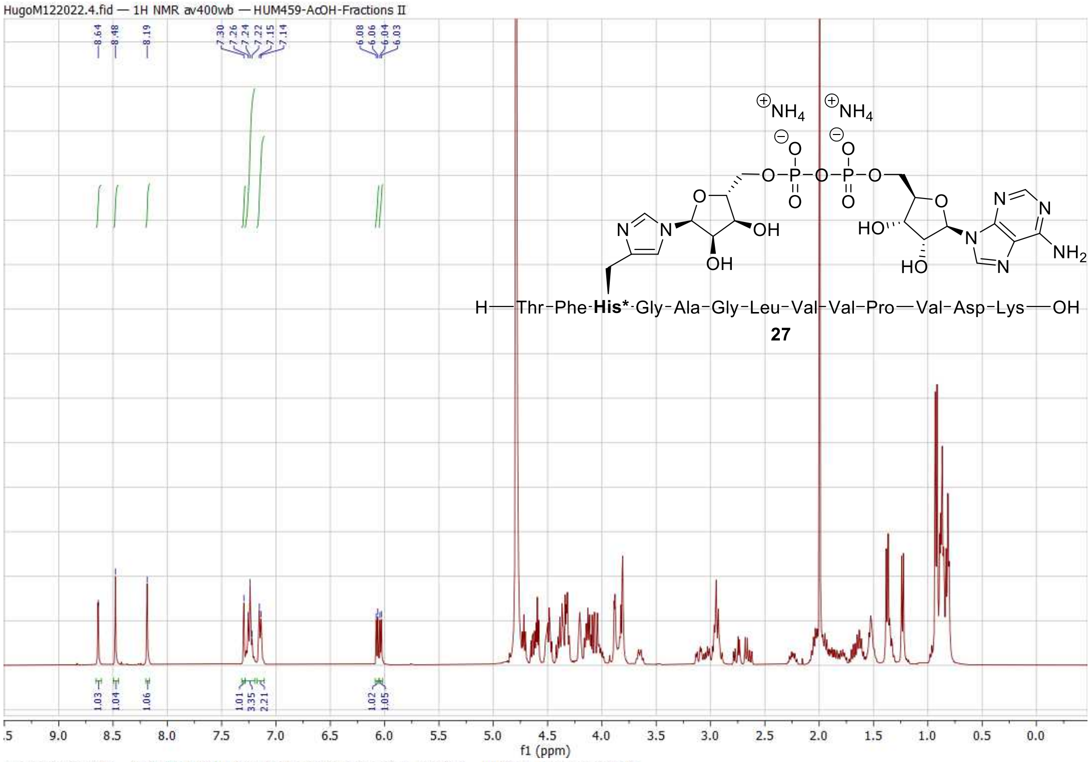
HugoM122022.4.fid — 1H NMR av400wb — HUM459-AcOH-Fractions II
8.64
8.48
8.19
7.30
7.26
7.24
7.22
7.15
7.14
6.08
6.06
6.04
6.03
NH4
NH4
O
O
P
O
P
O
O
O
O
N
N
N
OH
OH
HO
HO
N
N
N
N
NH2
H—Thr-Phe-His*-Gly-Ala-Gly-Leu-Val-Val-Pro—Val-Asp-Lys—OH
27
1.03
1.04
1.06
1.01
3.35
2.21
1.02
1.05
f1 (ppm)

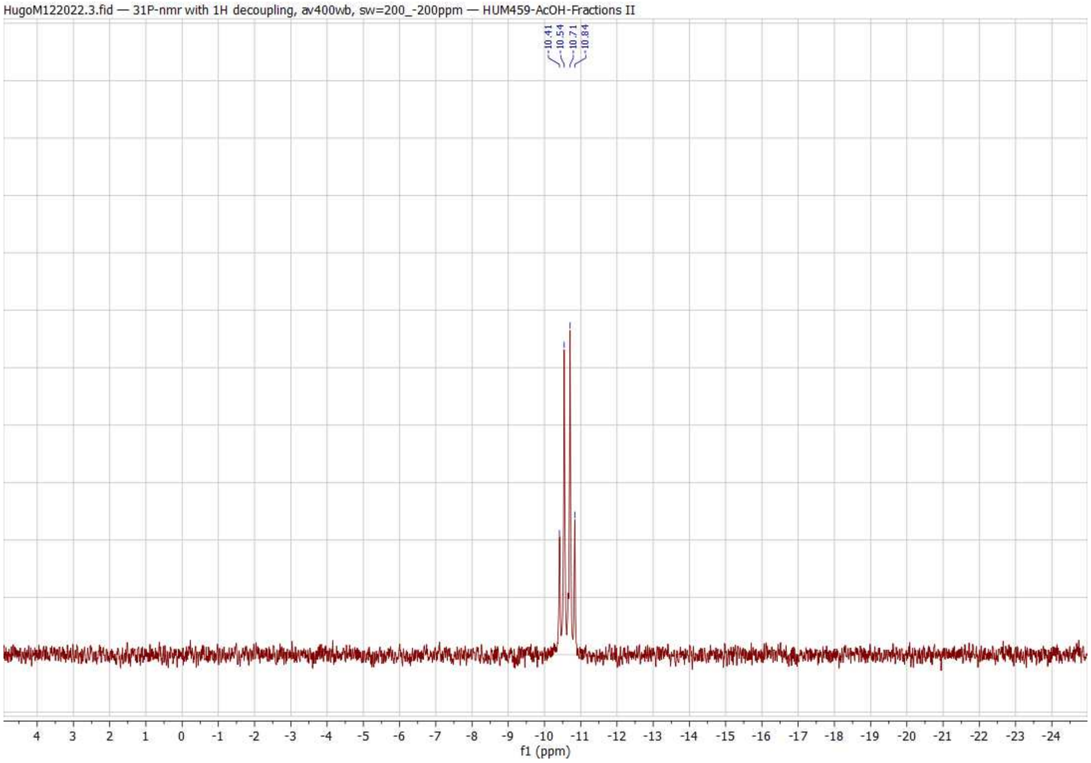
HugoM122022.3.fid — 31P-nmr with 1H decoupling, av400wb, sw=200_-200ppm — HUM459-AcOH-Fractions II
-10.41
-10.54
-10.71
-10.84
f1 (ppm)

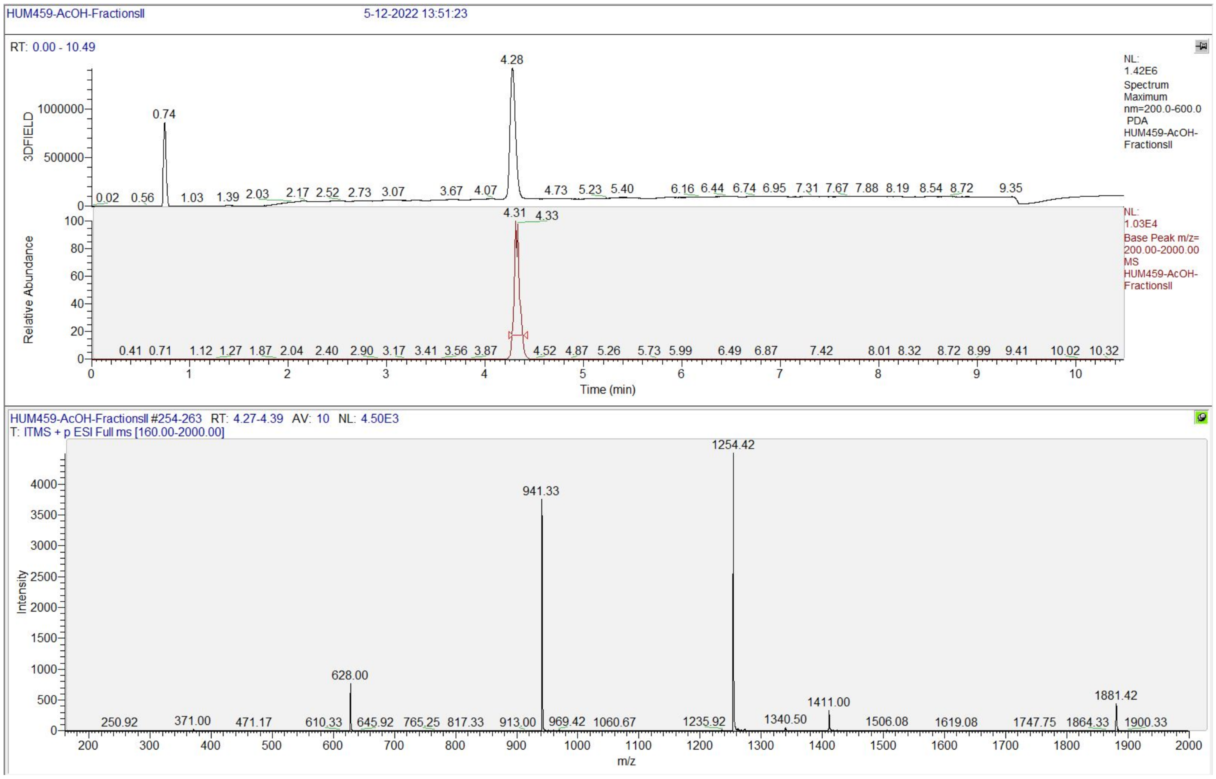

Figure S20: ^{1}HNMR, ^{31}PNMR & LCMS of compound **27**

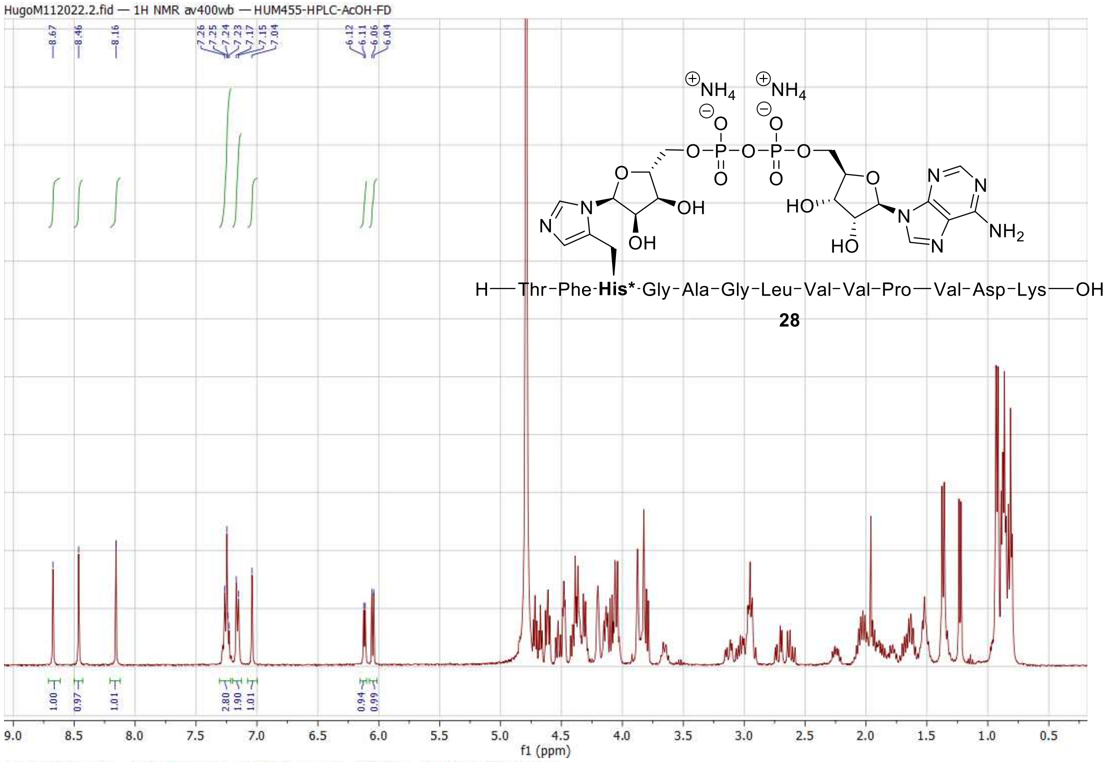
HugoM112022.2.fid — 1H NMR av400wb — HUM455-HPLC-AcOH-FD
H—Thr-Phe-His*-Gly-Ala-Gly-Leu-Val-Val-Pro—Val-Asp-Lys—OH
28
f1 (ppm)

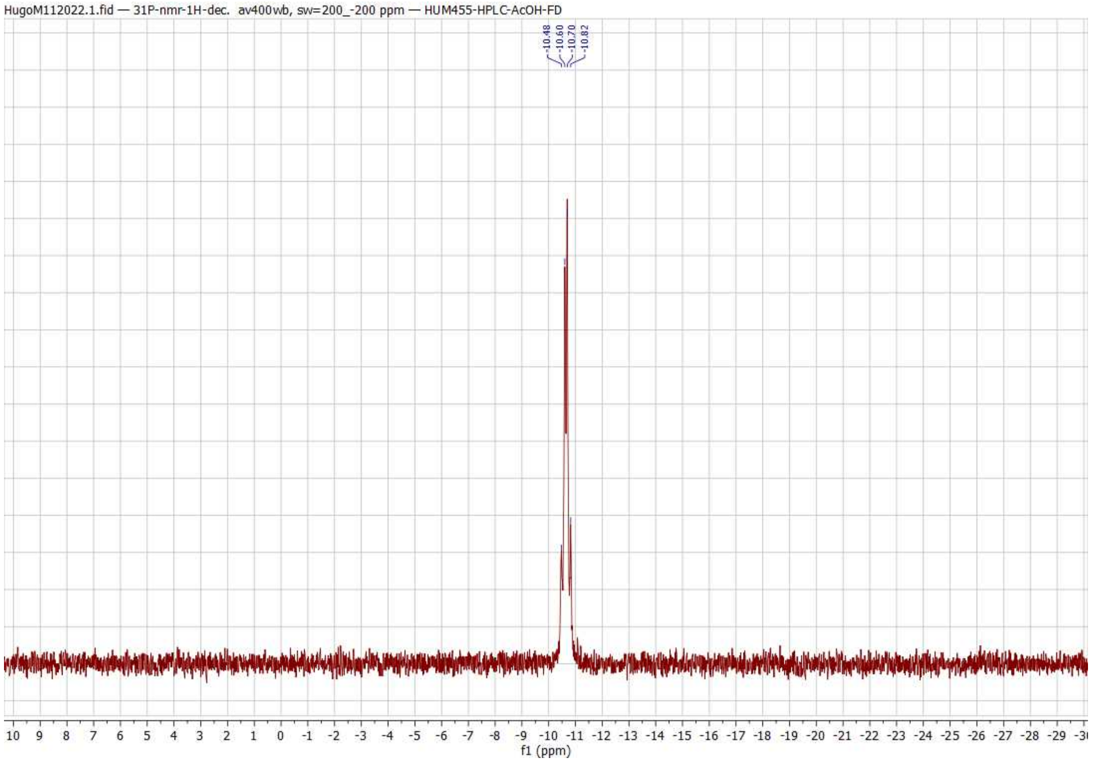
HugoM112022.1.fid — 31P-nmr-1H-dec. av400wb, sw=200_-200 ppm — HUM455-HPLC-AcOH-FD
-10.48
-10.60
-10.70
-10.82
f1 (ppm)

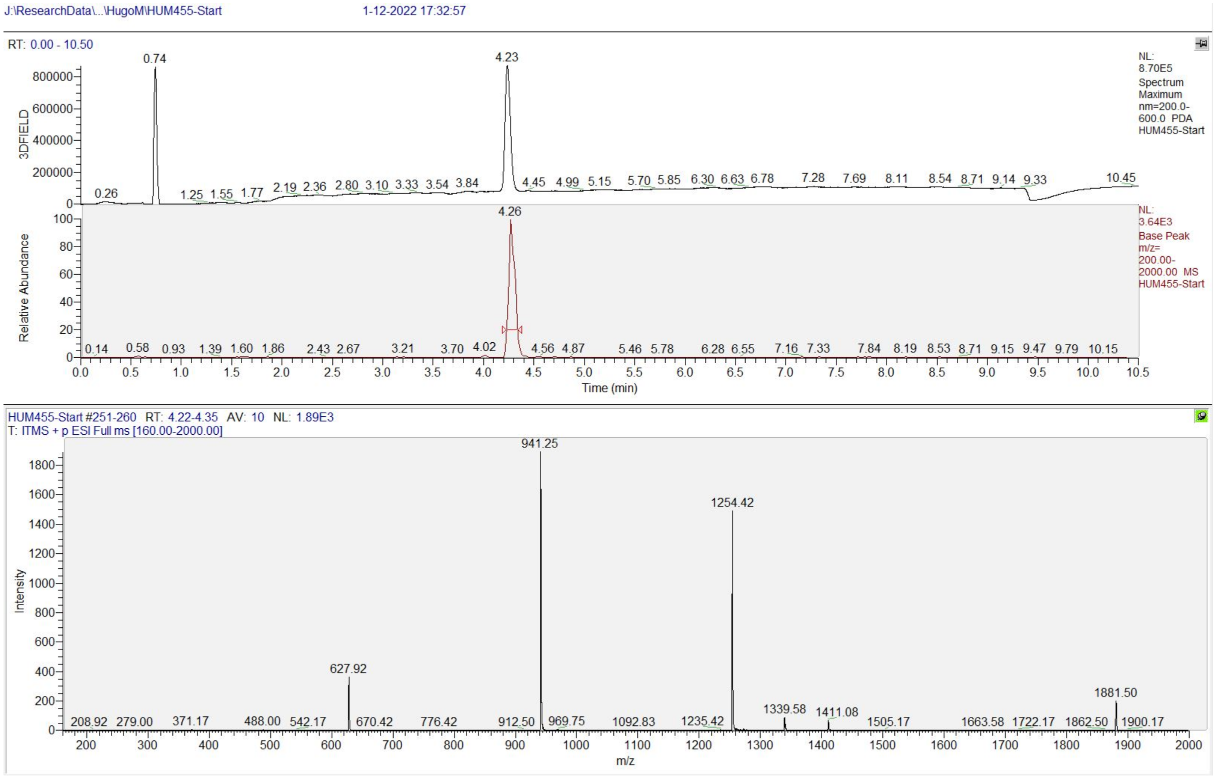

Figure S21: ^{1}HNMR, ^{31}PNMR & LCMS of compound **28**
